# *In silico* and *ex vivo* approaches indicate immune pressure on capsid and non-capsid regions of coxsackie B viruses in the human system

**DOI:** 10.1371/journal.pone.0199323

**Published:** 2018-06-20

**Authors:** Rhiannon Kundu, Robin Knight, Meenakshi Dunga, Mark Peakman

**Affiliations:** 1 Department of Immunobiology, School of Immunology, Infection and Inflammatory Disease, Faculty of Life Sciences and Medicine, King’s College London, London, United Kingdom; 2 Division of Diabetes and Nutrition, Faculty of Life Sciences and Medicine, King’s College London, United Kingdom; 3 NIHR Biomedical Research Centre, Guy’s and St Thomas’ NHS Foundation Trust and King’s College London, London, United Kingdom; University of British Columbia, CANADA

## Abstract

Coxsackie B Virus (CBV) infection has been linked to the aetiology of type 1 diabetes (T1D) and vaccination has been proposed as prophylaxis for disease prevention. Serum neutralising antibodies and the presence of viral protein and RNA in tissues have been common tools to examine this potential disease relationship, whilst the role of anti-CBV cytotoxic T cell responses and their targets have not been studied. To address this knowledge gap, we augmented conventional HLA-binding predictive algorithm-based epitope discovery by cross-referencing epitopes with sites of positive natural selection within the CBV3 viral genome, identified using mixed effects models of evolution. Eight epitopes for the common MHC class I allele HLA-A*0201 occur at sites that appear to be positively selected. Furthermore, such epitopes span the viral genome, indicating that effective anti-viral responses may not be restricted to the capsid region. To assess the spectrum of IFNy responses in non-diabetic subjects and recently diagnosed type 1 diabetes (T1D) patients, we stimulated PBMC *ex vivo* with pools of synthetic peptides based on component-restricted sequences identified *in silico*. We found responders were more likely to recognize multiple rather than a single CBV peptide pool, indicating that the natural course of infection results in multiple targets for effector memory responses, rather than immunodominant epitopes or viral components. The finding that anti-CBV CD8 T cell immunity is broadly targeted has implications for vaccination strategies and studies on the pathogenesis of CBV-linked diseases.

## Introduction

Coxsackie B viruses (CBVs) are a member of the diverse family of the picornaviridae. These viruses demonstrate tropism for human cells, entering by ligation of the coxsackievirus and adenovirus receptor. Whilst many human cases remain subclinical, CBVs can cause myocarditis and pancreatitis in neonates, the immunocompromised and the elderly. More recently, there is the suggestion that this family of viruses may play a role in the initiation or exacerbation of the pathogenesis of T1D, with some studies suggesting a particular association with CBV1 [1] and others CBV4 [2]. A nationwide cohort study in Taiwan indicated an increased risk of T1D in individuals presenting with enteroviral infection [3]. Furthermore, CBV proteins and RNA have been found in pancreatic sections from T1D patients studied post mortem [4]. Coxsackievirus RNA has also been detected in peripheral blood mononuclear cells (PBMCs) at onset of disease in children [5]. A recent meta-review of available data concluded that an overall increased risk of type 1 diabetes in association with coxsackievirus infection is indicated [6]. Preclinical mouse models of T1D support an association, as vaccination can ameliorate acceleration of disease onset caused by CBV1 viral infection in this setting [7]. Taken together, such findings have been proposed as support for vaccination against CBV as a prophylactic prevention to reduce or delay the onset of T1D.

The biology behind any potential causal association remains unclear, although there has been the suggestion of potential molecular mimicry between the diabetes-related autoantigen glutamic acid decarboxylase-65 and the CBV 2C protease [8]. CBV3 has been demonstrated to enhance autophagy [9], on which *in vivo* pathogenesis of infection in pancreatic acinar cells is dependent [10], and could therefore increase presentation of key disease-relevant autoantigens. Murine studies have suggested that CBV4 infection of pancreatic beta cells enhances phagocytosis by antigen presenting cells such as macrophages [11]. Combined with an inflammatory milieu associated with innate antiviral responses, this increase in autoantigen presentation may result in bystander activation or priming of autoreactive T cells to break tolerance and initiate or exacerbate disease. Cytotoxic T cell responses could act as major players in host cell death and pathogenesis, as has been shown to be the case for CBV viral myocarditis [12,13]. For these reasons, identification of molecular targets for CBV-specific CD8 responses would assist in assessing their role in pancreatic pathology, as well as being important for evaluating future vaccination approaches.

A previous approach to this question has been to predict CBV epitopes via a predictive algorithm coupled with prolonged in vitro co-culture of PBMCs with putative target peptides to elicit and measure T cell responses [14]. This approach has reduced specificity due to prolonged culture, and does not enable analysis of epitope regions that could be important for viral clearance. In the present study, we aimed to augment and expand epitope prediction and authentication for CBVs, by combining conventional MHC-binding algorithms with phylogenetic approaches to identify regions of the CBV genome evolving under positive selection to identify epitopes from immunogenic regions. Given the high degree of genetic similarity between enteroviruses, and the fact that T cell receptors can demonstrate high levels of cross-reactivity [15], we also utilised high density pre-culture to increase sensitivity [16] in short-term screening assays on human *ex vivo* blood samples to characterise potential CBV epitopes associated with memory to CBV infection.

## Materials and methods

### *In silico* identification of putative CBV epitopes

Coxsackievirus sequences were submitted to HLA-Restrictor [17] for identification of peptides with the potential to bind HLA-A*0201. To generate consensus sequences for analysis, all protein sequences greater than 1800 amino acids that returned for the search terms “coxsackievirus B”; “CBV” and “CVB” from Genbank were aligned by ClustalW pairwise alignment, edited and trimmed in MEGA6 [18]. Consensus sequence identity for each codon was identified using the Python package Bio.Align [19], taking 0.7 as a threshold for consensus identity at each amino acid site. Consensus sequences were generated for each serotype, as well as all coxsackie B viruses irrespective of serotype. To remove the effect of overrepresented CBV serotypes a consensus sequence of all serotype consensus sequences was generated as a “pan-CBV consensus”. The proportion of sequences containing each putative CBV HLA-A*0201 epitopes was assessed manually in MEGA6.

Identification of sites of positive selection was performed using 38 full nucleotide sequences of CBV3 available in GenBank. Sequences were aligned and trimmed in MEGA6 into frame and starting from the codon sequence beginning “MGAQVST…” Aligned nucleotide sequences were submitted to DataMonkey and checked for evidence of recombination by GARD. Sites evolving under positive selection were identified using MEME analysis, taking p-values <0.05 as significant [20].

Linear models were run in R (https://www.r-project.org/) and epitope radial plots generated in Strawberry Perl using the Circos package [21].

### Identification of IFNy responses by ELISpot

This study was approved by the Bromley Research Ethics Committee (reference 08/H0805). Venous blood was obtained from HLA-A*02:01 positive non-diabetic donors, or HLA-A*02:01 patients with recent-onset T1D with written consent, and PBMC isolated by density centrifugation as previously described [22]. PBMCs were cryopreserved to minimise variation during assay performance. Anti-CBV CD8 T cell responses were evaluated using a direct IFNγ ELISpot assay, in which PBMCs were thawed and rested for two days at high density (1.5 x10^7^/ml) in X-VIVO media + 5% human AB serum (Sigma). Cultured PBMCs were then recounted and plated at 3.3x10^6^/ml and 1x10^6^ cells stimulated with a pool of 4 peptides at 5μg/ml each final concentration (total final peptide concentration 20μg/ml) alongside diluent alone and viral peptide mix CEF (Mabtech) control conditions for three hours. Samples were transferred in triplicate to pre-coated and blocked IFNγ ELISpot plates (U-Cytech) and incubated for 24 hours to capture cytokine released. Cytokine release was identified as per the manufacturer’s instructions and plates counted using the Bio-sys Bioreader, as described previously [23–29]. Results are expressed as stimulation indices (SI) normalised to diluent-only control per 3.3 x 10^5^ cells (SIs of 2 and above were considered a positive response).

### Serum plaque neutralisation assay

Serum samples, diluted to 1 in 4 and 1 in 16 with serum free DMEM, were incubated with 200 pfu virus for 90 minutes before inoculation of a GMK monolayer in 96 well plates. After 1 hour with intermittent agitation, the inoculum was removed and an agarose-media plug layered onto the cells to prevent viral dissemination through the media. Assays were left for 48 hours for plaques to develop, before harsh fixation with Carnoy’s fix, washing, plug removal and staining with crystal violet. Plates were imaged using a Bio-sys Bioreader and restriction of virus-plaque formation quantified against virus-only inoculation controls. Virus strains used were: Coxsackievirus B1 (ATCC^®^ VR-28^™^)–Conn strain; Coxsackievirus B 2 (ATCC^®^ VR-29^™^)–Ohio strain; Coxsackievirus B3 (ATCC^®^ VR-30^™^)–Nancy strain; Coxsackievirus B4 (ATCC^®^ VR-184^™^)–J.V.B strain; Coxsackievirus B 5 (ATCC^®^ VR-185^™^)–Kentucky strain and Coxsackievirus B 6 (ATCC^®^ VR-155^™^)–Schmitt strain.

### Statistical analysis

Statistical analyses were performed in Prism 5, unless otherwise indicated. Tests used include Fisher’s exact and Student’s t test as indicated.

## Results

### Phylogenetic analysis of available whole-genome coxsackievirus B sequences

**Fig 1:**
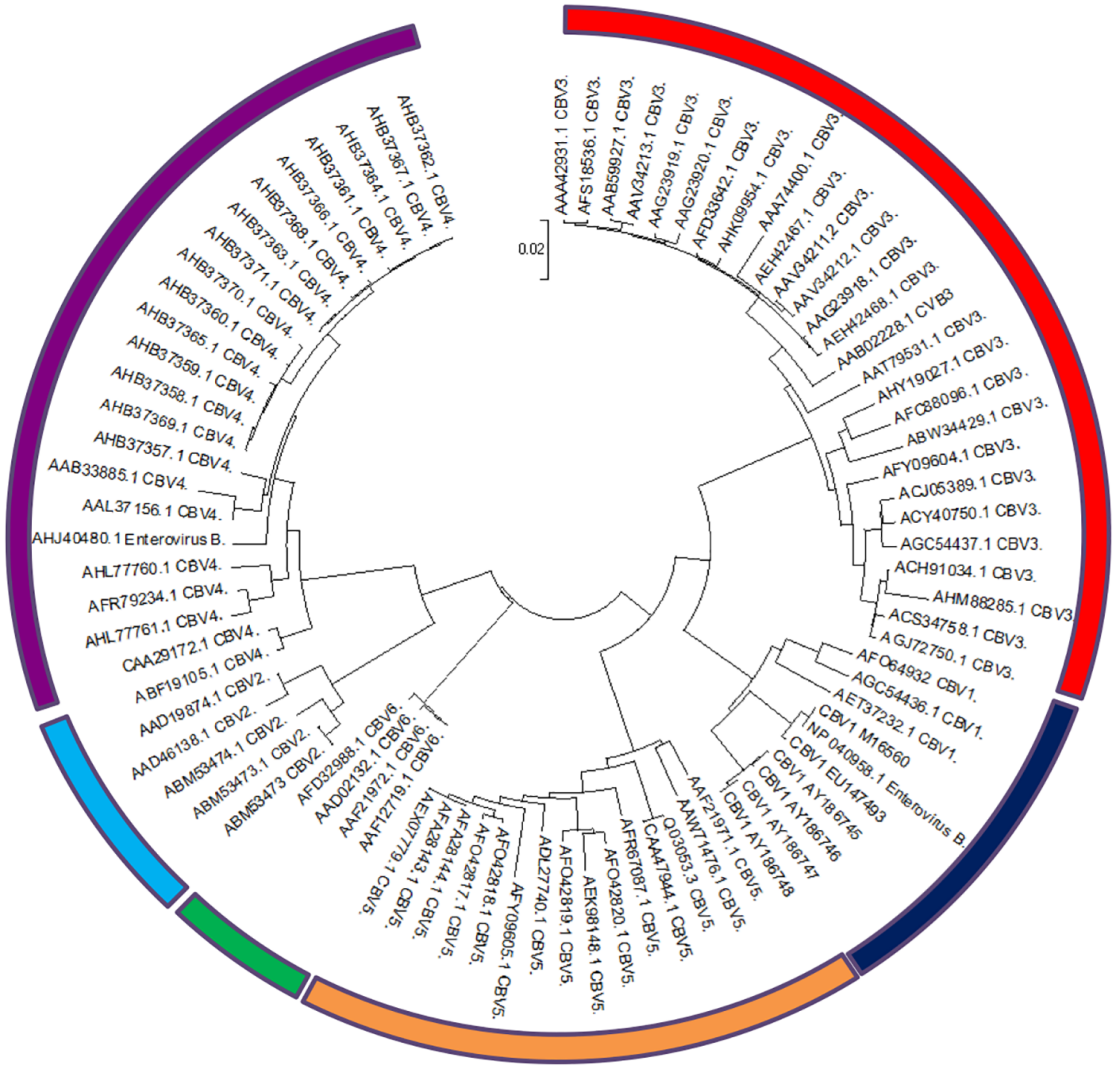
Phylogenetic analysis of available CBV sequences. Polyprotein sequences returned from Genbank for the terms “coxsackievirus B”; “coxsackie B virus”; “CBV”; “CVB”, listed in Table 1, were aligned using ClustalW pairwise alignment in Mega6. Genetic similarity between sequences and serotype clades in this alignment was assessed by generation of a maximum-likelihood phylogenetic tree. Sequences for CBV1 (10- highlighted in blue), CBV2 (7- light blue), CBV3 (29- red), CBV4 (25-purple), CBV5 (15-orange) and CBV6 (6- green) all clustered together with sequences for the same serotype, as would be expected from their labelling on Genbank.

We sought to identify potential MHC-I restricted epitope targets from coxsackievirus sequence data available on Genbank *in silico* by generating consensus sequences that could be interrogated for HLA-A*0201-binding epitopes. A search for sequences greater than 1800 amino acids in length on Genbank returned 9 sequences for CBV1, 4 for CBV2, 27 for CBV3, 23 for CBV4, 11 for CBV5 and 5 for CBV6 for alignment (S1 Table). This alignment was used to build a maximum likelihood phylogenetic tree and confirmed that sequences labelled as belonging to a particular serotype clustered together (Fig 1).

**Fig 2:**
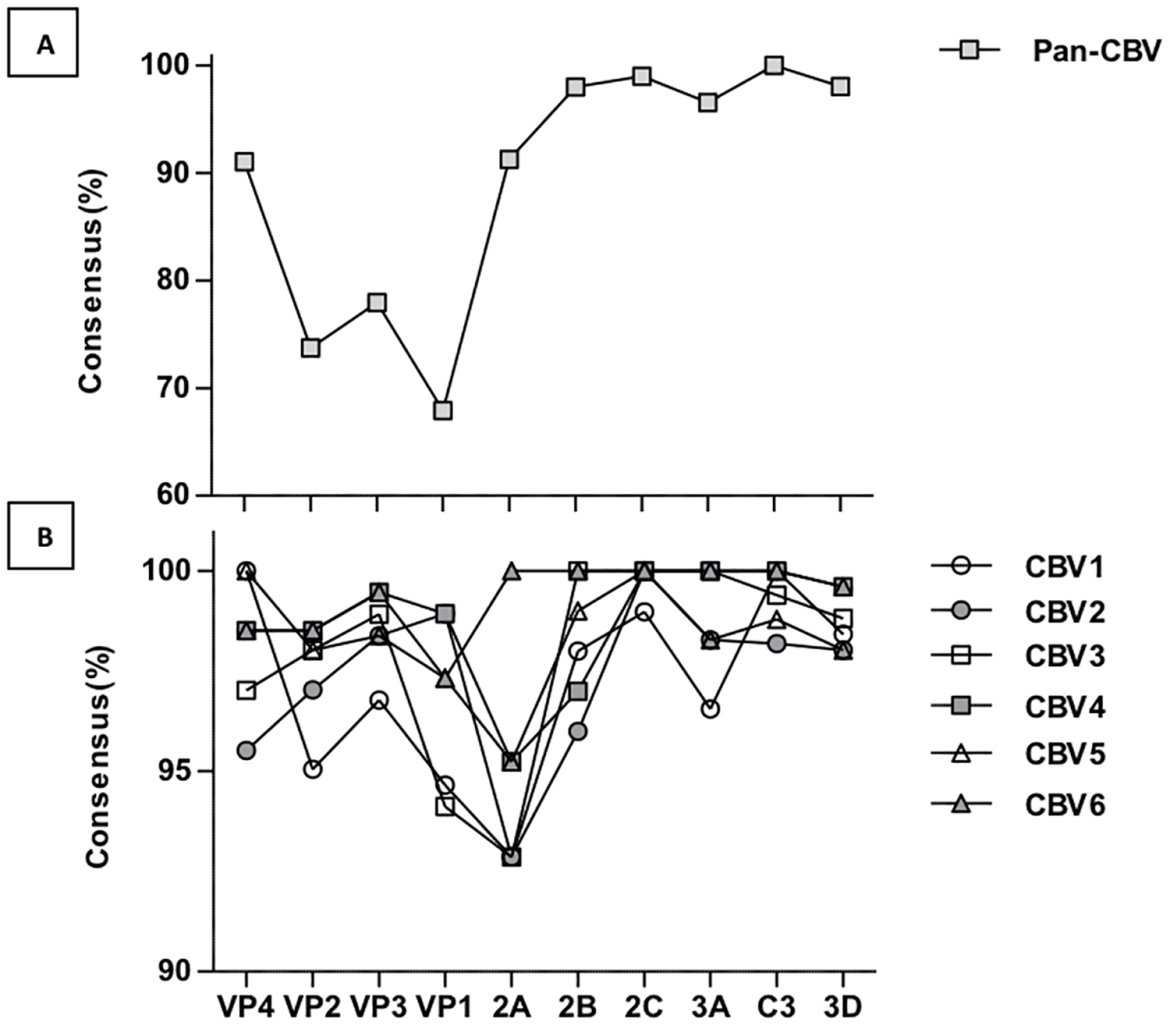
Conformation of viral component amino acid sequences to Pan-CBV and serotype-specific consensus sequences. To identify the protein components accounting for the greatest variance between sequences and serotypes used in our analyses, a pan-CBV consensus sequence was generated using Python Bio.Align package (using a threshold of 0.7) for all sequences for all serotypes, and the proportion of sequences conforming to this consensus sequence was determined for each amino acid site (A). Consensus sequences were also generated for each serotype, and the proportion of sequences within each specific serotype conforming to their consensus sequence was determined for each amino acid site for each viral protein (B).

We highlight the degree of consensus for each amino acid site in each polyprotein component within and across serotype sequences in Fig 2A. This figure shows the most conserved region between the serotypes by this measure to be the c3 protease region, with no site reaching below the threshold of 0.7 for the consensus level. Conversely, the most variable regions between the serotypes were the capsid proteins. VP1 was most variable, with only 67.91% of amino acids conforming to the pan-serotype consensus sequence in this region. Variation within each serotype was greatest in the 2A component of the polyprotein with approximately 92% conforming to the consensus sequence (Fig 2B).

### Identification of serotype specific MHC-I-restricted epitopes *in silico*

**Fig 3:**
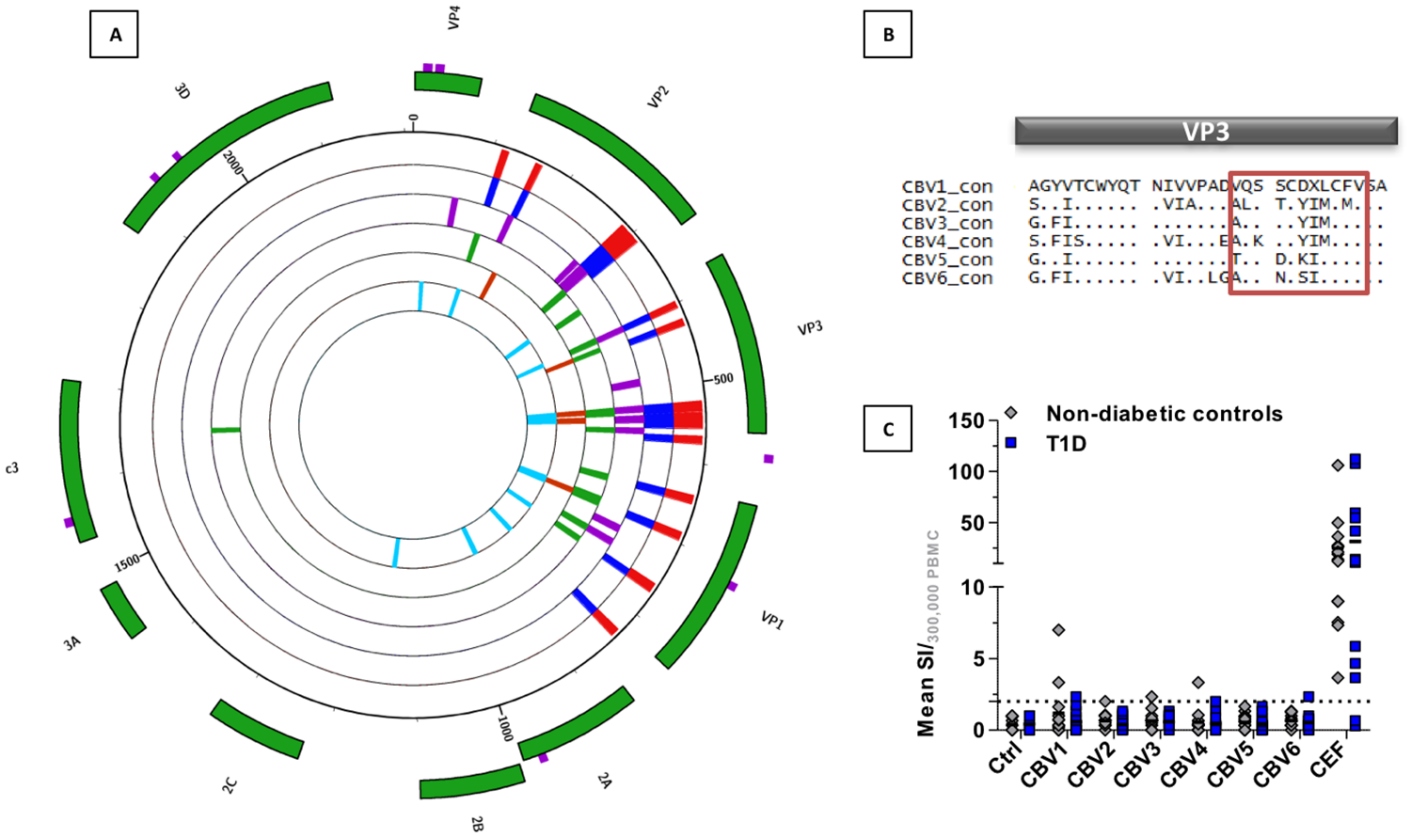
IFNy responses can be detected against epitopes at VP3 positions 538–548 in serotypes CBV1, 2, 3, 4 and 6. (A) Consensus sequences for CBV serotypes 1–6 were submitted to HLA-restrictor and returned putative HLA-A*0201 binding epitopes. The prevalence of these epitopes was assessed manually and serotype-unique sequences were presented in a radial plot. The radial tracks represent CBV serotypes 1–6, the outermost track of red histograms represents CBV1, blue CBV2, purple CBV3, green CBV4, rust CBV5 and aqua CBV6. The height of the histograms indicates the percentage of sequences within the indicated serotype that contain the epitope of interest at that site. The outer green tiles highlight the viral component. All serotypes of CBV had a putative HLA-A*0201 epitope at positions 538–548 within the VP3 region, which is highlighted in the alignment of consensus sequences presented in (B). (C) Cryopreserved PBMCs from non-diabetic controls (grey circles) and recently diagnosed T1D patients (blue squares) were thawed and pre-cultured at high density for 48 hours before stimulation with synthetic peptides (each at a final concentration of 5ug/ml) for the 538–548 epitopes in an IFNγ ELISpot. The results are normalised to DMSO controls and expressed as mean stimulation index (SI) per 3.3 x 10^5^ cells. We used a short-term ELISPot stimulation of 24 hours to ensure we did not prime naïve precursors and to minimise potential false positives from TCR promiscuity.

We submitted the consensus sequence for all coxsackie B virus serotypes to analysis by HLA-restrictor, for prediction of epitopes that could bind HLA-A*0201. We calculated the prevalence of each epitope returned from this analysis within the sequences of the alignment. The regions of 2A and 2B were significantly underrepresented in the epitopes found using HLA-restrictor, despite successfully generating a consensus sequence for this region. We therefore extracted coxsackievirus B sequences for this region from Genbank and generated a consensus sequence at a lower threshold of 0.4 to submit to HLA-restrictor to return more potential epitopes. We also identified serotype specific epitopes by submitting consensus sequences for each CBV serotype to HLA-restrictor from which exclusive epitopes were identified manually. These serotype specific epitopes were restricted almost entirely to the capsid regions of the virus (Fig 3A, outer to inner track of histograms: CBV1, CBV2, CBV3, CBV4, CBV5, CBV6). This analysis demonstrates that while the level of consensus between different serotypes is low in the capsid, this region appears to be highly immunogenic across serotypes. At position 538 within the VP3 capsid region, we identified a different HLA-A*0201 binding epitope within each serotype (Table 1 and Fig 3B).

**Table 1:** Synthetic peptides used to detect serotype-specific anti-CBV responses in ELISpot assays.

Serotype	Sequence	Predicted Affinity (nM)
**CBV1**	VQSSCDVLCFV	39
**CBV2**	ALSTCYIMCMV	11
**CBV3**	AQSSCYIMCFV	11
**CBV4**	AQKSCYIMCFV	361
**CBV5**	TQSDCKILCFV	70
**CBV6**	AQSNCSILCFV	27

These epitopes indicate that VP3 remains immunogenic despite substantive change during the differentiation of the serotypes, and could be used to detect serotype-specific MHC class I-restricted immunity. Such tools would be particularly useful in investigating the potential role of viral infection in T1D, as infection with CBV1 [1] or CBV4 [30] has been purported as a mechanism driving onset of disease. We therefore utilised synthetic peptides designed from VP3 sequences to stimulate PBMC from healthy controls and recently diagnosed patients with T1D in short term-ELISpot assays to detect serotype-specific MHC-I restricted IFNγ responses (Fig 3C). We detected low amplitude responses to all peptides, except for the CBV5-derived sequence TQSDCKILCFV. Despite the low predicted affinity for the CBV4-derived epitope AQKSCYIMCFV, we detected two responders from the 24 individuals tested. The CBV1 sequence VQSSCDVLCFV had the greatest number of responders, with two controls and one T1D returning a positive IFNγ response. Of the five ELISpot responders, all had either strong or weak serum neutralising antibody responses to the serotype they demonstrated a positive ELISpot to (S2 Table). They, like the ELISpot non-responders also demonstrated neutralisation against most other serotypes. These ELISpot responses suggest that the epitopes we identified *in silico* at VP3 538–548 can be used to screen for CBV serotype-biased MHC-I restricted immune responses.

### Mixed effects model of evolution identifies sites of positive selection within putative anti-CBV HLA-A*02:01 binding epitopes

**Fig 4:**
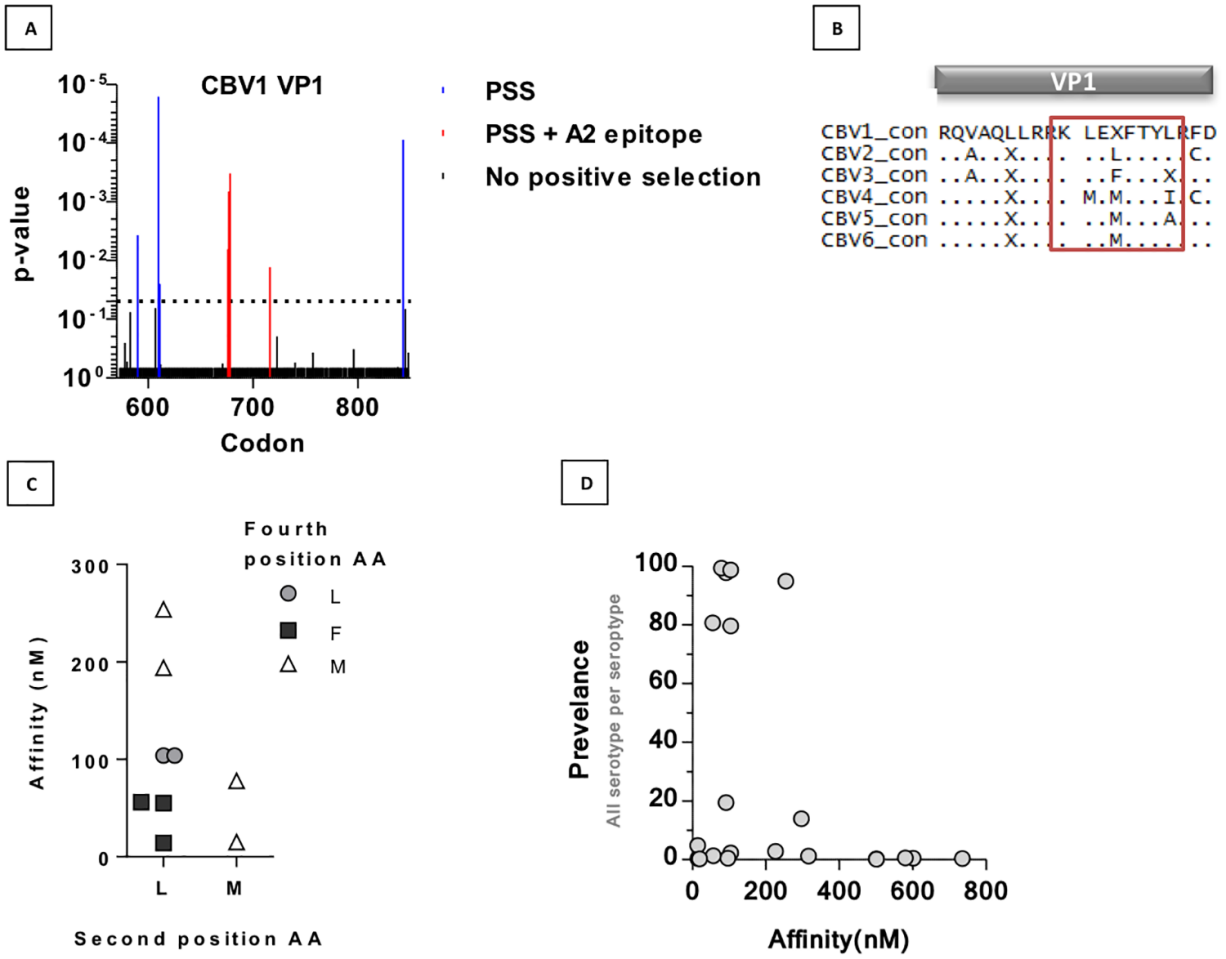
MEME analysis of CBV1 VP1 sequences reveal putative immunogenic regions across serotypes of CBV. (A) Trimmed and edited CBV1 VP1 sequences from Table 4 were aligned by Bio.Align in Python and submitted to Datamonkey to identify sites of positive selection by mixed effects model of evolution. The p-value for the significance of which the amino acid site is evolving under positive selection within the model is represented. (B) Epitopes present at VP1 684–692 are highlighted within an alignment of serotype consensus sequences. (C) The predicted affinity for all potential sequences at VP1 684–692 are plotted against the amino acid identity for positions 2 and 4 in the 8-mer. (D) Prevalence of each sequence present at sites 682–694 for all serotypes was determined and plotted against their predicted binding affinity for HLA-A*0201, determined by HLA-restrictor.

To examine the role of positive selection, potentially from the immune system, in driving variation in coxsackie B viruses we used Datamonkey’s web-based application [20] to identify codons that had significantly more nonsynonymous mutations within the population using a mixed effects model of evolution (MEME). There is particular interest in generating a CBV1 capsid based vaccine for clinical use [7], so we performed a MEME analysis using sequences returned from Genbank for the VP1 region of CBV1 (S3 Table) in order to identify sites that could be generating key anti-viral responses. We identified eight sites of positive selection within VP1 (S4 Table), including three consecutive sites at codons 676, 677 and 678 (Fig 4A). These consecutive sites coincided with a putative HLA-A*0201 binding epitope present in 40% CBV1 sequences (KLELFTYL 675–682) with a predicted binding affinity of 104nM. We found that HLA-restrictor predicted an HLA-A*0201 binding epitope at these positions for all serotypes of CBV (Fig 4B). The epitopes were 8-mers that differed in amino acid identity at positions 2, 4 and 8. We catalogued all available sequences for these sites in VP3 across all six serotypes and found that amino acid identities at positions 2 and 4 had a major impact on their predicted binding affinity for HLA-A*02:01 (Fig 4C). We also compared the prevalence of each sequence with its predicted binding affinity for HLA-A*0201 (Fig 4D). We found that apart from the sequence for CBV5, which was present in 95% of sequences analysed and had a predicted binding affinity of 254nM, sequences with no or a low affinity for binding HLA-A*0201 were present at lower frequencies. Similarly, the few epitopes present with a strong predicted binding affinity (<55nM) were also present at low frequencies. These observations suggest that despite immune pressure in this region, non-synonymous substitutions that alter the biophysical properties of this region too extremely are disadvantageous to the virus, as are substitutions that increase affinity of binding to a common HLA allele such as HLA-A*0201, making this region of great interest for vaccination strategies against CBV1 and other serotypes of CBV.

**Fig 5:**
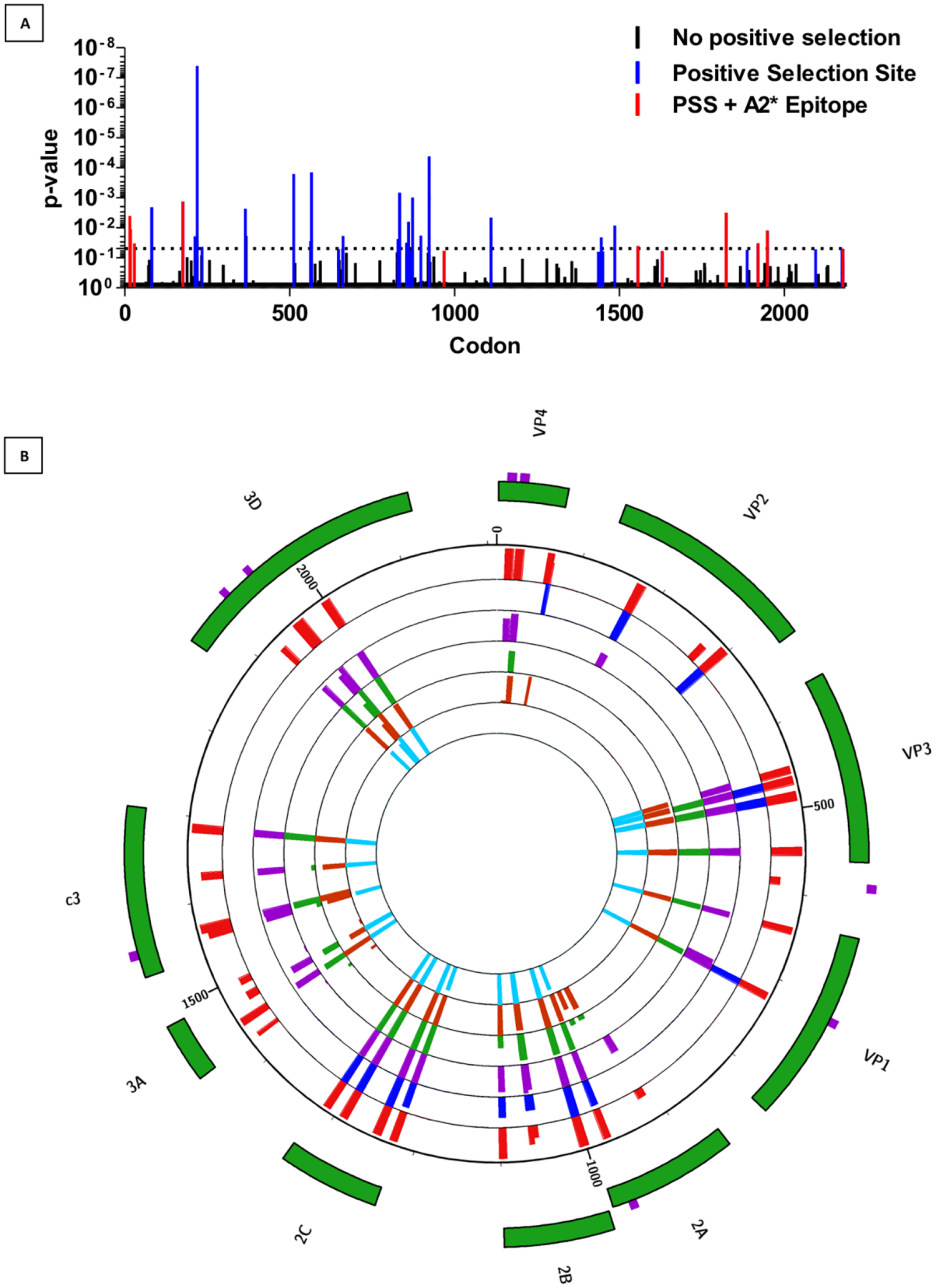
Circos plot indicating the prevalence of viral protein component epitopes across CBV serotypes. (A) Trimmed and edited CBV3 sequences from Table 1 were aligned by Bio.Align in Python and submitted to Datamonkey to identify sites of positive selection by mixed effects model of evolution. The p-value for the significance of which the amino acid site is evolving under positive selection is represented. (B) The percentage of sequences for each serotype containing each epitope was calculated manually and presented as histograms in a Circos plot. The radial tracks represent CBV serotypes 1–6, the outermost track of red histograms represents CBV1, blue CBV2, purple CBV3, green CBV4, rust CBV5 and aqua CBV6. The height of the histograms indicates the percentage of sequences within the indicated serotype that contain the epitope of interest at that site. The outer green tiles highlight the viral component. The outermost purple highlights indicate the presence of a site of positive selection within the CBV3 genome.

To date, most approaches in assessing the role of coxsackievirus infection in accelerating onset of T1D have centred on hypotheses of a diabetogenic serotype, or even strain [31] of CBV. We sought to test an alternate hypothesis that the targets of anti-CBV immune responses differ between non-diabetic control subjects and patients with T1D. To do so we generated pools of synthetic peptides derived from each viral protein of CBV, to use as stimuli in *ex vivo* PBMC assays. To prioritise inclusion of epitopes from putatively immunogenic regions, we performed a MEME analysis for full length sequences of CBV3, the most highly-represented serotype on GenBank, to identify sites under positive selection. We hypothesised that most sites under positive selection would be within the capsid. Interestingly, in addition to 16 sites within the capsid, we identified a further 20 sites in other viral components that appeared to be evolving under positive selection (S5 Table and Fig 5A). Five of the capsid and four of the non-capsid sites of positive selection lay within a predicted HLA-A*0201 epitope. To assess the presence of CD8 memory responses in human peripheral blood against coxsackievirus epitopes identified by our *in silico* analyses, we selected pools of four peptides for each viral component to use in *ex vivo* stimulation assays. Epitopes derived from regions that contained a site of positive selection in our previous MEME analysis were prioritised and additional peptides with the highest predicted HLA-A2-binding affinity (no weaker than 150nM) to make up pools of four peptides per viral component. In the case of VP4, there were only two potential epitopes returned using HLA-restrictor. We therefore submitted sequences to SYFPEITHI[32] which returned two additional peptides to bring the VP4 pool up to four. These were taken forward for functional studies (Table 2 and Fig 5B).

**Table 2:** Synthetic peptides used to detect anti-CBV T cell responses in ELISpot assays.

ID	Region	Start	Stop	Length	PSS	PSS AA	Sequence	PredictedAffinity (nM)*
**220**	VP4	10	19		18	N	TGAHETGLNA	
**221**	VP4	22	31		30	I	NSIIHYTNIN	
**86**	VP4	60	68	9	-	-	VMIKSMPAL	36
**71**	VP4	60	68	9	-	-	IMIKSMPAL	29
**25**	VP2	169	179	11	117	I	YLGRTGYTIHV	10
**14**	VP2	271	279	9	-	-	LVMPYINSV	7
**12**	VP2	291	298	8	-	-	TLMIIPFV	6
**42**	VP2	294	301	8	-	-	MIIPFVPL	17
**3**	VP3	450	458	9	-	-	FMFCGSAMA	3
**5**	VP3	462	471	10	-	-	FLLAYSPPGA	4
**22**	VP3	480	490	11	-	-	AMLGTHVIWDV	9
**28**	VP3	545	554	10	-	-	FVSACNDFSV	11
**222**	VP1	582	590		583/590	MV	AMVRVADTV	60
**66**	VP1	639	646	8	-	-	FLCRSACV	26
**127**	VP1	713	721	9	721	V	ILTHQIMYV	8
**121**	VP1	718	727	10	-	-	IMYVPPGGPV	88
**151**	2A	910	920	11	-	-	IIARCQCTTGV	96
**134**	2A	932	942	11	-	-	VVFEGPGLVEV	17
**135**	2A	952	960	9	-	-	YQTHVLLAV	17
**223**	2A	970	978		975	E	GILRCEHGV	453
**119**	2B	997	1007	11	-	-	WLEDDAMEQGV	80
**46**	2B	1053	1062	10	-	-	ALVKIISALV	19
**61**	2B	1056	1064	9	-	-	KIISALVIV	24
**120**	2B	1093	1101	9	-	-	SQYYGIPMA	83
**100**	2c	1222	1230	9	-	-	LLLHGSPGA	46
**24**	2c	1242	1251	10	-	-	SLAEKLNSSV	10
**11**	2c	1285	1294	10	-	-	SLFCQMVSSV	6
**33**	2c	1308	1316	9	-	-	ILFTSPFVL	13
**36**	3a	1441	1448	8	-	-	ALFQGPPI	14
**90**	3A	1447	1455	9	-	-	AIADLLKSV	39
**74**	3A	1470	1479	10	-	-	WLVPEINSTL	31
**47**	3A	1490	1498	9	-	-	CLQAITTFV	20
**67**	c3	1556	1565		1564	T	AMMKRNSSTV	27
**15**	c3	1563	1573	11	-	-	TMLGIYDRWAV	7
**9**	c3	1626	1634	9	-	-	FLAKEEVEV	6
**45**	c3	1682	1691	10	-	-	MLMYNFPTRA	17
**224**	3D	1923	1930		1954	D	AFHQNPGIVT	42
**225**	3D	1953	1960		2184	T	LDGHLIAFD	16
**43**	3D	1960	1967	8	-	-	SLSPVWFA	17
**89**	3D	1995	2005	11	-	-	YIDYLCNSHHL	38

*Predicted affinity from HLA-restrictor

### *Ex vivo* IFNy responses can be detected against capsid and non-*capsid*

**Fig 6:**
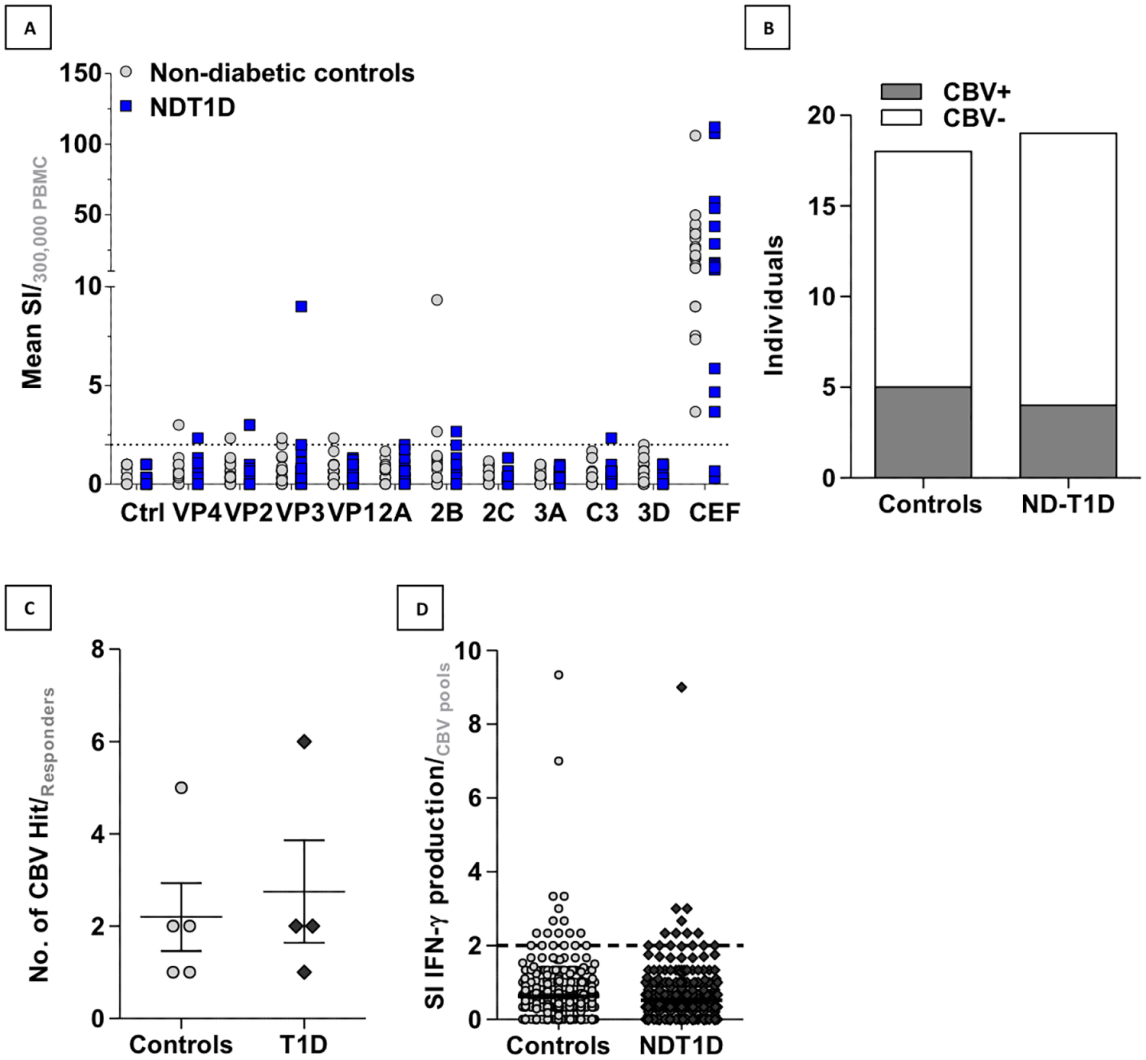
IFNγ responses to CBV viral component peptide pools in non-diabetic controls and recently-diagnosed T1D patients. PBMC from non-diabetic and recently diagnosed -T1D patients were pre-cultured at high density for 48 hours before stimulation with pools of CBV and control peptides for 18 hours in an IFNγ ELISpot assay. Spots are reported as mean stimulation index normalised to DMSO control for 3.3 x 10^5^cells. (A) Stimulation indices for cohort responses to viral component CBV pools and serotype specific CBV pools (B) are presented. (C) The number of individuals demonstrating positive IFNγ ELISpot responses to any CBV derived peptide pool for ND-T1Ds and non-diabetic controls. (D) The number of positive hits for CBV peptide pools in responders is plotted for control and recently-diagnosed T1D cases.

As several studies have suggested CBV infection may precede T1D diagnosis, we hypothesised that we would be more likely to detect CD8 memory responses against CBV peptide pools in recently diagnosed patients with T1D than in non-diabetic controls. To test for pre-existing cellular immunity to CBVs, cryopreserved PBMCs were thawed and pre-cultured at high density for 48 hours before stimulation with CBV peptide pools designed in the previous section in a short-term IFNγ ELISpot assay. We observed responses to the capsid-derived peptide pools, though there were also multiple responders to the 2B component pool (Fig 6A and S6 Table). We detected positive IFNγ responses to CBV peptide pools in 4/18 non-diabetic controls and 4/19 recently diagnosed patients (no significant difference in distribution by Fisher’s exact test (Fig 6B)). Responders generally exhibited IFNγ responses to more than one CBV peptide pool, and there was no significant difference in the number of pools that controls and patients responded against (Fig 6C). In addition, there was no significant difference in the consolidated stimulation indices for CBV responses between the two groups (Fig 6D). We found that the presence of serum neutralising antibodies, as determined by plaque neutralisation assay, was largely equivalent in cases and controls of serum samples tested from this cohort (S1A Fig). There were no significant differences in time since diagnosis for patient responders and non-responders to the CBV pools (S1B Fig), nor were there any differences between the two groups of CBV-responders in magnitude of IFNy responses to a positive control viral peptide mix (S1C Fig) and age (S1D Fig). Overall, these data do not indicate an association of anti-CBV immunity with T1D. However, this *ex vivo* data does suggest that CBV infection can generate IFNγ responses against not only capsid proteins, but also non-structural proteins such as proteases 2A and 2B, and polymerase 3D. These data support our suggestion that the positive selective pressure we observed outside of the capsid region of CBV may be immune mediated and specifically related to the potential for viral sequences to bind such a common MHC class I allele as HLA-A*0201.

## Discussion

Observational studies linking infection with coxsackievirus (and other enteroviruses) to T1D [3,6,33–36] have given rise to a number of experimental studies in preclinical models of the disease that support a role for infection in exacerbating pathological processes, an effect which vaccination against the virus can negate [7]. These observations have generated interest in the possibility of prophylactic vaccination against potentially diabetogenic CBV strains to reduce or delay the development of T1D [37]. Characterisation of CD8 epitope targets associated with viral infection would provide useful tools to monitor CBV responses in cohort studies and track the efficacy of vaccination approaches. The present study shows that many sites throughout the CBV3 genome appear to be evolving under positive selection by the immune system. This finding suggests a key role for immune responses outside of neutralising antibodies, which are typically considered the most important component of the anti-CBV response due to the predisposition of agammaglobulinemic patients to enteroviral encephalitis [38,39]. As neutralising antibodies target capsid, immune selective pressure on sites within non-structural proteins most likely derive from antigens generated during intracellular phases of the viral life cycle, or from internalisation of pathogen components by professional antigen-presenting cells. These antigens could prime either helper or cytotoxic T cell responses. These findings imply that successful generation of these cellular responses must be integral to the clearance of the virus for such selective pressure on non-structural components to be apparent. This new insight within the human system provides a series of potential targets through which to monitor immune responses to infection and vaccination, as well as holding implications for vaccine design.

By utilising high density pre-culture [16] before a short-term culture IFNy ELISpot assay to test epitopes on human *ex vivo* PBMCs, we were able to detect more specific memory responses than would be possible in a long term culture assay, in which priming and cross-reactivity have the potential to skew results. In this small-scale study, there was no single immunodominant epitope identified and we detected only low amplitude responses in a minority of individuals tested. A technical issue to account for this could be the use of pooled peptides, as in our laboratory we have observed lower SIs in response to a pool than when the individual peptides have been tested in the same donor. Aside from this, there are several biological interpretations that could be made for this result. First, is that successful clearance of enteroviruses is dependent on broad low level T cell responses, consistent with our *in silico* observations that sites across capsid and non-capsid regions evolve under immune pressure. Second, epitopes that contain amino acids from sites evolving under positive selection may differ across individuals, so we may be identifying a minority of responses that target this region of the genome. Third, the findings are consistent with our current understanding of the biology of CBVs. Viruses associated with chronic and recurring infections such as human cytomegalovirus [40], human Epstein-Barr virus [41], and influenza viridae [42], tend to focus cellular immune responses onto immunodominant regions, which are reprimed through repeated antigen exposure through-out life. What we have observed is a broad and low level specificity of CVB responses suggestive of resolved infection and long term protection, which is known to be predominantly maintained throughout life by neutralizing antibody. As a next step to studying the most plausible of these explanations that are also experimentally testable, we would propose a study in children, in whom CVB infection is likely to be more immediate and therefore characterised by a higher amplitude of cellular immune memory responses. However, given the blood volume requirements, such a study would need to be focused onto a discrete panel of epitopes informed by studies such as this one. These contrasting findings between types of virus immunity also imply that CBV infection does not have a chronic phase, or at least not one during which there is exposure to CD8 responses. Finally, CBV3 in animal models has been shown to actively suppress formation of CD8 memory responses [43], potentially by effecting dendritic cell stimulatory capacity [44]. In this light, our data may support the view that this also occurs in the human system for CBV3 and other serotypes, as all individuals tested had neutralising antibodies against at least two serotypes of CBV, the majority restricting plaque formation by four or more serotypes (S2 Table), but only 20% demonstrated positive ELISpots for CBV-derived MHC-I restricted epitopes.

*Ex vivo* analyses showed a diverse spread of epitope targets across individuals when positive ELISpot responses against CBV peptide pools were detected. Responders generally demonstrated low amplitude IFNy ELISpots to more than one pool of CBV-derived peptides, indicating the generation of multiple-specificities of CD8 memory over the course of an infection. In relation to our secondary objective, to examine potential differences in CD8 responses to CBV in patients with T1D compared with non-diabetic controls, we did not observe any differences in the prevalence or breadth of responses. These results should be interpreted cautiously at this stage since our subject numbers were small and due to the large volume of blood required for the *ex vivo* ELISpot screens we studied adult rather than childhood subjects (in whom enterovirus infections are more frequent). We have also studied subjects at a late stage of a disease that has a long prodrome, with the risk that we have missed important early events. Our study provides a systematic analysis of CBV epitopes that should be of use in designing more extensive studies at earlier disease stages, to address these issues.

## Supporting information

S1 Fig Serum neutralising antibody responses and other key confounding parameters were equivalent in the two arms of the cohort study. (A) For individuals where samples were available, serum neutralisation of plaque formation by coxsackievirus serotypes 1–6 was assayed as an indicator of serum neutralising antibodies, and so previous virus exposure. Weak neutralisation was defined as a greater than 50% reduction in the number of plaques formed using a 1 in 4 dilution of serum, whilst strong neutralisation was defined by a greater than 50% reduction in the number of plaques using 1 in 16 dilution of serum. The percentage of samples with neutralising capacity for each serotype is presented for recently diagnosed T1Ds (n = 10 for CBV1 and CBV2, n = 11 for CBV3- 6) and non-diabetic controls (n = 9 for CBV1, n = 7 for CBV2 and n = 10 for CBV3-6). Months since diagnosis (B); SI per 3.3 x 10^5^ PBMC for positive control CEF (C) and age (D) are plotted for recently-diagnosed T1Ds against non-diabetic controls and CBV-responders and non-responders. (E) Mean SIs for individuals responding to any CBV peptide pool are plotted against the number of positive hits for that individual. Dark grey points indicate ND-T1Ds and light grey points indicate non-diabetic controls.
(TIF)

S1 Table Sequences used for serotype specific and pan-serotype epitope prediction. Sequences returned from Genbank for the queries “CBV”, “CVB” “Coxsackievirus B” and “Coxsackie B Virus” were collated for use in *in silico* epitope prediction approaches.
(DOCX)

S2 Table Serotype specific ELISpot responses and serum plaque formation neutralisation assay results. Cryopreserved PBMCs were thawed and rested for two days at high density (1.5 x10^7^/ml) in X-VIVO media + 5% human AB serum (Sigma). Cultured PBMCs were then recounted and plated at 3.3x10^6^/ml and 1x10^6^ cells stimulated with indicated peptides at 5μg/ml each final concentration, alongside diluent alone and viral peptide mix CEF (Mabtech) control conditions for three hours. Samples were transferred in triplicate to pre-coated and blocked IFNγ ELISpot plates (U-Cytech) and incubated for 24 hours to capture cytokine released. Cytokine release was identified as per the manufacturer’s instructions and plates counted using the Bio-sys Bioreader. The mean IFNy SI per 3.3x10^5^ cells of three replicate wells and total spots per 10^6^ are presented for ELISpot assays against serotype specific epitope at position 538–548. Results from the neutralisation of CBV plaque formation by serum assays are also presented; strong responses were taken as those sera reducing plaque formation by 50% with a 1 in 16 dilution of serum, and weak responses were taken as those sera that reduced plaque formation by 50% with a 1 in 4 dilution of serum when compared to virus only controls.
(DOCX)

S3 Table Sequences used for CBV1 VP1 MEME. Sequences returned from Genbank for the queries “CBV1”, “CVB1”, “Coxsackievirus B1” and “Coxsackie B Virus 1” were collated for use in *in silico* epitope prediction approaches.
(DOCX)

S4 Table Sites of positive selection identified in CBV1 VP1 by MEME. The sites of positive selection identified in CBV1 VP1 sequences available on Genbank are presented, along with the predominant amino acid present at that site and any HLA-A*02:01 binding epitopes contained within the site.
(DOCX)

S5 Table Sites of positive selection identified in CBV3 by MEME. Full length sequences returned from Genbank for the queries “CBV3”, “CVB3”, “Coxsackievirus B3” and “Coxsackie B Virus 3” and used to identify sites evolving under positive selective pressure using Datamonkey’s mixed effects model of evolution packages. Sites that were identified as evolving under positive selection are listed, along with HLA-A*02:01 binding epitopes that incorporate that site.
(DOCX)

S6 Table Raw data for viral component pool ELISpot assays. Cryopreserved PBMCS were thawed and precultured at high-density for 48 hours as preparation for ELISpot assay. PBMCs were then recounted and plated at 3.3x10^6^/ml and 1x10^6^ cells stimulated with a pool of 4 peptides at 5μg/ml each final concentration (total final peptide concentration 20μg/ml) alongside diluent alone and viral peptide mix CEF (Mabtech) control conditions for three hours. Samples were transferred in triplicate to pre-coated and blocked IFNγ ELISpot plates (U-Cytech) and incubated for 24 hours. Cytokine release was identified as per the manufacturer’s instructions and plates counted using the Bio-sys Bioreader. The mean IFNy SI per 3.3x10^5^ cells of three replicate wells and total spots per 10^6^ are presented for ELISpot assays against viral component specific peptide pools.
(DOCX)

## References

[1] Oikarinen S, Tauriainen S, Hober D, Lucas B, Vazeou A, Sioofy-Khojine A, et al Virus antibody survey in different european populations indicates risk association between coxsackievirus B1 and type 1 diabetes. Diabetes. 2014;63: 655–662. doi: 10.2337/db13-0620 24009257 10.2337/db13-0620

[2] Chehadeh W, Weill J, Vantyghem MC, Alm G, Lefèbvre J, Wattré P, et al Increased level of interferon-alpha in blood of patients with insulin-dependent diabetes mellitus: relationship with coxsackievirus B infection. J Infect Dis. 2000;181: 1929–1939. doi: 10.1086/315516 10837172 10.1086/315516

[3] Lin H-C, Wang C-H, Tsai F-J, Hwang K-P, Chen W, Lin C-C, et al Enterovirus infection is associated with an increased risk of childhood type 1 diabetes in Taiwan: a nationwide population-based cohort study. Diabetologia. 2015;58: 79–86. doi: 10.1007/s00125-014-3400-z 25335440 10.1007/s00125-014-3400-z

[4] Richardson SJ, Willcox a., Bone a. J, Foulis a. K, Morgan NG. The prevalence of enteroviral capsid protein vp1 immunostaining in pancreatic islets in human type 1 diabetes. Diabetologia. 2009;52: 1143–1151. doi: 10.1007/s00125-009-1276-0 19266182 10.1007/s00125-009-1276-0

[5] Yin H, Berg A-K, Tuvemo T, Frisk G. Enterovirus RNA is found in peripheral blood mononuclear cells in a majority of type 1 diabetic children at onset. Diabetes. 2002;51: 1964–1971. doi: 10.2337/diabetes.51.6.1964 12031987 10.2337/diabetes.51.6.1964

[6] Yeung W, Rawlinson W, Craig M. Enterovirus infection and type 1 diabetes mellitus: systematic review and meta-analysis of observational molecular studies. Bmj. 2011; 1–9. doi: 10.1136/bmj.d35 21292721 10.1136/bmj.d35 PMC3033438

[7] Larsson PG, Lakshmikanth T, Laitinen OH, Utorova R, Jacobson S, Oikarinen M, et al A preclinical study on the efficacy and safety of a new vaccine against Coxsackievirus B1 reveals no risk for accelerated diabetes development in mouse models. Diabetologia. 2014;58: 346–354. doi: 10.1007/s00125-014-3436-0 25370797 10.1007/s00125-014-3436-0

[8] Varela-Calvino R, Skowera A, Arif S, Peakman M. Identification of a Naturally Processed Cytotoxic CD8 T-Cell Epitope of Coxsackievirus B4, Presented by HLA-A2.1 and Located in the PEVKEK Region of the P2C Nonstructural Protein. J Virol. 2004;78: 13399–13408. doi: 10.1128/JVI.78.24.13399-13408.2004 15564450 10.1128/JVI.78.24.13399-13408.2004 PMC533958

[9] Shi X, Chen Z, Tang S, Wu F, Xiong S, Dong C. Coxsackievirus B3 infection induces autophagic flux, and autophagosomes are critical for efficient viral replication. Arch Virol. 2016;161: 2197–2205. doi: 10.1007/s00705-016-2896-6 27224983 10.1007/s00705-016-2896-6

[10] Alirezaei M, Flynn CT, Wood MR, Whitton JL. Pancreatic acinar cell-specific autophagy disruption reduces coxsackievirus replication and pathogenesis in vivo. Cell Host Microbe. Elsevier Inc.; 2012;11: 298–305. doi: 10.1016/j.chom.2012.01.014 22423969 10.1016/j.chom.2012.01.014 PMC3308121

[11] Horwitz MS, Ilic A, Fine C, Balasa B, Sarvetnick N. Coxsackieviral-mediated diabetes: Induction requires antigen-presenting cells and is accompanied by phagocytosis of beta cells. Clin Immunol. 2004;110: 134–144. doi: 10.1016/j.clim.2003.09.014 15003810 10.1016/j.clim.2003.09.014

[12] Opavsky M a, Penninger J, Aitken K, Wen WH, Dawood F, Mak T, et al Susceptibility to myocarditis is dependent on the response of alphabeta T lymphocytes to coxsackieviral infection. Circ Res. 1999;85: 551–558. doi: 10.1161/01.RES.85.6.551 10488058 10.1161/01.res.85.6.551

[13] Liu W, Dienz O, Roberts B, Moussawi M, Rincon M, Huber S a. IL-21R expression on CD8+ T cells promotes CD8+ T cell activation in coxsackievirus B3 induced myocarditis. Exp Mol Pathol. Elsevier Inc.; 2012;92: 327–33. doi: 10.1016/j.yexmp.2012.03.009 22465422 10.1016/j.yexmp.2012.03.009 PMC3354021

[14] Weinzierl AO, Rudolf D, Maurer D, Wernet D, Rammensee H-G, Stevanović S, et al Identification of HLA-A*01- and HLA-A*02-restricted CD8+ T-cell epitopes shared among group B enteroviruses. J Gen Virol. 2008;89: 2090–7. doi: 10.1099/vir.0.2008/000711-0 18753217 10.1099/vir.0.2008/000711-0

[15] Wooldridge L, Ekeruche-Makinde J, Van Den Berg H a., Skowera A, Miles JJ, Tan MP, et al A single autoimmune T cell receptor recognizes more than a million different peptides. J Biol Chem. 2012;287: 1168–1177. doi: 10.1074/jbc.M111.289488 22102287 10.1074/jbc.M111.289488 PMC3256900

[16] Wegner J, Hackenberg S, Scholz CJ, Chuvpilo S, Tyrsin D, Matskevich AA, et al High-density preculture of PBMCs restores defective sensitivity of circulating CD8 T cells to virus- and tumor-derived antigens. Blood. 2015;126: 185–194. doi: 10.1182/blood-2015-01-622704 26024876 10.1182/blood-2015-01-622704

[17] Erup Larsen M, Kloverpris H, Stryhn A, Koofhethile CK, Sims S, Ndung’u T, et al HLArestrictor—a tool for patient-specific predictions of HLA restriction elements and optimal epitopes within peptides. Immunogenetics. 2011;63: 43–55. doi: 10.1007/s00251-010-0493-5 21079948 10.1007/s00251-010-0493-5

[18] Tamura K, Stecher G, Peterson D, Filipski A, Kumar S. MEGA6: Molecular evolutionary genetics analysis version 6.0. Mol Biol Evol. Oxford University Press; 2013;30: 2725–2729. doi: 10.1093/molbev/mst197 24132122 10.1093/molbev/mst197 PMC3840312

[19] Sievers F, Wilm A, Dineen D, Gibson TJ, Karplus K, Li W, et al Fast, scalable generation of high-quality protein multiple sequence alignments using Clustal Omega. Mol Syst Biol. 2014;7: 539–539. doi: 10.1038/msb.2011.75 21988835 10.1038/msb.2011.75 PMC3261699

[20] Delport W, Poon AFY, Frost SDW, Kosakovsky Pond SL. Datamonkey 2010: A suite of phylogenetic analysis tools for evolutionary biology. Bioinformatics. 2010;26: 2455–2457. doi: 10.1093/bioinformatics/btq429 20671151 10.1093/bioinformatics/btq429 PMC2944195

[21] Krzywinski M, Schein J, Birol I, Connors J, Gascoyne R, Horsman D, et al Circos: An information aesthetic for comparative genomics. Genome Res. 2009;19: 1639–1645. doi: 10.1101/gr.092759.109 19541911 10.1101/gr.092759.109 PMC2752132

[22] Bulek AM, Cole DK, Skowera A, Dolton G, Gras S, Madura F, et al Structural basis for the killing of human beta cells by CD8+ T cells in type 1 diabetes. Nat Immunol. Nature Publishing Group; 2012;13: 283–289. doi: 10.1038/ni.2206 22245737 10.1038/ni.2206 PMC3378510

[23] Van Lummel M, Duinkerken G, Van Veelen PA, De Ru A, Cordfunke R, Zaldumbide A, et al Posttranslational modification of HLA-DQ binding islet autoantigens in type 1 diabetes. Diabetes. 2014;63: 237–247. doi: 10.2337/db12-1214 24089515 10.2337/db12-1214

[24] Skowera A, Ellis RJ, Varela-Calviño R, Arif S, Huang GC, Van-Krinks C, et al CTLs are targeted to kill β cells in patients with type 1 diabetes through recognition of a glucose-regulated preproinsulin epitope. J Clin Invest. 2008;118: 3390–3402. doi: 10.1172/JCI35449 18802479 10.1172/JCI35449 PMC2542849

[25] Herold KC, Brooks-Worrell B, Palmer J, Dosch HM, Peakman M, Gottlieb P, et al Validity and reproducibility of measurement of islet autoreactivity by T-cell assays in subjects with early type 1 diabetes. Diabetes. 2009;58: 2588–2595. doi: 10.2337/db09-0249 19675135 10.2337/db09-0249 PMC2768166

[26] Arif S, Tree TI, Astill TP, Tremble JM, Bishop AJ, Dayan CM, et al Autoreactive T cell responses show proinflammatory polarization in diabetes but a regulatory phenotype in health. J Clin Invest. 2004;113: 451–463. doi: 10.1172/JCI19585 14755342 10.1172/JCI19585 PMC324541

[27] Arif S, Leete P, Nguyen V, Marks K, Nor NM, Estorninho M, et al Blood and islet phenotypes indicate immunological heterogeneity in type 1 diabetes. Diabetes. 2014;63: 3835–3845. doi: 10.2337/db14-0365 24939426 10.2337/db14-0365 PMC4207393

[28] Ali MA, Liu YF, Arif S, Tatovic D, Shariff H, Gibson VB, et al Metabolic and immune effects of immunotherapy with proinsulin peptide in human new-onset type 1 diabetes. Sci Transl Med. 2017;9: eaaf7779 doi: 10.1126/scitranslmed.aaf7779 28794283 10.1126/scitranslmed.aaf7779

[29] Arif S, Gibson VB, Nguyen V, Bingley PJ, Todd JA, Guy C, et al β-cell specific T-lymphocyte response has a distinct inflammatory phenotype in children with Type 1 diabetes compared with adults. Diabet Med. 2017;34: 419–425. doi: 10.1111/dme.13153 27151105 10.1111/dme.13153

[30] Skarsvik S, Puranen J, Honkanen J, Roivainen M, Ilonen J, Holmberg H, et al Coxsackie Virus B4 in Children With Type 1 Diabetes. Gene Expr. 2006;55: 996–1003. 10.2337/diabetes.55.04.06.db05-0630 16567521

[31] Kanno T, Kim K, Kono K, Drescher KM, Chapman NM, Tracy S. Group B Coxsackievirus Diabetogenic Phenotype Correlates with Replication Efficiency. J Virol. 2006;80: 5637–5643. doi: 10.1128/JVI.02361-05 16699045 10.1128/JVI.02361-05 PMC1472143

[32] Rammensee H-G, Bachmann J, Emmerich NPN, Bachor OA, Stevanović S. SYFPEITHI: database for MHC ligands and peptide motifs. Immunogenetics. Springer-Verlag; 1999;50: 213–219. doi: 10.1007/s002510050595 10.1007/s002510050595 10602881

[33] Krogvold L, Edwin B, Buanes T, Frisk G, Skog O, Anagandula M, et al Detection of a low-grade enteroviral infection in the islets of langerhans of living patients newly diagnosed with type 1 diabetes. Diabetes. 2015;64: 1682–1687. doi: 10.2337/db14-1370 25422108 10.2337/db14-1370

[34] Laitinen OH, Honkanen H, Pakkanen O, Oikarinen S, Hankaniemi MM, Huhtala H, et al Coxsackievirus B1 Is Associated With Induction of b -Cell Autoimmunity That Portends Type 1 Diabetes. 2014;63: 446–455. doi: 10.2337/db13-0619 23974921 10.2337/db13-0619

[35] Moya-Suri V, Schlosser M, Zimmermann K, Rjasanowski I, Gürtler L, Mentel R. Enterovirus RNA sequences in sera of schoolchildren in the general population and their association with type 1-diabetes-associated autoantibodies. J Med Microbiol. 2005;54: 879–83. doi: 10.1099/jmm.0.46015-0 16091441 10.1099/jmm.0.46015-0

[36] Andréoletti L, Hober D, Hober-Vandenberghe C, Belaich S, Vantyghem MC, Lefebvre J, et al Detection of coxsackie B virus RNA sequences in whole blood samples from adult patients at the onset of type I diabetes mellitus. J Med Virol. 1997;52: 121–7. 9179756 10.1002/(sici)1096-9071(199706)52:2<121::aid-jmv1>3.0.co;2-5

[37] Nurminen N, Oikarinen S, Hyöty H. Virus infections as potential targets of preventive treatments for type 1 diabetes. Rev Diabet Stud. 2012;9: 260–271. doi: 10.1900/RDS.2012.9.260 23804265 10.1900/RDS.2012.9.260 PMC3740695

[38] McKinney RE, Katz SL, Wilfert CM. Chronic Enteroviral Meningoencephalitis in Agammaglobulinemic Patients. Rev Infect Dis. 1987;9: 334–356. doi: 10.1093/clinids/9.2.334 3296100 10.1093/clinids/9.2.334

[39] Wilfert CM, Buckley RH, Mohanakumar T, Griffith JF, Katz SL, Whisnant JK, et al Persistent and Fatal Central-Nervous-System ECHOvirus Infections in Patients with Agammaglobulinemia. N Engl J Med. Massachusetts Medical Society; 1977;296: 1485–1489. doi: 10.1056/NEJM197706302962601 301244 10.1056/NEJM197706302962601

[40] Fehniger TA, Shah MH, Turner MJ, Vandeusen JB, Whitman SP, Cooper MA, et al Identification of Three HLA-A*0201-Restricted Cytotoxic T Cell Epitopes in the Cytomegalovirus Protein pp65 That Are Conserved Between Eight Strains of the Virus. J Immunol. 1999; 5512–5518. 10553078

[41] Redchenko I V, Rickinson a B. Accessing Epstein-Barr virus-specific T-cell memory with peptide-loaded dendritic cells. J Virol. 1999;73: 334–342. 9847337 10.1128/jvi.73.1.334-342.1999 PMC103838

[42] Grant E, Wu C, Chan K-F, Eckle S, Bharadwaj M, Zou QM, et al Nucleoprotein of influenza A virus is a major target of immunodominant CD8+ T-cell responses. Immunol Cell Biol. Australasian Society for Immunology Inc.; 2013;91: 184–194. 10.1038/icb.2012.78 23399741

[43] Kemball CC, Harkins S, Whitmire JK, Flynn CT, Feuer R, Whitton JL. Coxsackievirus B3 inhibits antigen presentation in vivo, exerting a profound and selective effect on the MHC class I pathway. PLoS Pathog. 2009;5 doi: 10.1371/journal.ppat.1000618 19834548 10.1371/journal.ppat.1000618 PMC2757675

[44] Kemball CC, Flynn CT, Hosking MP, Botten J, Whitton JL. Wild-type coxsackievirus infection dramatically alters the abundance, heterogeneity, and immunostimulatory capacity of conventional dendritic cells in vivo. Virology. Elsevier; 2012;429: 74–90. doi: 10.1016/j.virol.2012.04.005 22551767 10.1016/j.virol.2012.04.005 PMC3358485

