# Supplementary figures and images for: In silico and ex vivo approaches indicate immune pressure on capsid and non-capsid regions of coxsackie B viruses in the human system
Source: PLoS One. 2018 Jun 20;13(6):e0199323. doi: 10.1371/journal.pone.0199323 (PMC6010236; doi:10.1371/journal.pone.0199323)

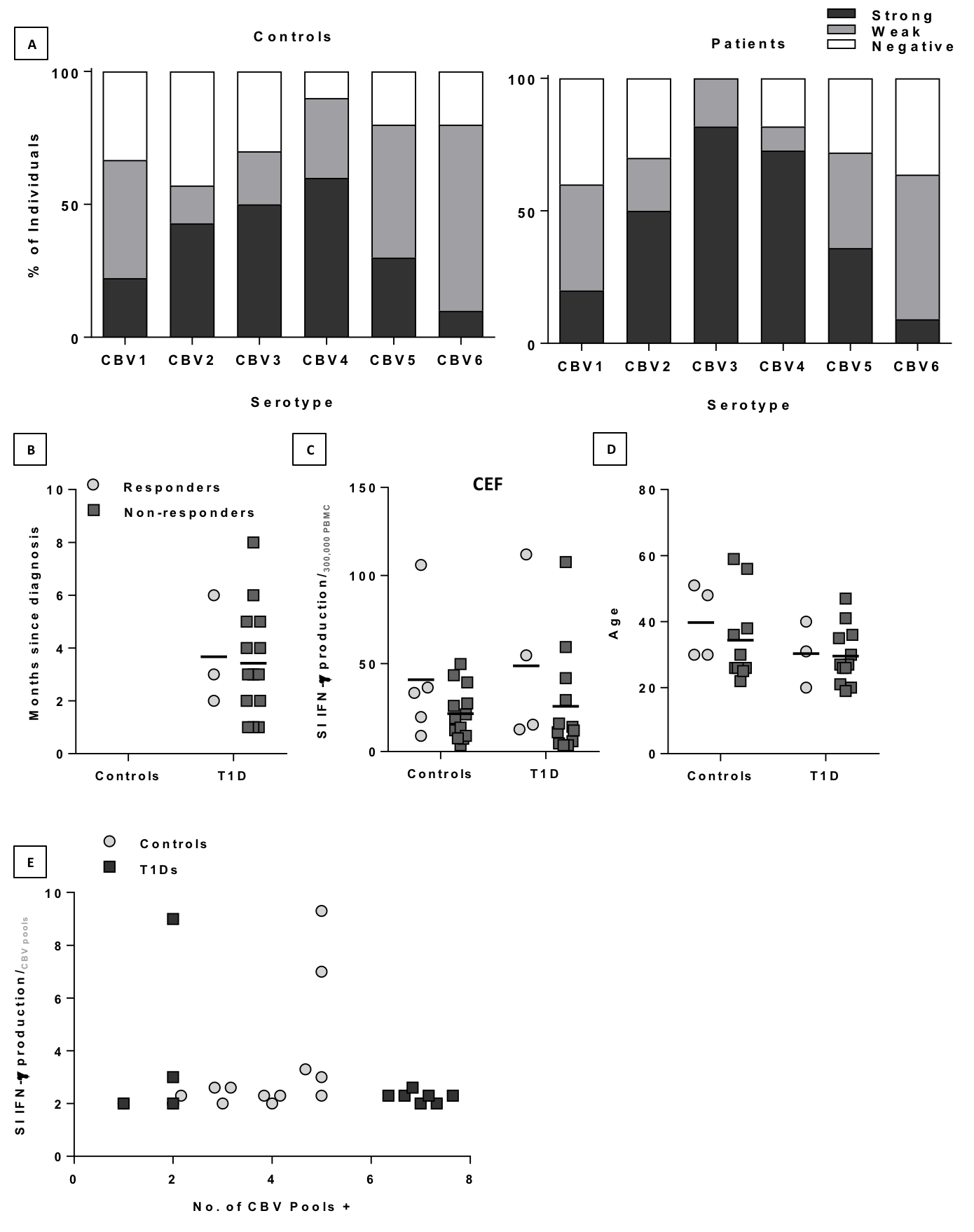

Supplement: S1 Fig — (A) For individuals where samples were available, serum neutralisation of plaque formation by coxsackievirus serotypes 1–6 was assayed as an indicator of serum neutralising antibodies, and so previous virus exposure. Weak neutralisation was defined as a greater than 50% reduction in the number of plaques formed using a 1 in 4 dilution of serum, whilst strong neutralisation was defined by a greater than 50% reduction in the number of plaques using 1 in 16 dilution of serum. The percentage of samples with neutralising capacity for each serotype is presented for recently diagnosed T1Ds (n = 10 for CBV1 and CBV2, n = 11 for CBV3- 6) and non-diabetic controls (n = 9 for CBV1, n = 7 for CBV2 and n = 10 for CBV3-6). Months since diagnosis (B); SI per 3.3 x 10^5^ PBMC for positive control CEF (C) and age (D) are plotted for recently-diagnosed T1Ds against non-diabetic controls and CBV-responders and non-responders. (E) Mean SIs for individuals responding to any CBV peptide pool are plotted against the number of positive hits for that individual. Dark grey points indicate ND-T1Ds and light grey points indicate non-diabetic controls. (TIF) [file pone.0199323.s001.tif]
