# Supplementary material for: In silico and ex vivo approaches indicate immune pressure on capsid and non-capsid regions of coxsackie B viruses in the human system
Source: PLoS One. 2018 Jun 20;13(6):e0199323. doi: 10.1371/journal.pone.0199323 (PMC6010236; doi:10.1371/journal.pone.0199323)
Supplement: S1 Table — Sequences returned from Genbank for the queries “CBV”, “CVB” “Coxsackievirus B” and “Coxsackie B Virus” were collated for use in in silico epitope prediction approaches. (DOCX) [file pone.0199323.s002.docx]

S1 Table: Sequences used for Serotype Specific and Pan-Serotype Epitope Prediction.

| Nucleotide Accession | Protein Accession | Serotype | Authors | Publication | Journal | Year |
| --- | --- | --- | --- | --- | --- | --- |
| AY186745.1 | AAO84298.1 | CBV1 | Tam,P.E., Weber-Sanders,M.L. and Messner,R.P. | Multiple viral determinants mediate myopathogenicity in coxsackievirus B1-induced chronic inflammatory myopathy | Journal of Virology | 2003 |
| AY186746.1 | AAO84299.1 | CBV1 | Tam,P.E., Weber-Sanders,M.L. and Messner,R.P. | Multiple viral determinants mediate myopathogenicity in coxsackievirus B1-induced chronic inflammatory myopathy | Journal of Virology | 2003 |
| AY186747.1 | AAO84300.1 | CBV1 | Tam,P.E., Weber-Sanders,M.L. and Messner,R.P. | Multiple viral determinants mediate myopathogenicity in coxsackievirus B1-induced chronic inflammatory myopathy | Journal of Virology | 2003 |
| AY186748.1 | AAO84301.1 | CBV1 | Tam,P.E., Weber-Sanders,M.L. and Messner,R.P. | Multiple viral determinants mediate myopathogenicity in coxsackievirus B1-induced chronic inflammatory myopathy | Journal of Virology | 2003 |
| EU147493.1 | ABV64405.1 | CBV1 | Cifuente,J.O., Hafenstein,S.L., Song,W.-C., Romanowski,V. and Gomez,R.M. | Molecular determinants of pancreatic and cardiac disease in Coxsackievirus B1 infection | Direct Genbank Submission | 2007 |
| JN596588.1 | AFO64932.1 | CBV1 | Ma,S., Pan,Y. and Li,H. | Complete Nucleotide Sequence of a Human Coxsackievirus B1 MSH/KM9/2009 Strain Isolated in China | Direct Genbank Submission | 2011 |
| JN797615.1 | AET37232.1 | CBV1 | Bachtler,M., Frey,B.M., Frey,F.J., Gorgievski,M., Simonetti,G. and Pasch,A. | Role of enteroviruses in mesangial renal disease | Direct Genbank Submission | 2011 |
| JX976769.1 | AGC54436.1 | CBV1 | Zhang,T., Du,J., Xue,Y., Su,H., Yang,F. and Jin,Q. | Epidemics and Frequent Recombination within Species in Outbreaks of Human Enterovirus B-Associated Hand, Foot and Mouth Disease in Shandong China in 2010 and 2011 | PLosOne | 2013 |
| M16560.1 | NP_040958 | CBV1 | Iizuka,N., Kuge,S. and Nomoto,A. | Complete nucleotide sequence of the genome of coxsackievirus B1 | Virology | 1987 |
| AF081485.1 | AAD46138.1 | CBV2 | Zell,R., Birch-Hirschfeld,E., Fortmuller,U., Henke,A. and Stelzner,A. | Nucleotide Sequence of Coxsackievirus B2 Ohio | Direct Genbank Submission | 1998 |
| AF085363.1 | AAD19874.1 | CBV2 | Polacek,C., Lundgren,A., Andersson,A. and Lindberg,A.M. | Genomic and phylogenetic characterization of coxsackievirus B2 prototype strain Ohio-1 | Virus Research | 1999 |
| EF174468.1 | ABM53473.1 | CBV2 | Hong,J., Kang,B., Kim,A., Kim,J., Lee,S., Cheon,D. and Jee,Y. | Human Coxsackievirus B2 | Direct Genbank Submission | 2006 |
| EF174469.1 | ABM53474.1 | CBV2 | Hong,J., Kang,B., Kim,A., Kim,J., Lee,S., Cheon,D. and Jee,Y. | Human Coxsackievirus B2 | Direct Genbank Submission | 2006 |
| AF231763.1 | AAG23918.1 | CBV3 | Schmidtke,M., Selinka,H.C., Heim,A., Jahn,B., Tonew,M., Kandolf,R., Stelzner,A. and Zell,R. | Attachment of coxsackievirus B3 variants to various cell lines: mapping of phenotypic differences to capsid protein VP1 | Virology | 2000 |
| AF231764.1 | AAG23919.1 | CBV3 | Schmidtke,M., Selinka,H.C., Heim,A., Jahn,B., Tonew,M., Kandolf,R., Stelzner,A. and Zell,R. | Attachment of coxsackievirus B3 variants to various cell lines: mapping of phenotypic differences to capsid protein VP1 | Virology | 2000 |
| AF231765.1 | AAG23920.1 | CBV3 | Schmidtke,M., Selinka,H.C., Heim,A., Jahn,B., Tonew,M., Kandolf,R., Stelzner,A. and Zell,R. | Attachment of coxsackievirus B3 variants to various cell lines: mapping of phenotypic differences to capsid protein VP1 | Virology | 2000 |
| AY673831.1 | AAT79531.1 | CBV3 | Lee,C.K., Kono,K., Haas,E., Kim,K.S., Drescher,K.M., Chapman,N.M. and Tracy,S. | Characterization of an infectious cDNA copy of the genome of a naturally occurring, avirulent coxsackievirus B3 clinical isolate | Journal of General Virology | 2005 |
| AY752944.1 | AAV34211.1 | CBV3 | Chapman,N.M., Tu,Z., Tracy,S. and Gauntt,C.J. | An infectious cDNA copy of the genome of a non-cardiovirulent coxsackievirus B3 strain: its complete sequence analysis and comparison to the genomes of cardiovirulent coxsackieviruses | Archives of Virology | 1994 |
| AY752945.1 | AAV34212.1 | CBV3 | Chapman,N.M., Tu,Z., Tracy,S. and Gauntt,C.J. | An infectious cDNA copy of the genome of a non-cardiovirulent coxsackievirus B3 strain: its complete sequence analysis and comparison to the genomes of cardiovirulent coxsackieviruses | Archives of Virology | 1994 |
| AY752946.1 | AAV34213.1 | CBV3 | Tracy,S., Chapman,N.M. and Tu,Z. | Coxsackievirus B3 from an infectious cDNA copy of the genome is cardiovirulent in mice | Archives of Virology | 1992 |
| EU144042.1 | ABW34429.1 | CBV3 | Krogstad,P.A., Hammon,R.J., Halnon,N.J. and Whitton,L. | Fatal Neonatal Infection with a Recombinant Human Enterovirus | Direct Genbank Submission | 2007 |
| FJ000001.1 | ACH91034.1 | CBV3 | Liu,J., Ma,S., Wang,L., Liu,L. and Li,Q. | CVB3 isolates in Fuyang, China from 2008 | Direct Genbank Submission | 2008 |
| FJ357838.1 | ACJ05389.1 | CBV3 | Tian,X., Zhou,R., Gong,S. and Zhu,B. | Coxsackievirus B3 isolated in Guangzhou, China in 2008 | Direct Genbank Submission | 2008 |
| GQ141875.1 | ACS34758.1 | CBV3 | Du,J., Wu,Z., Xue,Y., Zhang,T., Yang,F. and Jin,Q. | Complete genome sequence of a human coxsackievirus b3 from a child with myocarditis in beijing, china | Genome Announcements | 2013 |
| GU109481.1 | ACY40750.1 | CBV3 | He,W., Lu,H., Song,D., Zhao,K., Gai,X., Wang,X., Chen,Q. and Gao,F. | The evidence of Coxsackievirus B3 induced myocarditis as the cause of death in a Sichuan snub-nosed monkey (Rhinopithecus roxellana) | Journal of Medical Primatology | 2009 |
| JN048468.1 | AEH42467.1 | CBV3 | Pan,J., Narayanan,B., Shah,S., Yoder,J.D., Cifuente,J.O., Hafenstein,S. and Bergelson,J.M. | Single amino Acid changes in the virus capsid permit coxsackievirus b3 to bind decay-accelerating factor | Journal of Virology | 2011 |
| JN048469.1 | AEH42468.1 | CBV3 | Pan,J., Narayanan,B., Shah,S., Yoder,J.D., Cifuente,J.O., Hafenstein,S. and Bergelson,J.M. | Single amino Acid changes in the virus capsid permit coxsackievirus b3 to bind decay-accelerating factor | Journal of Virology | 2011 |
| JN979570.1 | AFC88096.1 | CBV3 | Nielsen,S.C., Mourier,T., Baandrup,U., Soland,T.M., Bertelsen,M.F., Gilbert,M.T. and Nielsen,L.P. | Probable transmission of Coxsackie b3 virus from human to chimpanzee, Denmark | Emerging Infectious Disease | 2012 |
| JQ040513.1 | AFD33642.1 | CBV3 | Wang,L., Dong,C., Chen,D.E. and Song,Z. | Coxsackievirus-induced acute neonatal central nervous system disease model | International Journal of Clinical Experimental Pathology | 2014 |
| JX312064.1 | AFS18536.1 | CBV3 | Gangaplara,A., Massilamany,C., Vu,H., Pattnaik,A.K. and Reddy,J. | NA | Direct Genbank Submission | 2012 |
| JX843810.1 | AFY09604.1 | CBV3 | Ma,S. | NA | Direct Genbank Submission | 2012 |
| JX976770.1 | AGC54437.1 | CBV3 | Zhang,T., Du,J., Xue,Y., Su,H., Yang,F. and Jin,Q. | Epidemics and Frequent Recombination within Species in Outbreaks of Human Enterovirus B-Associated Hand, Foot and Mouth Disease in Shandong China in 2010 and 2011 | PLosOne | 2013 |
| KC481610.1 | AGJ72750.1 | CBV3 | Zhang,X., Zheng,Z., Shu,B., Liu,X., Zhang,Z., Liu,Y., Bai,B., Hu,Q., Mao,P. and Wang,H. | Human astrocytic cells support persistent coxsackievirus B3 infection | Journal of Virology | 2013 |
| KJ020100.1 | AHM88285.1 | CBV3 | Liu,J. and Ma,S. | NA | Direct Genbank Submission | 2014 |
| KJ025083.1 | AHK09954.1 | CBV3 | Liu,B., Li,Z., Xiang,F., Li,F., Zheng,Y. and Wang,G. | The whole genome sequence of Coxsackievirus B3 MKP strain leading to myocarditis and its molecular phylogenetic analysis | Virology Journal | 2014 |
| KJ489414.1 | AHY19027.1 | CBV3 | Aubry,F., Nougairede,A., de Fabritus,L., Querat,G., Gould,E.A. and de Lamballerie,X. | Single-stranded positive-sense RNA viruses generated in days using infectious subgenomic amplicons | Journal of General Virology | 2014 |
| M16572.1 | AAA74400.1 | CBV3 | Lindberg,A.M., Stalhandske,P.O. and Pettersson,U. | Genome of coxsackievirus B3 | Virology | 1987 |
| M33854.1 | AAA42931.1 | CBV3 | Klump,W.M., Bergmann,I., Muller,B.C., Ameis,D. and Kandolf,R. | Complete nucleotide sequence of infectious Coxsackievirus B3 cDNA: two initial 5' uridine residues are regained during plus-strand RNA synthesis | Journal of Virology | 1990 |
| M88483.1 | AAB59927.1 | CBV3 | Lindberg,A.M., Stalhandske,P.O. and Pettersson,U. | Genome of coxsackievirus B3 | Virology | 1987 |
| U57056.1 | AAB02228.1 | CBV3 | Knowlton,K.U., Jeon,E.S., Berkley,N., Wessely,R. and Huber,S. | A mutation in the puff region of VP2 attenuates the myocarditic phenotype of an infectious cDNA of the Woodruff variant of coxsackievirus B3 | Journal of Virology | 1996 |
| AF311939.1 | AAL37156.1 | CBV4 | Lindberg,A.M. and Andersson,P. | Molecular analysis of a coxsackievirus B4 strain E2 variant | Direct Genbank Submission | 2000 |
| DQ480420.1 | ABF19105.1 | CBV4 | Dotta,F., Censini,S., van Halteren,A.G., Marselli,L., Masini,M., Dionisi,S., Mosca,F., Boggi,U., Muda,A.O., Prato,S.D., Elliott,J.F., Covacci,A., Rappuoli,R., Roep,B.O. and Marchetti,P. | Coxsackie B4 virus infection of beta cells and natural killer cell insulitis in recent-onset type 1 diabetic patients | PNAS | 2007 |
| JX308222.1 | AFR79234.1 | CBV4 | Hu,Y.F., Du,J., Zhao,R., Xue,Y., Yang,F. and Jin,Q. | Complete genome sequence of a recombinant coxsackievirus b4 from a patient with a fatal case of hand, foot, and mouth disease in Guangxi, China | Journal of Virology | 2012 |
| KC558559.1 | AHB37357.1 | CBV4 | Nielsen,S.C.A., Gilbert,T. and Nielsen,L.P. | Deep sequencing of fifteen coxsackie B4 virus genomes from Danish patients and their phylogenetic relationship to the diabetogenic coxsackie B4 strain, E2 | Direct Genbank Submission | 2013 |
| KC558560.1 | AHB37358.1 | CBV4 | Nielsen,S.C.A., Gilbert,T. and Nielsen,L.P. | Deep sequencing of fifteen coxsackie B4 virus genomes from Danish patients and their phylogenetic relationship to the diabetogenic coxsackie B4 strain, E2 | Direct Genbank Submission | 2013 |
| KC558561.1 | AHB37359.1 | CBV4 | Nielsen,S.C.A., Gilbert,T. and Nielsen,L.P. | Deep sequencing of fifteen coxsackie B4 virus genomes from Danish patients and their phylogenetic relationship to the diabetogenic coxsackie B4 strain, E2 | Direct Genbank Submission | 2013 |
| KC558562.1 | AHB37360.1 | CBV4 | Nielsen,S.C.A., Gilbert,T. and Nielsen,L.P. | Deep sequencing of fifteen coxsackie B4 virus genomes from Danish patients and their phylogenetic relationship to the diabetogenic coxsackie B4 strain, E2 | Direct Genbank Submission | 2013 |
| KC558563.1 | AHB37361.1 | CBV4 | Nielsen,S.C.A., Gilbert,T. and Nielsen,L.P. | Deep sequencing of fifteen coxsackie B4 virus genomes from Danish patients and their phylogenetic relationship to the diabetogenic coxsackie B4 strain, E2 | Direct Genbank Submission | 2013 |
| KC558564.1 | AHB37362.1 | CBV4 | Nielsen,S.C.A., Gilbert,T. and Nielsen,L.P. | Deep sequencing of fifteen coxsackie B4 virus genomes from Danish patients and their phylogenetic relationship to the diabetogenic coxsackie B4 strain, E2 | Direct Genbank Submission | 2013 |
| KC558565.1 | AHB37363.1 | CBV4 | Nielsen,S.C.A., Gilbert,T. and Nielsen,L.P. | Deep sequencing of fifteen coxsackie B4 virus genomes from Danish patients and their phylogenetic relationship to the diabetogenic coxsackie B4 strain, E2 | Direct Genbank Submission | 2013 |
| KC558566.1 | AHB37364.1 | CBV4 | Nielsen,S.C.A., Gilbert,T. and Nielsen,L.P. | Deep sequencing of fifteen coxsackie B4 virus genomes from Danish patients and their phylogenetic relationship to the diabetogenic coxsackie B4 strain, E2 | Direct Genbank Submission | 2013 |
| KC558567.1 | AHB37365.1 | CBV4 | Nielsen,S.C.A., Gilbert,T. and Nielsen,L.P. | Deep sequencing of fifteen coxsackie B4 virus genomes from Danish patients and their phylogenetic relationship to the diabetogenic coxsackie B4 strain, E2 | Direct Genbank Submission | 2013 |
| KC558568.1 | AHB37366.1 | CBV4 | Nielsen,S.C.A., Gilbert,T. and Nielsen,L.P. | Deep sequencing of fifteen coxsackie B4 virus genomes from Danish patients and their phylogenetic relationship to the diabetogenic coxsackie B4 strain, E2 | Direct Genbank Submission | 2013 |
| KC558569.1 | AHB37367.1 | CBV4 | Nielsen,S.C.A., Gilbert,T. and Nielsen,L.P. | Deep sequencing of fifteen coxsackie B4 virus genomes from Danish patients and their phylogenetic relationship to the diabetogenic coxsackie B4 strain, E2 | Direct Genbank Submission | 2013 |
| KC558570.1 | AHB37368.1 | CBV4 | Nielsen,S.C.A., Gilbert,T. and Nielsen,L.P. | Deep sequencing of fifteen coxsackie B4 virus genomes from Danish patients and their phylogenetic relationship to the diabetogenic coxsackie B4 strain, E2 | Direct Genbank Submission | 2013 |
| KC558571.1 | AHB37369.1 | CBV4 | Nielsen,S.C.A., Gilbert,T. and Nielsen,L.P. | Deep sequencing of fifteen coxsackie B4 virus genomes from Danish patients and their phylogenetic relationship to the diabetogenic coxsackie B4 strain, E2 | Direct Genbank Submission | 2013 |
| KC558572.1 | AHB37370.1 | CBV4 | Nielsen,S.C.A., Gilbert,T. and Nielsen,L.P. | Deep sequencing of fifteen coxsackie B4 virus genomes from Danish patients and their phylogenetic relationship to the diabetogenic coxsackie B4 strain, E2 | Direct Genbank Submission | 2013 |
| KC558573.1 | AHB37371.1 | CBV4 | Nielsen,S.C.A., Gilbert,T. and Nielsen,L.P. | Deep sequencing of fifteen coxsackie B4 virus genomes from Danish patients and their phylogenetic relationship to the diabetogenic coxsackie B4 strain, E2 | Direct Genbank Submission | 2013 |
| KF781524.1 | AHL77760.1 | CBV4 | Tian,X., Zhang,Y., Gu,S., Fan,Y., Sun,Q., Zhang,B., Yan,S., Xu,W., Ma,X. and Wang,W. | New coxsackievirus B4 genotype circulating in Inner Mongolia Autonomous Region, China | PLosOne | 2014 |
| KF781525.1 | AHL77761.1 | CBV4 | Tian,X., Zhang,Y., Gu,S., Fan,Y., Sun,Q., Zhang,B., Yan,S., Xu,W., Ma,X. and Wang,W. | New coxsackievirus B4 genotype circulating in Inner Mongolia Autonomous Region, China | PLosOne | 2014 |
| KF878966.1 | AHJ40480.1 | CBV4 | Madden,K., Wang,C.Y.T., Arden,K.E., Sloots,T.P. and Mackay,I.M. | The molecular characterization of a Coxsackievirus B4 variant isolated from a child with upper respiratory tract infection | Direct Genbank Submission | 2013 |
| S76772.1 | AAB33885.1 | CBV4 | Kang,Y., Chatterjee,N.K., Nodwell,M.J. and Yoon,J.W. | Complete nucleotide sequence of a strain of coxsackie B4 virus of human origin that induces diabetes in mice and its comparison with nondiabetogenic coxsackie B4 JBV strain | Journal of Medical Virology | 1994 |
| X05690.1 | CAA29172.1 | CBV4 | Jenkins,O., Booth,J.D., Minor,P.D. and Almond,J.W. | The complete nucleotide sequence of coxsackievirus B4 and its comparison to other members of the Picornaviridae | Journal of General Virology | 1987 |
| JX276378.1 | AFR67087.1 | CBV5 | Hu,Y.F., Zhao,R., Xue,Y., Yang,F. and Jin,Q. | Full genome sequence of a novel coxsackievirus B5 strain isolated from neurological hand, foot, and mouth disease patients in china | Journal of Virology | 2012 |
| AF114383.1 | AAF21971.1 | CBV5 | Lindberg,A.M. and Polacek,C. | Molecular analysis of the prototype coxsackievirus B5 genome | Archives of Virology | 2000 |
| AY875692.1 | AAW71476.1 | CBV5 | Yi,J. and Kim,E.-C. | NA | Direct Genbank Submission | 2005 |
| GU376747.1 | ADL27740.1 | CBV5 | Shan,T. | NA | Direct Genbank Submission | 2009 |
| JN580070.1 | AEX07779.1 | CBV5 | Han,J.F., Jiang,T., Fan,X.L., Yang,L.M., Yu,M., Cao,R.Y., Wang,J.Z., Qin,E.D. and Qin,C.F. | Recombination of human coxsackievirus b5 in hand, foot, and mouth disease patients, china | Emerging Infectious Disease | 2012 |
| JN695050.1 | AFA28143.1 | CBV5 | Han,J.F., Jiang,T., Fan,X.L., Yang,L.M., Yu,M., Cao,R.Y., Wang,J.Z., Qin,E.D. and Qin,C.F. | Recombination of human coxsackievirus b5 in hand, foot, and mouth disease patients, china | Emerging Infectious Disease | 2012 |
| JN695051.1 | AFA28144.1 | CBV5 | Han,J.F., Jiang,T., Fan,X.L., Yang,L.M., Yu,M., Cao,R.Y., Wang,J.Z., Qin,E.D. and Qin,C.F. | Recombination of human coxsackievirus b5 in hand, foot, and mouth disease patients, china | Emerging Infectious Disease | 2012 |
| JX017380.1 | AFO42817.1 | CBV5 | Ma,H., Huang,X., Kang,K., Li,X., Tang,X., Ren,Y., Wang,Y., Zhao,G. and Xu,B. | Recombination in human coxsackievirus B5 strains that caused an outbreak of viral encephalitis in Henan, China | Archives of Virology | 2013 |
| JX017381.1 | AFO42818.1 | CBV5 | Ma,H., Huang,X., Kang,K., Li,X., Tang,X., Ren,Y., Wang,Y., Zhao,G. and Xu,B. | Recombination in human coxsackievirus B5 strains that caused an outbreak of viral encephalitis in Henan, China | Archives of Virology | 2013 |
| JX017383.1 | AFO42820.1 | CBV5 | Ma,H. | Molecular biological identification of pathogens which caused an outbreak of viral encephalitis in Henan area | Direct Genbank Submission | 2012 |
| JX843811.1 | AFY09605.1 | CBV5 | Ma,H. | Molecular biological identification of pathogens which caused an outbreak of viral encephalitis in Henan area | Direct Genbank Submission | 2012 |
| AF039205.1 | AAD02132.1 | CBV6 | Zell,R.H. | Cloning and sequencing of an infectious cDNA of Coxsackievirus B6 (CVB6) | Direct Genbank Submission | 1997 |
| AF105342.1 | AAF12719.1 | CBV6 | Martino,T.A., Tellier,R., Petric,M., Irwin,D.M., Afshar,A. and Liu,P.P. | The complete consensus sequence of coxsackievirus B6 and generation of infectious clones by long RT-PCR | Virus Research | 1999 |
| AF114384.1 | AAF21972.1 | CBV6 | Lindberg,A.M., Polacek,C., Johansson,S., Lundgren,A., Andersson,A. and Van Ranst,M. | Strategy for sequence analysis of complete enterovirus genomes. The prototype strain of coxsackievirus B6 | Direct Genbank Submission | 1998 |
| JQ041368.1 | AFD32988.1 | CBV6 | Demina,A.V., Ternovoi,V.A., Svyatchenko,V.A., Tikunova,N.V., Tikunov,A.I., Kiselev,N., Loktev,V.B., Netesov,S.V. and Chumakov,P.M. | NA | Direct Genbank Submission | 2011 |
