# Supplementary material for: In silico and ex vivo approaches indicate immune pressure on capsid and non-capsid regions of coxsackie B viruses in the human system
Source: PLoS One. 2018 Jun 20;13(6):e0199323. doi: 10.1371/journal.pone.0199323 (PMC6010236; doi:10.1371/journal.pone.0199323)
Supplement: S2 Table — Cryopreserved PBMCs were thawed and rested for two days at high density (1.5 x10^7^/ml) in X-VIVO media + 5% human AB serum (Sigma). Cultured PBMCs were then recounted and plated at 3.3x10^6^/ml and 1x10^6^ cells stimulated with indicated peptides at 5μg/ml each final concentration, alongside diluent alone and viral peptide mix CEF (Mabtech) control conditions for three hours. Samples were transferred in triplicate to pre-coated and blocked IFNγ ELISpot plates (U-Cytech) and incubated for 24 hours to capture cytokine released. Cytokine release was identified as per the manufacturer’s instructions and plates counted using the Bio-sys Bioreader. The mean IFNy SI per 3.3x10^5^ cells of three replicate wells and total spots per 106 are presented for ELISpot assays against serotype specific epitope at position 538–548. Results from the neutralisation of CBV plaque formation by serum assays are also presented; strong responses were taken as those sera reducing plaque formation by 50% with a 1 in 16 dilution of serum, and weak responses were taken as those sera that reduced plaque formation by 50% with a 1 in 4 dilution of serum when compared to virus only controls. (DOCX) [file pone.0199323.s003.docx]

**S2 Table: Serotype Specific ELISpot Responses and Serum Plaque Formation Neutralisation Assay results**

|  |  | ELISpot | | | | | | | | Serum Neutralising Antibody Assay | | | | | |
| --- | --- | --- | --- | --- | --- | --- | --- | --- | --- | --- | --- | --- | --- | --- | --- |
|  |  | DMSO | CB1 | CB2 | CB3 | CB4 | CB5 | CB6 | CEF | CB1 | CB2 | CB3 | CB4 | CB5 | CB6 |
| **Ctrl 1** | Mean SI | 1.0 | 0.8 | 0.5 | 0.8 | 0.4 | 0.8 | 0.6 | 12.1 | Neg | W | S | S | W | W |
|  | Total Spots | 28.0 | 21.0 | 14.0 | 23.0 | 12.0 | 22.0 | 17.0 | 339.0 |  |  |  |  |  |  |
| **Ctrl 2** | Mean SI | 0.0 | 0.0 | 0.0 | 0.0 | 0.3 | 0.0 | 0.3 | 9.0 | - | - | - | - | - | - |
|  | Total Spots | 0.0 | 0.0 | 0.0 | 0.0 | 1.0 | 0.0 | 1.0 | 27.0 |  |  |  |  |  |  |
| **Ctrl 3** | Mean SI | 0.0 | 0.3 | 0.7 | 0.7 | 0.3 | 1.0 | 0.3 | 3.7 | W | W | W | W | Neg | Neg |
|  | Total Spots | 0.0 | 1.0 | 2.0 | 2.0 | 1.0 | 3.0 | 1.0 | 11.0 |  |  |  |  |  |  |
| **Ctrl 4** | Mean SI | 0.0 | - | - | - | - | - | - | 39.3 | W | S | S | S | S | W |
|  | Total Spots | 0.0 | - | - | - | - | - | - | 118.0 |  |  |  |  |  |  |
| **Ctrl 5** | Mean SI | 0.0 | 0.3 | 0.3 | 0.7 | 0.0 | 0.3 | 0.7 | 19.0 | S | S | S | Neg | W | W |
|  | Total Spots | 0.0 | 1.0 | 1.0 | 2.0 | 0.0 | 1.0 | 2.0 | 57.0 |  |  |  |  |  |  |
| **Ctrl 6** | Mean SI | 1.0 | 0.2 | 0.7 | 0.5 | 0.4 | 0.9 | 0.8 | 13.7 | Neg | Neg | Neg | W | W | Neg |
|  | Total Spots | 10.0 | 2.0 | 7.0 | 5.0 | 4.0 | 9.0 | 8.0 | 137.0 |  |  |  |  |  |  |
| **Ctrl 7** | Mean SI | 0.0 | 0.3 | 0.7 | 0.7 | 0.3 | 0.3 | 0.7 | 7.3 | Neg | W | W | W | S | Neg |
|  | Total Spots | 0.0 | 1.0 | 2.0 | 2.0 | 1.0 | 1.0 | 2.0 | 22.0 |  |  |  |  |  |  |
| **Ctrl 8** | Mean SI | 0.3 | 3.3 | 0.0 | 0.0 | 0.0 | 0.0 | 0.0 | 26.0 | S | - | S | S | S | S |
|  | Total Spots | 1.0 | 10.0 | 0.0 | 0.0 | 0.0 | 0.0 | 0.0 | 78.0 |  |  |  |  |  |  |
| **Ctrl 9** | Mean SI | 0.0 | 0.0 | 0.3 | 0.0 | 0.3 | 0.0 | 0.3 | 49.7 | - | - | - | - | - | - |
|  | Total Spots | 0.0 | 0.0 | 1.0 | 0.0 | 1.0 | 0.0 | 1.0 | 149.0 |  |  |  |  |  |  |
| **Ctrl 10** | Mean SI | 1.0 | 1.6 | 1.0 | 1.5 | 1.0 | 0.6 | 1.2 | 7.5 | - | - | - | - | - | - |
|  | Total Spots | 10.0 | 17.0 | 11.0 | 16.0 | 11.0 | 6.0 | 13.0 | 79.0 |  |  |  |  |  |  |
| **Ctrl 11** | Mean SI | 1.0 | - | - | - | - | - | - | 19.7 | - | - | - | - | - | - |
|  | Total Spots | 6.0 | - | - | - | - | - | - | 118.0 |  |  |  |  |  |  |
| **Ctrl 12** | Mean SI | 0.7 | 1.0 | 2.0 | 1.0 | 0.7 | 1.3 | 1.3 | 106.0 | W | S | W | S | S | Neg |
|  | Total Spots | 2.0 | 3.0 | 6.0 | 3.0 | 2.0 | 4.0 | 4.0 | 318.0 |  |  |  |  |  |  |
| **Ctrl 13** | Mean SI | 0.0 | 1.0 | 1.0 | 2.3 | 0.7 | 0.7 | 1.0 | 9.0 | W | S | W | S | W | W |
|  | Total Spots | 0.0 | 3.0 | 3.0 | 7.0 | 2.0 | 2.0 | 3.0 | 27.0 |  |  |  |  |  |  |
| **Ctrl 14** | Mean SI | 0.3 | 7.0 | 1.0 | 0.7 | 3.3 | 1.7 | 0.3 | 36.3 | - | - | - | - | - | - |
|  | Total Spots | 1.0 | 21.0 | 3.0 | 2.0 | 10.0 | 5.0 | 1.0 | 109.0 |  |  |  |  |  |  |
| **Ctrl 15** | Mean SI | 0.3 | 0.0 | 0.0 | 0.3 | 0.0 | 0.0 | 0.3 | 27.3 | - | - | - | - | - | - |
|  | Total Spots | 1.0 | 0.0 | 0.0 | 1.0 | 0.0 | 0.0 | 1.0 | 82.0 |  |  |  |  |  |  |
| **Ctrl 16** | Mean SI | 1.0 | 0.7 | 1.0 | 0.0 | 0.0 | 0.0 | 1.3 | 21.1 | - | - | - | - | - | - |
|  | Total Spots | 7.0 | 5.0 | 7.0 | 0.0 | 0.0 | 0.0 | 9.0 | 148.0 |  |  |  |  |  |  |
| **Ctrl 17** | Mean SI | 1.0 | - | - | - | - | - | - | 33.3 | - | - | - | - | - | - |
|  | Total Spots | 3.0 | - | - | - | - | - | - | 100.0 |  |  |  |  |  |  |
| **Ctrl 18** | Mean SI | 0.7 | - | - | - | - | - | - | 43.3 | - | - | - | - | - | - |
|  | Total Spots | 2.0 | - | - | - | - | - | - | 130.0 |  |  |  |  |  |  |
| **T1D 1** | Mean SI | 0.0 | 0.3 | 0.0 | 0.0 | 0.3 | 0.0 | 0.0 | 14.0 | W | W | S | S | Neg | Neg |
|  | Total Spots | 0.0 | 1.0 | 0.0 | 0.0 | 1.0 | 0.0 | 0.0 | 42.0 |  |  |  |  |  |  |
| **T1D 2** | Mean SI | 0.0 | 0.0 | 0.0 | 0.0 | 0.0 | 0.3 | 0.0 | 11.0 | W | S | W | S | W | Neg |
|  | Total Spots | 0.0 | 0.0 | 0.0 | 0.0 | 0.0 | 1.0 | 0.0 | 33.0 |  |  |  |  |  |  |
| **T1D 3** | Mean SI | 1.0 | 2.3 | 1.3 | 1.0 | 2.0 | 1.3 | 2.3 | 54.7 | S | W | W | S | W | W |
|  | Total Spots | 3.0 | 7.0 | 4.0 | 3.0 | 6.0 | 4.0 | 7.0 | 164.0 |  |  |  |  |  |  |
| **T1D 4** | Mean SI | 0.0 | 0.0 | 0.7 | 0.3 | 0.0 | 0.3 | 0.3 | 41.7 | W | Neg | S | Neg | Neg | W |
|  | Total Spots | 0.0 | 0.0 | 2.0 | 1.0 | 0.0 | 1.0 | 1.0 | 125.0 |  |  |  |  |  |  |
| **T1D 5** | Mean SI | 1.0 | 1.8 | 1.1 | 1.1 | 0.9 | 0.6 | 0.8 | 5.9 | W | W | W | S | S | Neg |
|  | Total Spots | 44.0 | 77.0 | 50.0 | 48.0 | 38.0 | 26.0 | 35.0 | 258.0 |  |  |  |  |  |  |
| **T1D6** | Mean SI | 0.3 | 0.0 | 0.0 | 0.3 | 0.0 | 0.3 | 0.7 | 3.7 | S | S | S | S | W | S |
|  | Total Spots | 1.0 | 0.0 | 0.0 | 1.0 | 0.0 | 1.0 | 2.0 | 11.0 |  |  |  |  |  |  |
| **T1D7** | Mean SI | 0.0 | - | - | - | - | - | - | 16.0 | W | - | S | S | S | Neg |
|  | Total Spots | 0.0 | - | - | - | - | - | - | 48.0 |  |  |  |  |  |  |
| **T1D8** | Mean SI | 0.3 | - | - | - | - | - | - | 3.7 | - | - | - | - | - | - |
|  | Total Spots | 1.0 | - | - | - | - | - | - | 11.0 |  |  |  |  |  |  |
| **T1D9** | Mean SI | 1.0 | 0.2 | 0.1 | 0.8 | 0.5 | 0.9 | 1.0 | 12.0 | Neg | S | S | S | S | W |
|  | Total Spots | 10.0 | 2.0 | 1.0 | 8.0 | 5.0 | 9.0 | 10.0 | 120.0 |  |  |  |  |  |  |
| **T1D 10** | Mean SI | 0.0 | 0.0 | 0.0 | 0.0 | 0.0 | 0.0 | 0.3 | 107.7 | W | S | W | S | Neg | W |
|  | Total Spots | 0.0 | 0.0 | 0.0 | 0.0 | 0.0 | 0.0 | 1.0 | 323.0 |  |  |  |  |  |  |
| **T1D 11** | Mean SI | 0.0 | 0.0 | 0.0 | 1.0 | 0.0 | 0.0 | 0.3 | 59.3 | Neg | S | S | S | W | Neg |
|  | Total Spots | 0.0 | 0.0 | 0.0 | 3.0 | 0.0 | 0.0 | 1.0 | 178.0 |  |  |  |  |  |  |
| **T1D 12** | Mean SI | 0.3 | - | - | - | - | - | - | 4.7 | W | W | S | S | W | Neg |
|  | Total Spots | 1.0 | - | - | - | - | - | - | 14.0 |  |  |  |  |  |  |
| **T1D 13** | Mean SI | 1.0 | 0.3 | 1.0 | 0.7 | 0.3 | 1.7 | 0.0 | 112.0 | W | S | S | S | S | W |
|  | Total Spots | 3.0 | 1.0 | 3.0 | 2.0 | 1.0 | 5.0 | 0.0 | 336.0 |  |  |  |  |  |  |
| **T1D 14** | Mean SI | 0.3 | 0.7 | 0.3 | 0.7 | 1.3 | 0.3 | 0.7 | 12.7 | - | - | - | - | - | - |
|  | Total Spots | 1.0 | 2.0 | 1.0 | 2.0 | 4.0 | 1.0 | 2.0 | 38.0 |  |  |  |  |  |  |
| **T1D 15** | Mean SI | 1.0 | - | - | - | - | - | - | 15.3 | S | S | S | S | W | W |
|  | Total Spots | 3.0 | - | - | - | - | - | - | 46.0 |  |  |  |  |  |  |
| **T1D 16** | Mean SI | 0.3 | - | - | - | - | - | - | 29.3 | S | Neg | S | S | S | S |
|  | Total Spots | 1.0 | - | - | - | - | - | - | 88.0 |  |  |  |  |  |  |
