# Supplementary material for: In silico and ex vivo approaches indicate immune pressure on capsid and non-capsid regions of coxsackie B viruses in the human system
Source: PLoS One. 2018 Jun 20;13(6):e0199323. doi: 10.1371/journal.pone.0199323 (PMC6010236; doi:10.1371/journal.pone.0199323)
Supplement: S3 Table — Sequences returned from Genbank for the queries “CBV1”, “CVB1”, “Coxsackievirus B1” and “Coxsackie B Virus 1” were collated for use in in silico epitope prediction approaches. (DOCX) [file pone.0199323.s004.docx]

Table S2: Sequences Used for CBV1 MEME.

| Genbank Accession Number | Publication Title | Authours | Journal | Year |
| --- | --- | --- | --- | --- |
| AB162733 | Sequence of enterovirus | Munemura,T., Saikusa,M. and Kawakami,C. | Direct Genbank Submission | 2004 |
| AB162734 | Sequence of enterovirus | Munemura,T., Saikusa,M. and Kawakami,C. | Direct Genbank Submission | 2004 |
| AB162735 | Sequence of enterovirus | Munemura,T., Saikusa,M. and Kawakami,C. | Direct Genbank Submission | 2004 |
| AB162736 | Sequence of enterovirus | Munemura,T., Saikusa,M. and Kawakami,C. | Direct Genbank Submission | 2004 |
| AB162737 | Sequence of enterovirus | Munemura,T., Saikusa,M. and Kawakami,C. | Direct Genbank Submission | 2004 |
| AB167989 | Molecular evolution of enteroviruses in Fukuoka City | Wakatsuki,K., Yamasaki,S., Manabe,K. and Takeda,A. | Direct Genbank Submission | 2004 |
| AB268125 | Molecular typing and epidemiology of non-polio enteroviruses isolated from Yunnan Province, the People's Republic of China | Bingjun,T., Yoshida,H., Yan,W., Lin,L., Tsuji,T., Shimizu,H. and Miyamura,T. | Journal of Medical Virology | 2008 |
| AB268126 | Molecular typing and epidemiology of non-polio enteroviruses isolated from Yunnan Province, the People's Republic of China | Bingjun,T., Yoshida,H., Yan,W., Lin,L., Tsuji,T., Shimizu,H. and Miyamura,T. | Journal of Medical Virology | 2008 |
| AB268127 | Molecular typing and epidemiology of non-polio enteroviruses isolated from Yunnan Province, the People's Republic of China | Bingjun,T., Yoshida,H., Yan,W., Lin,L., Tsuji,T., Shimizu,H. and Miyamura,T. | Journal of Medical Virology | 2008 |
| AB598062 | Investigation of enteroviruses from healthy children in Zhoutong, Yunnan Province, 2010 | Tang,J., Tian,B., Lu,L., Ding,Z. and Zhang,J. | Direct Genbank Submission | 2010 |
| AB598063 | Investigation of enteroviruses from healthy children in Zhoutong, Yunnan Province, 2010 | Tang,J., Tian,B., Lu,L., Ding,Z. and Zhang,J. | Direct Genbank Submission | 2010 |
| AB598064 | Investigation of enteroviruses from healthy children in Zhoutong, Yunnan Province, 2010 | Tang,J., Tian,B., Lu,L., Ding,Z. and Zhang,J. | Direct Genbank Submission | 2010 |
| AB598065 | Investigation of enteroviruses from healthy children in Zhoutong, Yunnan Province, 2010 | Tang,J., Tian,B., Lu,L., Ding,Z. and Zhang,J. | Direct Genbank Submission | 2010 |
| AB598066 | Investigation of enteroviruses from healthy children in Zhoutong, Yunnan Province, 2010 | Tang,J., Tian,B., Lu,L., Ding,Z. and Zhang,J. | Direct Genbank Submission | 2010 |
| AB601195 | Enterovirus | Yoshitomi,H., Sera,N., Ishibashi,T. and Tagami,S. | Direct Genbank Submission | 2010 |
| AB601196 | Enterovirus | Yoshitomi,H., Sera,N., Ishibashi,T. and Tagami,S. | Direct Genbank Submission | 2010 |
| AB601197 | Enterovirus | Yoshitomi,H., Sera,N., Ishibashi,T. and Tagami,S. | Direct Genbank Submission | 2010 |
| AB601198 | Enterovirus | Yoshitomi,H., Sera,N., Ishibashi,T. and Tagami,S. | Direct Genbank Submission | 2010 |
| AB601199 | Enterovirus | Yoshitomi,H., Sera,N., Ishibashi,T. and Tagami,S. | Direct Genbank Submission | 2010 |
| AB759899 | Molecular typing of human enteroviruses from healthy children in Yunnan province, 2012(part 1) | Chen,S., Tang,J. and Zhang,J. | Direct Genbank Submission | 2012 |
| AB759900 | Molecular typing of human enteroviruses from healthy children in Yunnan province, 2012(part 1) | Chen,S., Tang,J. and Zhang,J. | Direct Genbank Submission | 2012 |
| AB759901 | Molecular typing of human enteroviruses from healthy children in Yunnan province, 2012(part 1) | Chen,S., Tang,J. and Zhang,J. | Direct Genbank Submission | 2012 |
| AB759902 | Molecular typing of human enteroviruses from healthy children in Yunnan province, 2012(part 1) | Chen,S., Tang,J. and Zhang,J. | Direct Genbank Submission | 2012 |
| AB781729 | Molecular typing of human enteroviruses from healthy children in Yunnan province, 2012 (part 2) | Tian,B., Chen,S. and Zhang,J. | Direct Genbank Submission | 2013 |
| AB902819 | Detection of enteroviruses from 2010 to 2012 in Okinawa, Japan | Nidaira,M., Taira,K., Kyan,H., Takara,T., Okano,S. and Kudaka,J. | Direct Genbank Submission | 2014 |
| AB902820 | Detection of enteroviruses from 2010 to 2012 in Okinawa, Japan | Nidaira,M., Taira,K., Kyan,H., Takara,T., Okano,S. and Kudaka,J. | Direct Genbank Submission | 2014 |
| AB902821 | Detection of enteroviruses from 2010 to 2012 in Okinawa, Japan | Nidaira,M., Taira,K., Kyan,H., Takara,T., Okano,S. and Kudaka,J. | Direct Genbank Submission | 2014 |
| AF252169 | Molecular characterization of human enteroviruses in clinical samples: comparison between VP2, VP1, and RNA polymerase regions using RT nested PCR assays and direct sequencing of products. | Casas,I., Palacios,G.F., Trallero,G., Cisterna,D., Freire,M.C. and Tenorio,A. | Journal of Medical Virology | 2001 |
| AF252170 | Molecular characterization of human enteroviruses in clinical samples: comparison between VP2, VP1, and RNA polymerase regions using RT nested PCR assays and direct sequencing of products. | Casas,I., Palacios,G.F., Trallero,G., Cisterna,D., Freire,M.C. and Tenorio,A. | Journal of Medical Virology | 2001 |
| AJ279158 | Molecular strategy for ‘serotyping’ of human enteroviruses | Caro,V., Guillot,S., Delpeyroux,F. and Crainic,R. | Journal of General Virology | 2001 |
| AJ279186 | Molecular strategy for ‘serotyping’ of human enteroviruses | Caro,V., Guillot,S., Delpeyroux,F. and Crainic,R. | Journal of General Virology | 2001 |
| AJ279187 | Molecular strategy for ‘serotyping’ of human enteroviruses | Caro,V., Guillot,S., Delpeyroux,F. and Crainic,R. | Journal of General Virology | 2001 |
| AJ309258 | Molecular Identification and Typing of Enteroviruses Isolated from Clinical Specimens | Manzara,S., Muscillo,M., La Rosa,G., Marianelli,C., Cattani,P. and Fadda,G. | Journal of Clinical Microbiology | 2002 |
| AM492463 | Prospective identification of enteroviruses involved in meningitis in 2006 through direct genotyping in cerebrospinal fluid | Mirand,A., Henquell,C., Archimbaud,C., Chambon,M., Charbonne,F., Peigue-Lafeuille,H. and Bailly,J.L. | Journal of Clinical Microbiology | 2008 |
| AM711043 | Prospective identification of enteroviruses involved in meningitis in 2006 through direct genotyping in cerebrospinal fluid | Mirand,A., Henquell,C., Archimbaud,C., Chambon,M., Charbonne,F., Peigue-Lafeuille,H. and Bailly,J.L. | Journal of Clinical Microbiology | 2008 |
| AM711081 | Prospective identification of enteroviruses involved in meningitis in 2006 through direct genotyping in cerebrospinal fluid | Mirand,A., Henquell,C., Archimbaud,C., Chambon,M., Charbonne,F., Peigue-Lafeuille,H. and Bailly,J.L. | Journal of Clinical Microbiology | 2008 |
| AY373091 | RNA recombination plays a major role in genomic change during circulation of coxsackie B viruses | Oberste,M.S., Penaranda,S. and Pallansch,M.A | Journal of Virology | 2004 |
| AY373092 | RNA recombination plays a major role in genomic change during circulation of coxsackie B viruses | Oberste,M.S., Penaranda,S. and Pallansch,M.A | Journal of Virology | 2004 |
| AY373093 | RNA recombination plays a major role in genomic change during circulation of coxsackie B viruses | Oberste,M.S., Penaranda,S. and Pallansch,M.A | Journal of Virology | 2004 |
| AY373094 | RNA recombination plays a major role in genomic change during circulation of coxsackie B viruses | Oberste,M.S., Penaranda,S. and Pallansch,M.A | Journal of Virology | 2004 |
| AY373095 | RNA recombination plays a major role in genomic change during circulation of coxsackie B viruses | Oberste,M.S., Penaranda,S. and Pallansch,M.A | Journal of Virology | 2004 |
| AY373096 | RNA recombination plays a major role in genomic change during circulation of coxsackie B viruses | Oberste,M.S., Penaranda,S. and Pallansch,M.A | Journal of Virology | 2004 |
| AY373097 | RNA recombination plays a major role in genomic change during circulation of coxsackie B viruses | Oberste,M.S., Penaranda,S. and Pallansch,M.A | Journal of Virology | 2004 |
| AY373098 | RNA recombination plays a major role in genomic change during circulation of coxsackie B viruses | Oberste,M.S., Penaranda,S. and Pallansch,M.A | Journal of Virology | 2004 |
| AY373099 | RNA recombination plays a major role in genomic change during circulation of coxsackie B viruses | Oberste,M.S., Penaranda,S. and Pallansch,M.A | Journal of Virology | 2004 |
| AY373100 | RNA recombination plays a major role in genomic change during circulation of coxsackie B viruses | Oberste,M.S., Penaranda,S. and Pallansch,M.A | Journal of Virology | 2004 |
| AY634233 | Concentration, detection and identification of enteroviruses in sewage | Papaventsis,D.C., Siafakas,N. and Markoylatos,P. | Direct Genbank Submission | 2004 |
| AY634259 | Concentration, detection and identification of enteroviruses in sewage | Papaventsis,D.C., Siafakas,N. and Markoylatos,P. | Direct Genbank Submission | 2004 |
| AY634261 | Concentration, detection and identification of enteroviruses in sewage | Papaventsis,D.C., Siafakas,N. and Markoylatos,P. | Direct Genbank Submission | 2004 |
| AY634267 | Concentration, detection and identification of enteroviruses in sewage | Papaventsis,D.C., Siafakas,N. and Markoylatos,P. | Direct Genbank Submission | 2004 |
| AY634268 | Concentration, detection and identification of enteroviruses in sewage | Papaventsis,D.C., Siafakas,N. and Markoylatos,P. | Direct Genbank Submission | 2004 |
| AY634270 | Concentration, detection and identification of enteroviruses in sewage | Papaventsis,D.C., Siafakas,N. and Markoylatos,P. | Direct Genbank Submission | 2004 |
| AY679738 | Nucleotide analysis and phylogenetic study of the homology boundaries of coxsackie A and B viruses | Bolanaki,E., Kottaridi,C., Markoulatos,P., Margaritis,L. and Katsorchis,T. | Virus Genes | 2005 |
| AY679759 | Nucleotide analysis and phylogenetic study of the homology boundaries of coxsackie A and B viruses | Bolanaki,E., Kottaridi,C., Markoulatos,P., Margaritis,L. and Katsorchis,T. | Virus Genes | 2005 |
| AY694115 | Echovirus 30 analysis in Korea 2003 | Jeong,E.J., Park,H.S., Lee,J.H., Jee,Y.M. and Jeong,H.K | Direct Genbank Submission | 2003 |
| AY701748 | Coxsackievirus B1 analysis in Korea 2003 | Jeong,E.J., Park,H.S., Lee,J.H., Jee,Y.M. and Jeong,H.K. | Direct Genbank Submission | 2003 |
| AY831483 | Nucleotide analysis and phylogenetic study of the homology boundaries of coxsackie A and B viruses | Bolanaki,E., Kottaridi,C., Markoulatos,P., Margaritis,L. and Katsorchis,T. | Virus Genes | 2005 |
| AY831484 | Nucleotide analysis and phylogenetic study of the homology boundaries of coxsackie A and B viruses | Bolanaki,E., Kottaridi,C., Markoulatos,P., Margaritis,L. and Katsorchis,T. | Virus Genes | 2005 |
| AY831486 | Nucleotide analysis and phylogenetic study of the homology boundaries of coxsackie A and B viruses | Bolanaki,E., Kottaridi,C., Markoulatos,P., Margaritis,L. and Katsorchis,T. | Virus Genes | 2005 |
| AY831497 | Nucleotide analysis and phylogenetic study of the homology boundaries of coxsackie A and B viruses | Bolanaki,E., Kottaridi,C., Markoulatos,P., Margaritis,L. and Katsorchis,T. | Virus Genes | 2005 |
| AY831500 | Nucleotide analysis and phylogenetic study of the homology boundaries of coxsackie A and B viruses | Bolanaki,E., Kottaridi,C., Markoulatos,P., Margaritis,L. and Katsorchis,T. | Virus Genes | 2005 |
| AY831501 | Nucleotide analysis and phylogenetic study of the homology boundaries of coxsackie A and B viruses | Bolanaki,E., Kottaridi,C., Markoulatos,P., Margaritis,L. and Katsorchis,T. | Virus Genes | 2005 |
| AY831502 | Nucleotide analysis and phylogenetic study of the homology boundaries of coxsackie A and B viruses | Bolanaki,E., Kottaridi,C., Markoulatos,P., Margaritis,L. and Katsorchis,T. | Virus Genes | 2005 |
| AY831503 | Nucleotide analysis and phylogenetic study of the homology boundaries of coxsackie A and B viruses | Bolanaki,E., Kottaridi,C., Markoulatos,P., Margaritis,L. and Katsorchis,T. | Virus Genes | 2005 |
| AY831504 | Nucleotide analysis and phylogenetic study of the homology boundaries of coxsackie A and B viruses | Bolanaki,E., Kottaridi,C., Markoulatos,P., Margaritis,L. and Katsorchis,T. | Virus Genes | 2005 |
| AY831505 | Nucleotide analysis and phylogenetic study of the homology boundaries of coxsackie A and B viruses | Bolanaki,E., Kottaridi,C., Markoulatos,P., Margaritis,L. and Katsorchis,T. | Virus Genes | 2005 |
| AY831506 | Nucleotide analysis and phylogenetic study of the homology boundaries of coxsackie A and B viruses | Bolanaki,E., Kottaridi,C., Markoulatos,P., Margaritis,L. and Katsorchis,T. | Virus Genes | 2005 |
| DQ251350 | Frequency and dynamics of recombination within different species of human enteroviruses | Simmonds,P. and Welch,J | Journal of Virology | 2006 |
| DQ317166 | High prevalence of human enterovirus a infections in natural circulation of human enteroviruses | Witso,E., Palacios,G., Cinek,O., Stene,L.C., Grinde,B., Janowitz,D., Lipkin,W.I. and Ronningen,K.S. | Journal of Clinical Microbiology | 2006 |
| DQ317167 | High prevalence of human enterovirus a infections in natural circulation of human enteroviruses | Witso,E., Palacios,G., Cinek,O., Stene,L.C., Grinde,B., Janowitz,D., Lipkin,W.I. and Ronningen,K.S. | Journal of Clinical Microbiology | 2006 |
| DQ317168 | High prevalence of human enterovirus a infections in natural circulation of human enteroviruses | Witso,E., Palacios,G., Cinek,O., Stene,L.C., Grinde,B., Janowitz,D., Lipkin,W.I. and Ronningen,K.S. | Journal of Clinical Microbiology | 2006 |
| DQ317169 | High prevalence of human enterovirus a infections in natural circulation of human enteroviruses | Witso,E., Palacios,G., Cinek,O., Stene,L.C., Grinde,B., Janowitz,D., Lipkin,W.I. and Ronningen,K.S. | Journal of Clinical Microbiology | 2006 |
| DQ317170 | High prevalence of human enterovirus a infections in natural circulation of human enteroviruses | Witso,E., Palacios,G., Cinek,O., Stene,L.C., Grinde,B., Janowitz,D., Lipkin,W.I. and Ronningen,K.S. | Journal of Clinical Microbiology | 2006 |
| DQ530391 | Molecular characterization of enteroviruses isolated from patients with aseptic meningitis in Korea, 2005 | Lee,S.T., Ki,C.S. and Lee,N.Y. | Archives of Virology | 2007 |
| DQ530392 | Molecular characterization of enteroviruses isolated from patients with aseptic meningitis in Korea, 2005 | Lee,S.T., Ki,C.S. and Lee,N.Y. | Archives of Virology | 2007 |
| DQ530393 | Molecular characterization of enteroviruses isolated from patients with aseptic meningitis in Korea, 2005 | Lee,S.T., Ki,C.S. and Lee,N.Y. | Archives of Virology | 2007 |
| DQ530394 | Molecular characterization of enteroviruses isolated from patients with aseptic meningitis in Korea, 2005 | Lee,S.T., Ki,C.S. and Lee,N.Y. | Archives of Virology | 2007 |
| FJ525916 | Direct identification of human enterovirus serotypes in cerebrospinal fluid by amplification of the VP1 region | Leitch,E.C., Harvala,H., Robertson,I., Ubillos,I., Templeton,K. and Simmonds,P. | Journal of Clinical Virology | 2009 |
| FJ525920 | Direct identification of human enterovirus serotypes in cerebrospinal fluid by amplification of the VP1 region | Leitch,E.C., Harvala,H., Robertson,I., Ubillos,I., Templeton,K. and Simmonds,P. | Journal of Clinical Virology | 2009 |
| FJ868283 | Identification of 20 common human enterovirus serotypes by use of a reverse transcription-PCR-based reverse line blot hybridization assay. | Zhou,F., Kong,F., McPhie,K., Ratnamohan,M., Donovan,L., Zeng,F., Gilbert,G.L. and Dwyer,D.E. | Journal of Clinical Microbiology | 2009 |
| FJ868284 | Identification of 20 common human enterovirus serotypes by use of a reverse transcription-PCR-based reverse line blot hybridization assay. | Zhou,F., Kong,F., McPhie,K., Ratnamohan,M., Donovan,L., Zeng,F., Gilbert,G.L. and Dwyer,D.E. | Journal of Clinical Microbiology | 2009 |
| FJ868324 | NA | Zhou,F., Kong,F. and Dwyer,D.E. | Direct Genbank Submission | 2009 |
| FJ868325 | NA | Zhou,F., Kong,F. and Dwyer,D.E. | Direct Genbank Submission | 2009 |
| FJ868326 | NA | Zhou,F., Kong,F. and Dwyer,D.E. | Direct Genbank Submission | 2009 |
| FR797992 | Enterovirus Co-infections and Onychomadesis after Hand, Foot, and Mouth Disease, Spain 2008 | Bracho,M.A., Gonzalez-Candelas,F., Valero,A., Cordoba,J. and Salazar, A. | Emerging Infectious Disease | 2011 |
| FR797993 | Enterovirus Co-infections and Onychomadesis after Hand, Foot, and Mouth Disease, Spain 2008 | Bracho,M.A., Gonzalez-Candelas,F., Valero,A., Cordoba,J. and Salazar, A. | Emerging Infectious Disease | 2011 |
| FR797994 | Enterovirus Co-infections and Onychomadesis after Hand, Foot, and Mouth Disease, Spain 2008 | Bracho,M.A., Gonzalez-Candelas,F., Valero,A., Cordoba,J. and Salazar, A. | Emerging Infectious Disease | 2011 |
| FR797995 | Enterovirus Co-infections and Onychomadesis after Hand, Foot, and Mouth Disease, Spain 2008 | Bracho,M.A., Gonzalez-Candelas,F., Valero,A., Cordoba,J. and Salazar, A. | Emerging Infectious Disease | 2011 |
| FR797996 | Enterovirus Co-infections and Onychomadesis after Hand, Foot, and Mouth Disease, Spain 2008 | Bracho,M.A., Gonzalez-Candelas,F., Valero,A., Cordoba,J. and Salazar, A. | Emerging Infectious Disease | 2011 |
| FR797997 | Enterovirus Co-infections and Onychomadesis after Hand, Foot, and Mouth Disease, Spain 2008 | Bracho,M.A., Gonzalez-Candelas,F., Valero,A., Cordoba,J. and Salazar, A. | Emerging Infectious Disease | 2011 |
| FR797998 | Enterovirus Co-infections and Onychomadesis after Hand, Foot, and Mouth Disease, Spain 2008 | Bracho,M.A., Gonzalez-Candelas,F., Valero,A., Cordoba,J. and Salazar, A. | Emerging Infectious Disease | 2011 |
| FR797999 | Enterovirus Co-infections and Onychomadesis after Hand, Foot, and Mouth Disease, Spain 2008 | Bracho,M.A., Gonzalez-Candelas,F., Valero,A., Cordoba,J. and Salazar, A. | Emerging Infectious Disease | 2011 |
| FR798000 | Enterovirus Co-infections and Onychomadesis after Hand, Foot, and Mouth Disease, Spain 2008 | Bracho,M.A., Gonzalez-Candelas,F., Valero,A., Cordoba,J. and Salazar, A. | Emerging Infectious Disease | 2011 |
| GQ329732 | Molecular epidemiology of species B enteroviruses isolated from shadong Province of China | Wang,H., Li,Y., Tao,Z., Fan,Q., Yang,H. and Xu,A. | Direct Genbank Submission | 2009 |
| GQ329733 | Molecular epidemiology of species B enteroviruses isolated from shadong Province of China | Wang,H., Li,Y., Tao,Z., Fan,Q., Yang,H. and Xu,A. | Direct Genbank Submission | 2009 |
| GQ329734 | Molecular epidemiology of species B enteroviruses isolated from shadong Province of China | Wang,H., Li,Y., Tao,Z., Fan,Q., Yang,H. and Xu,A. | Direct Genbank Submission | 2009 |
| GQ329735 | Molecular epidemiology of species B enteroviruses isolated from shadong Province of China | Wang,H., Li,Y., Tao,Z., Fan,Q., Yang,H. and Xu,A. | Direct Genbank Submission | 2009 |
| GQ329736 | Molecular epidemiology of species B enteroviruses isolated from shadong Province of China | Wang,H., Li,Y., Tao,Z., Fan,Q., Yang,H. and Xu,A. | Direct Genbank Submission | 2009 |
| GQ329737 | Molecular epidemiology of species B enteroviruses isolated from shadong Province of China | Wang,H., Li,Y., Tao,Z., Fan,Q., Yang,H. and Xu,A. | Direct Genbank Submission | 2009 |
| GU949565 | Molecular characteristic study of Coxsackievirus B1 strains from the samples of aseptic meningitis patients in Zhejiang during 2008-2009 | Yan,J.-Y., Gong,L.-M. and Ge,Q. | Direct Genbank Submission | 2010 |
| GU949566 | Molecular characteristic study of Coxsackievirus B1 strains from the samples of aseptic meningitis patients in Zhejiang during 2008-2009 | Yan,J.-Y., Gong,L.-M. and Ge,Q. | Direct Genbank Submission | 2010 |
| GU949567 | Molecular characteristic study of Coxsackievirus B1 strains from the samples of aseptic meningitis patients in Zhejiang during 2008-2009 | Yan,J.-Y., Gong,L.-M. and Ge,Q. | Direct Genbank Submission | 2010 |
| GU949568 | Molecular characteristic study of Coxsackievirus B1 strains from the samples of aseptic meningitis patients in Zhejiang during 2008-2009 | Yan,J.-Y., Gong,L.-M. and Ge,Q. | Direct Genbank Submission | 2010 |
| HF948082 | Phylogenetic Patterns of Human Coxsackievirus B5 Arise from Population Dynamics between Two Genogroups and Reveal Evolutionary Factors of Molecular Adaptation and Transmission | Henquell,C., Mirand,A., Richter,J., Schuffenecker,I., Bottiger,B., Diedrich,S., Terletskaia-Ladwig,E., Christodoulou,C., Peigue-Lafeuille,H. and Bailly,J.L. | Journal of Virology | 2013 |
| HG793665 | Variations in cerebrospinal fluid viral loads among enterovirus genotypes in patients hospitalized with laboratory-confirmed meningitis due to enterovirus | Volle,R., Bailly,J.L., Mirand,A., Pereira,B., Marque-Juillet,S., Chambon,M., Regagnon,C., Brebion,A., Henquell,C., Peigue-Lafeuille,H. and Archimbaud,C. | Journal of Infectious Disease | 2014 |
| HG793666 | Variations in cerebrospinal fluid viral loads among enterovirus genotypes in patients hospitalized with laboratory-confirmed meningitis due to enterovirus | Volle,R., Bailly,J.L., Mirand,A., Pereira,B., Marque-Juillet,S., Chambon,M., Regagnon,C., Brebion,A., Henquell,C., Peigue-Lafeuille,H. and Archimbaud,C. | Journal of Infectious Disease | 2014 |
| HM584455 | Onychomadesis after a hand, foot, and mouth disease outbreak in Spain, 2009 | Cabrerizo,M., De Miguel,T., Armada,A., Martinez-Risco,R., Pousa,A., and Trallero,G. | Epidemiology and Infection | 2010 |
| HM584456 | Onychomadesis after a hand, foot, and mouth disease outbreak in Spain, 2009 | Cabrerizo,M., De Miguel,T., Armada,A., Martinez-Risco,R., Pousa,A., and Trallero,G. | Epidemiology and Infection | 2010 |
| HM584457 | Onychomadesis after a hand, foot, and mouth disease outbreak in Spain, 2009 | Cabrerizo,M., De Miguel,T., Armada,A., Martinez-Risco,R., Pousa,A., and Trallero,G. | Epidemiology and Infection | 2010 |
| HM584458 | Onychomadesis after a hand, foot, and mouth disease outbreak in Spain, 2009 | Cabrerizo,M., De Miguel,T., Armada,A., Martinez-Risco,R., Pousa,A., and Trallero,G. | Epidemiology and Infection | 2010 |
| HM584459 | Onychomadesis after a hand, foot, and mouth disease outbreak in Spain, 2009 | Cabrerizo,M., De Miguel,T., Armada,A., Martinez-Risco,R., Pousa,A., and Trallero,G. | Epidemiology and Infection | 2010 |
| HM584460 | Onychomadesis after a hand, foot, and mouth disease outbreak in Spain, 2009 | Cabrerizo,M., De Miguel,T., Armada,A., Martinez-Risco,R., Pousa,A., and Trallero,G. | Epidemiology and Infection | 2010 |
| HM584461 | Onychomadesis after a hand, foot, and mouth disease outbreak in Spain, 2009 | Cabrerizo,M., De Miguel,T., Armada,A., Martinez-Risco,R., Pousa,A., and Trallero,G. | Epidemiology and Infection | 2010 |
| HM584462 | Onychomadesis after a hand, foot, and mouth disease outbreak in Spain, 2009 | Cabrerizo,M., De Miguel,T., Armada,A., Martinez-Risco,R., Pousa,A., and Trallero,G. | Epidemiology and Infection | 2010 |
| HM584463 | Onychomadesis after a hand, foot, and mouth disease outbreak in Spain, 2009 | Cabrerizo,M., De Miguel,T., Armada,A., Martinez-Risco,R., Pousa,A., and Trallero,G. | Epidemiology and Infection | 2010 |
| HM584464 | Onychomadesis after a hand, foot, and mouth disease outbreak in Spain, 2009 | Cabrerizo,M., De Miguel,T., Armada,A., Martinez-Risco,R., Pousa,A., and Trallero,G. | Epidemiology and Infection | 2010 |
| HM584465 | Onychomadesis after a hand, foot, and mouth disease outbreak in Spain, 2009 | Cabrerizo,M., De Miguel,T., Armada,A., Martinez-Risco,R., Pousa,A., and Trallero,G. | Epidemiology and Infection | 2010 |
| HM584466 | Onychomadesis after a hand, foot, and mouth disease outbreak in Spain, 2009 | Cabrerizo,M., De Miguel,T., Armada,A., Martinez-Risco,R., Pousa,A., and Trallero,G. | Epidemiology and Infection | 2010 |
| HM584467 | Onychomadesis after a hand, foot, and mouth disease outbreak in Spain, 2009 | Cabrerizo,M., De Miguel,T., Armada,A., Martinez-Risco,R., Pousa,A., and Trallero,G. | Epidemiology and Infection | 2010 |
| HM584468 | Onychomadesis after a hand, foot, and mouth disease outbreak in Spain, 2009 | Cabrerizo,M., De Miguel,T., Armada,A., Martinez-Risco,R., Pousa,A., and Trallero,G. | Epidemiology and Infection | 2010 |
| HM584469 | Onychomadesis after a hand, foot, and mouth disease outbreak in Spain, 2009 | Cabrerizo,M., De Miguel,T., Armada,A., Martinez-Risco,R., Pousa,A., and Trallero,G. | Epidemiology and Infection | 2010 |
| HM584470 | Onychomadesis after a hand, foot, and mouth disease outbreak in Spain, 2009 | Cabrerizo,M., De Miguel,T., Armada,A., Martinez-Risco,R., Pousa,A., and Trallero,G. | Epidemiology and Infection | 2010 |
| HM584471 | Onychomadesis after a hand, foot, and mouth disease outbreak in Spain, 2009 | Cabrerizo,M., De Miguel,T., Armada,A., Martinez-Risco,R., Pousa,A., and Trallero,G. | Epidemiology and Infection | 2010 |
| HM584472 | Onychomadesis after a hand, foot, and mouth disease outbreak in Spain, 2009 | Cabrerizo,M., De Miguel,T., Armada,A., Martinez-Risco,R., Pousa,A., and Trallero,G. | Epidemiology and Infection | 2010 |
| HM584473 | Onychomadesis after a hand, foot, and mouth disease outbreak in Spain, 2009 | Cabrerizo,M., De Miguel,T., Armada,A., Martinez-Risco,R., Pousa,A., and Trallero,G. | Epidemiology and Infection | 2010 |
| HM584474 | Onychomadesis after a hand, foot, and mouth disease outbreak in Spain, 2009 | Cabrerizo,M., De Miguel,T., Armada,A., Martinez-Risco,R., Pousa,A., and Trallero,G. | Epidemiology and Infection | 2010 |
| HM584475 | Onychomadesis after a hand, foot, and mouth disease outbreak in Spain, 2009 | Cabrerizo,M., De Miguel,T., Armada,A., Martinez-Risco,R., Pousa,A., and Trallero,G. | Epidemiology and Infection | 2010 |
| HQ407400 | Study of Coxsackievirus B1 isolated from an outbreak associated with HFMD in Henan province in China | Huang,L., Du,Y.-H. and Li,R.-F. | Direct Genbank Submission | 2010 |
| HQ664088 | Molecular epidemiological study of human enterovirus circulating in India | Kumar,A., Shukla,D., Srivastava,S. and Dhole,T.N. | Direct Genbank Submission | 2010 |
| HQ685858 | Epidemics of enterovirus infection in Chungnam Korea, 2008 and 2009 | Baek,K., Yeo,S., Lee,B., Park,K., Song,J., Yu,J., Rheem,I., Kim,J., Hwang,S., Choi,Y., Cheon,D. and Park,J. | Virology Journal | 2011 |
| HQ685859 | Epidemics of enterovirus infection in Chungnam Korea, 2008 and 2009 | Baek,K., Yeo,S., Lee,B., Park,K., Song,J., Yu,J., Rheem,I., Kim,J., Hwang,S., Choi,Y., Cheon,D. and Park,J. | Virology Journal | 2011 |
| HQ685860 | Epidemics of enterovirus infection in Chungnam Korea, 2008 and 2009 | Baek,K., Yeo,S., Lee,B., Park,K., Song,J., Yu,J., Rheem,I., Kim,J., Hwang,S., Choi,Y., Cheon,D. and Park,J. | Virology Journal | 2011 |
| HQ685861 | Epidemics of enterovirus infection in Chungnam Korea, 2008 and 2009 | Baek,K., Yeo,S., Lee,B., Park,K., Song,J., Yu,J., Rheem,I., Kim,J., Hwang,S., Choi,Y., Cheon,D. and Park,J. | Virology Journal | 2011 |
| HQ685862 | Epidemics of enterovirus infection in Chungnam Korea, 2008 and 2009 | Baek,K., Yeo,S., Lee,B., Park,K., Song,J., Yu,J., Rheem,I., Kim,J., Hwang,S., Choi,Y., Cheon,D. and Park,J. | Virology Journal | 2011 |
| HQ685863 | Epidemics of enterovirus infection in Chungnam Korea, 2008 and 2009 | Baek,K., Yeo,S., Lee,B., Park,K., Song,J., Yu,J., Rheem,I., Kim,J., Hwang,S., Choi,Y., Cheon,D. and Park,J. | Virology Journal | 2011 |
| HQ685864 | Epidemics of enterovirus infection in Chungnam Korea, 2008 and 2009 | Baek,K., Yeo,S., Lee,B., Park,K., Song,J., Yu,J., Rheem,I., Kim,J., Hwang,S., Choi,Y., Cheon,D. and Park,J. | Virology Journal | 2011 |
| HQ685865 | Epidemics of enterovirus infection in Chungnam Korea, 2008 and 2009 | Baek,K., Yeo,S., Lee,B., Park,K., Song,J., Yu,J., Rheem,I., Kim,J., Hwang,S., Choi,Y., Cheon,D. and Park,J. | Virology Journal | 2011 |
| HQ685866 | Epidemics of enterovirus infection in Chungnam Korea, 2008 and 2009 | Baek,K., Yeo,S., Lee,B., Park,K., Song,J., Yu,J., Rheem,I., Kim,J., Hwang,S., Choi,Y., Cheon,D. and Park,J. | Virology Journal | 2011 |
| HQ685867 | Epidemics of enterovirus infection in Chungnam Korea, 2008 and 2009 | Baek,K., Yeo,S., Lee,B., Park,K., Song,J., Yu,J., Rheem,I., Kim,J., Hwang,S., Choi,Y., Cheon,D. and Park,J. | Virology Journal | 2011 |
| HQ685868 | Epidemics of enterovirus infection in Chungnam Korea, 2008 and 2009 | Baek,K., Yeo,S., Lee,B., Park,K., Song,J., Yu,J., Rheem,I., Kim,J., Hwang,S., Choi,Y., Cheon,D. and Park,J. | Virology Journal | 2011 |
| HQ685869 | Epidemics of enterovirus infection in Chungnam Korea, 2008 and 2009 | Baek,K., Yeo,S., Lee,B., Park,K., Song,J., Yu,J., Rheem,I., Kim,J., Hwang,S., Choi,Y., Cheon,D. and Park,J. | Virology Journal | 2011 |
| HQ685870 | Epidemics of enterovirus infection in Chungnam Korea, 2008 and 2009 | Baek,K., Yeo,S., Lee,B., Park,K., Song,J., Yu,J., Rheem,I., Kim,J., Hwang,S., Choi,Y., Cheon,D. and Park,J. | Virology Journal | 2011 |
| HQ685871 | Epidemics of enterovirus infection in Chungnam Korea, 2008 and 2009 | Baek,K., Yeo,S., Lee,B., Park,K., Song,J., Yu,J., Rheem,I., Kim,J., Hwang,S., Choi,Y., Cheon,D. and Park,J. | Virology Journal | 2011 |
| HQ685872 | Epidemics of enterovirus infection in Chungnam Korea, 2008 and 2009 | Baek,K., Yeo,S., Lee,B., Park,K., Song,J., Yu,J., Rheem,I., Kim,J., Hwang,S., Choi,Y., Cheon,D. and Park,J. | Virology Journal | 2011 |
| HQ685873 | Epidemics of enterovirus infection in Chungnam Korea, 2008 and 2009 | Baek,K., Yeo,S., Lee,B., Park,K., Song,J., Yu,J., Rheem,I., Kim,J., Hwang,S., Choi,Y., Cheon,D. and Park,J. | Virology Journal | 2011 |
| HQ685874 | Epidemics of enterovirus infection in Chungnam Korea, 2008 and 2009 | Baek,K., Yeo,S., Lee,B., Park,K., Song,J., Yu,J., Rheem,I., Kim,J., Hwang,S., Choi,Y., Cheon,D. and Park,J. | Virology Journal | 2011 |
| HQ685875 | Epidemics of enterovirus infection in Chungnam Korea, 2008 and 2009 | Baek,K., Yeo,S., Lee,B., Park,K., Song,J., Yu,J., Rheem,I., Kim,J., Hwang,S., Choi,Y., Cheon,D. and Park,J. | Virology Journal | 2011 |
| HQ685876 | Epidemics of enterovirus infection in Chungnam Korea, 2008 and 2009 | Baek,K., Yeo,S., Lee,B., Park,K., Song,J., Yu,J., Rheem,I., Kim,J., Hwang,S., Choi,Y., Cheon,D. and Park,J. | Virology Journal | 2011 |
| HQ685877 | Epidemics of enterovirus infection in Chungnam Korea, 2008 and 2009 | Baek,K., Yeo,S., Lee,B., Park,K., Song,J., Yu,J., Rheem,I., Kim,J., Hwang,S., Choi,Y., Cheon,D. and Park,J. | Virology Journal | 2011 |
| HQ685878 | Epidemics of enterovirus infection in Chungnam Korea, 2008 and 2009 | Baek,K., Yeo,S., Lee,B., Park,K., Song,J., Yu,J., Rheem,I., Kim,J., Hwang,S., Choi,Y., Cheon,D. and Park,J. | Virology Journal | 2011 |
| HQ694467 | Virological surveillance of enteroviruses from healthy children in the border areas with Myanma, Yunnan Province, China, 2009 | Tian,B.J., Tang,J.J., Zhang,J., Ding,Z.R. and Pang,Y.K. | Direct Genbank Submission | 2010 |
| HQ844650 | Virological surveillance of enteroviruses from healthy children in the border areas with Myanma, Yunnan Province, China, 2009 | Tian,B.J., Tang,J.J., Zhang,J., Ding,Z.R. and Pang,Y.K. | Direct Genbank Submission | 2010 |
| HQ844651 | Virological surveillance of enteroviruses from healthy children in the border areas with Myanma, Yunnan Province, China, 2009 | Tian,B.J., Tang,J.J., Zhang,J., Ding,Z.R. and Pang,Y.K. | Direct Genbank Submission | 2010 |
| JF773190 | Comparison of hand, foot and mouth disease and herpangina | Park,K., Baek,K., Kim,D., Lee,B., Park,J. and Choi,Y. | Direct Genbank Submission | 2011 |
| JF773191 | Comparison of hand, foot and mouth disease and herpangina | Park,K., Baek,K., Kim,D., Lee,B., Park,J. and Choi,Y. | Direct Genbank Submission | 2011 |
| JN169069 | Coxsackievirus a21, enterovirus 68, and acute respiratory tract infection, China | Xiang,Z., Gonzalez,R., Wang,Z., Ren,L., Xiao,Y., Li,J., Li,Y., Vernet,G., Paranhos-Baccala,G., Jin,Q. and Wang,J. | Emerging Infectious Disease | 2012 |
| JN169070 | Coxsackievirus a21, enterovirus 68, and acute respiratory tract infection, China | Xiang,Z., Gonzalez,R., Wang,Z., Ren,L., Xiao,Y., Li,J., Li,Y., Vernet,G., Paranhos-Baccala,G., Jin,Q. and Wang,J. | Emerging Infectious Disease | 2012 |
| JN169071 | Coxsackievirus a21, enterovirus 68, and acute respiratory tract infection, China | Xiang,Z., Gonzalez,R., Wang,Z., Ren,L., Xiao,Y., Li,J., Li,Y., Vernet,G., Paranhos-Baccala,G., Jin,Q. and Wang,J. | Emerging Infectious Disease | 2012 |
| JN169072 | Coxsackievirus a21, enterovirus 68, and acute respiratory tract infection, China | Xiang,Z., Gonzalez,R., Wang,Z., Ren,L., Xiao,Y., Li,J., Li,Y., Vernet,G., Paranhos-Baccala,G., Jin,Q. and Wang,J. | Emerging Infectious Disease | 2012 |
| JN203547 | Antigenic diversity of enteroviruses associated with nonpolio acute flaccid paralysis, India, 2007-2009 | Rao,C.D., Yergolkar,P. and Shankarappa,K.S. | Emerging Infectious Disease | 2012 |
| JN203548 | Antigenic diversity of enteroviruses associated with nonpolio acute flaccid paralysis, India, 2007-2009 | Rao,C.D., Yergolkar,P. and Shankarappa,K.S. | Emerging Infectious Disease | 2012 |
| JN203549 | Antigenic diversity of enteroviruses associated with nonpolio acute flaccid paralysis, India, 2007-2009 | Rao,C.D., Yergolkar,P. and Shankarappa,K.S. | Emerging Infectious Disease | 2012 |
| JN203550 | Antigenic diversity of enteroviruses associated with nonpolio acute flaccid paralysis, India, 2007-2009 | Rao,C.D., Yergolkar,P. and Shankarappa,K.S. | Emerging Infectious Disease | 2012 |
| JN203551 | Antigenic diversity of enteroviruses associated with nonpolio acute flaccid paralysis, India, 2007-2009 | Rao,C.D., Yergolkar,P. and Shankarappa,K.S. | Emerging Infectious Disease | 2012 |
| JN203552 | Antigenic diversity of enteroviruses associated with nonpolio acute flaccid paralysis, India, 2007-2009 | Rao,C.D., Yergolkar,P. and Shankarappa,K.S. | Emerging Infectious Disease | 2012 |
| JN203553 | Antigenic diversity of enteroviruses associated with nonpolio acute flaccid paralysis, India, 2007-2009 | Rao,C.D., Yergolkar,P. and Shankarappa,K.S. | Emerging Infectious Disease | 2012 |
| JN203554 | Antigenic diversity of enteroviruses associated with nonpolio acute flaccid paralysis, India, 2007-2009 | Rao,C.D., Yergolkar,P. and Shankarappa,K.S. | Emerging Infectious Disease | 2012 |
| JN203555 | Antigenic diversity of enteroviruses associated with nonpolio acute flaccid paralysis, India, 2007-2009 | Rao,C.D., Yergolkar,P. and Shankarappa,K.S. | Emerging Infectious Disease | 2012 |
| JN203556 | Antigenic diversity of enteroviruses associated with nonpolio acute flaccid paralysis, India, 2007-2009 | Rao,C.D., Yergolkar,P. and Shankarappa,K.S. | Emerging Infectious Disease | 2012 |
| JN203557 | Antigenic diversity of enteroviruses associated with nonpolio acute flaccid paralysis, India, 2007-2009 | Rao,C.D., Yergolkar,P. and Shankarappa,K.S. | Emerging Infectious Disease | 2012 |
| JN203558 | Antigenic diversity of enteroviruses associated with nonpolio acute flaccid paralysis, India, 2007-2009 | Rao,C.D., Yergolkar,P. and Shankarappa,K.S. | Emerging Infectious Disease | 2012 |
| JN203559 | Antigenic diversity of enteroviruses associated with nonpolio acute flaccid paralysis, India, 2007-2009 | Rao,C.D., Yergolkar,P. and Shankarappa,K.S. | Emerging Infectious Disease | 2012 |
| JN203560 | Antigenic diversity of enteroviruses associated with nonpolio acute flaccid paralysis, India, 2007-2009 | Rao,C.D., Yergolkar,P. and Shankarappa,K.S. | Emerging Infectious Disease | 2012 |
| JN203561 | Antigenic diversity of enteroviruses associated with nonpolio acute flaccid paralysis, India, 2007-2009 | Rao,C.D., Yergolkar,P. and Shankarappa,K.S. | Emerging Infectious Disease | 2012 |
| JN203562 | Antigenic diversity of enteroviruses associated with nonpolio acute flaccid paralysis, India, 2007-2009 | Rao,C.D., Yergolkar,P. and Shankarappa,K.S. | Emerging Infectious Disease | 2012 |
| JN203563 | Antigenic diversity of enteroviruses associated with nonpolio acute flaccid paralysis, India, 2007-2009 | Rao,C.D., Yergolkar,P. and Shankarappa,K.S. | Emerging Infectious Disease | 2012 |
| JN203564 | Antigenic diversity of enteroviruses associated with nonpolio acute flaccid paralysis, India, 2007-2009 | Rao,C.D., Yergolkar,P. and Shankarappa,K.S. | Emerging Infectious Disease | 2012 |
| JN203565 | Antigenic diversity of enteroviruses associated with nonpolio acute flaccid paralysis, India, 2007-2009 | Rao,C.D., Yergolkar,P. and Shankarappa,K.S. | Emerging Infectious Disease | 2012 |
| JN203566 | Antigenic diversity of enteroviruses associated with nonpolio acute flaccid paralysis, India, 2007-2009 | Rao,C.D., Yergolkar,P. and Shankarappa,K.S. | Emerging Infectious Disease | 2012 |
| JN203567 | Antigenic diversity of enteroviruses associated with nonpolio acute flaccid paralysis, India, 2007-2009 | Rao,C.D., Yergolkar,P. and Shankarappa,K.S. | Emerging Infectious Disease | 2012 |
| JN255592 | Molecular characterization of human enteroviruses in the central african republic: uncovering wide diversity and identification of a new human enterovirus a71 genogroup | Bessaud,M., Pillet,S., Ibrahim,W., Joffret,M.L., Pozzetto,B., Delpeyroux,F. and Gouandjika-Vasilache,I. | Journal of Clinical Microbiology | 2012 |
| JN572153 | Analysis of the VP1 gene of coxsackievirus B1 from viral encephalitis prevailing isolate from Zhejiang Province in 2009 | Yan,J. and Shi,W. | Direct Genbank Submission | 2011 |
| JN638300 | Molecular characteristics of human coxsackievirus B1 infection in Korea, 2008-2009 | Kim,H., Kang,B., Hwang,S., Hong,J., Chung,J., Kim,S., Jeong,Y.S., Kim,K. and Cheon,D.S. | Journal of Medical Virology | 2013 |
| JN638301 | Molecular characteristics of human coxsackievirus B1 infection in Korea, 2008-2009 | Kim,H., Kang,B., Hwang,S., Hong,J., Chung,J., Kim,S., Jeong,Y.S., Kim,K. and Cheon,D.S. | Journal of Medical Virology | 2013 |
| JN638302 | Molecular characteristics of human coxsackievirus B1 infection in Korea, 2008-2009 | Kim,H., Kang,B., Hwang,S., Hong,J., Chung,J., Kim,S., Jeong,Y.S., Kim,K. and Cheon,D.S. | Journal of Medical Virology | 2013 |
| JN638303 | Molecular characteristics of human coxsackievirus B1 infection in Korea, 2008-2009 | Kim,H., Kang,B., Hwang,S., Hong,J., Chung,J., Kim,S., Jeong,Y.S., Kim,K. and Cheon,D.S. | Journal of Medical Virology | 2013 |
| JN638304 | Molecular characteristics of human coxsackievirus B1 infection in Korea, 2008-2009 | Kim,H., Kang,B., Hwang,S., Hong,J., Chung,J., Kim,S., Jeong,Y.S., Kim,K. and Cheon,D.S. | Journal of Medical Virology | 2013 |
| JN638305 | Molecular characteristics of human coxsackievirus B1 infection in Korea, 2008-2009 | Kim,H., Kang,B., Hwang,S., Hong,J., Chung,J., Kim,S., Jeong,Y.S., Kim,K. and Cheon,D.S. | Journal of Medical Virology | 2013 |
| JN638306 | Molecular characteristics of human coxsackievirus B1 infection in Korea, 2008-2009 | Kim,H., Kang,B., Hwang,S., Hong,J., Chung,J., Kim,S., Jeong,Y.S., Kim,K. and Cheon,D.S. | Journal of Medical Virology | 2013 |
| JN638307 | Molecular characteristics of human coxsackievirus B1 infection in Korea, 2008-2009 | Kim,H., Kang,B., Hwang,S., Hong,J., Chung,J., Kim,S., Jeong,Y.S., Kim,K. and Cheon,D.S. | Journal of Medical Virology | 2013 |
| JN638308 | Molecular characteristics of human coxsackievirus B1 infection in Korea, 2008-2009 | Kim,H., Kang,B., Hwang,S., Hong,J., Chung,J., Kim,S., Jeong,Y.S., Kim,K. and Cheon,D.S. | Journal of Medical Virology | 2013 |
| JN638309 | Molecular characteristics of human coxsackievirus B1 infection in Korea, 2008-2009 | Kim,H., Kang,B., Hwang,S., Hong,J., Chung,J., Kim,S., Jeong,Y.S., Kim,K. and Cheon,D.S. | Journal of Medical Virology | 2013 |
| JN638310 | Molecular characteristics of human coxsackievirus B1 infection in Korea, 2008-2009 | Kim,H., Kang,B., Hwang,S., Hong,J., Chung,J., Kim,S., Jeong,Y.S., Kim,K. and Cheon,D.S. | Journal of Medical Virology | 2013 |
| JN896800 | Comparing molecular methods for early detection and serotyping of enteroviruses in throat swabs of pediatric patients | Chiang,P.S., Huang,M.L., Luo,S.T., Lin,T.Y., Tsao,K.C. and Lee,M.S. | PlosOne | 2012 |
| JN990934 | Molecular epidemiological study of human enterovirus circulating in India | Kumar,A., Shukla,D., Idris,M.Z., Srivastava,S. and Dhole,T.N. | Direct Genbank Submission | 2010 |
| JQ235663 | Molecular characteristics of human coxsackievirus B1 infection in Korea, 2008-2009 | Kim,H., Kang,B., Hwang,S., Hong,J., Chung,J., Kim,S., Jeong,Y.S., Kim,K. and Cheon,D.S. | Journal of Medical Virology | 2013 |
| JQ235664 | Molecular characteristics of human coxsackievirus B1 infection in Korea, 2008-2009 | Kim,H., Kang,B., Hwang,S., Hong,J., Chung,J., Kim,S., Jeong,Y.S., Kim,K. and Cheon,D.S. | Journal of Medical Virology | 2013 |
| JQ235665 | Molecular characteristics of human coxsackievirus B1 infection in Korea, 2008-2009 | Kim,H., Kang,B., Hwang,S., Hong,J., Chung,J., Kim,S., Jeong,Y.S., Kim,K. and Cheon,D.S. | Journal of Medical Virology | 2013 |
| JQ235666 | Molecular characteristics of human coxsackievirus B1 infection in Korea, 2008-2009 | Kim,H., Kang,B., Hwang,S., Hong,J., Chung,J., Kim,S., Jeong,Y.S., Kim,K. and Cheon,D.S. | Journal of Medical Virology | 2013 |
| JQ235667 | Molecular characteristics of human coxsackievirus B1 infection in Korea, 2008-2009 | Kim,H., Kang,B., Hwang,S., Hong,J., Chung,J., Kim,S., Jeong,Y.S., Kim,K. and Cheon,D.S. | Journal of Medical Virology | 2013 |
| JQ235668 | Molecular characteristics of human coxsackievirus B1 infection in Korea, 2008-2009 | Kim,H., Kang,B., Hwang,S., Hong,J., Chung,J., Kim,S., Jeong,Y.S., Kim,K. and Cheon,D.S. | Journal of Medical Virology | 2013 |
| JQ235669 | Molecular characteristics of human coxsackievirus B1 infection in Korea, 2008-2009 | Kim,H., Kang,B., Hwang,S., Hong,J., Chung,J., Kim,S., Jeong,Y.S., Kim,K. and Cheon,D.S. | Journal of Medical Virology | 2013 |
| JQ235670 | Molecular characteristics of human coxsackievirus B1 infection in Korea, 2008-2009 | Kim,H., Kang,B., Hwang,S., Hong,J., Chung,J., Kim,S., Jeong,Y.S., Kim,K. and Cheon,D.S. | Journal of Medical Virology | 2013 |
| JQ235671 | Molecular characteristics of human coxsackievirus B1 infection in Korea, 2008-2009 | Kim,H., Kang,B., Hwang,S., Hong,J., Chung,J., Kim,S., Jeong,Y.S., Kim,K. and Cheon,D.S. | Journal of Medical Virology | 2013 |
| JQ235672 | Molecular characteristics of human coxsackievirus B1 infection in Korea, 2008-2009 | Kim,H., Kang,B., Hwang,S., Hong,J., Chung,J., Kim,S., Jeong,Y.S., Kim,K. and Cheon,D.S. | Journal of Medical Virology | 2013 |
| JQ235673 | Molecular characteristics of human coxsackievirus B1 infection in Korea, 2008-2009 | Kim,H., Kang,B., Hwang,S., Hong,J., Chung,J., Kim,S., Jeong,Y.S., Kim,K. and Cheon,D.S. | Journal of Medical Virology | 2013 |
| JQ235674 | Molecular characteristics of human coxsackievirus B1 infection in Korea, 2008-2009 | Kim,H., Kang,B., Hwang,S., Hong,J., Chung,J., Kim,S., Jeong,Y.S., Kim,K. and Cheon,D.S. | Journal of Medical Virology | 2013 |
| JQ235675 | Molecular characteristics of human coxsackievirus B1 infection in Korea, 2008-2009 | Kim,H., Kang,B., Hwang,S., Hong,J., Chung,J., Kim,S., Jeong,Y.S., Kim,K. and Cheon,D.S. | Journal of Medical Virology | 2013 |
| JQ235676 | Molecular characteristics of human coxsackievirus B1 infection in Korea, 2008-2009 | Kim,H., Kang,B., Hwang,S., Hong,J., Chung,J., Kim,S., Jeong,Y.S., Kim,K. and Cheon,D.S. | Journal of Medical Virology | 2013 |
| JQ235677 | Molecular characteristics of human coxsackievirus B1 infection in Korea, 2008-2009 | Kim,H., Kang,B., Hwang,S., Hong,J., Chung,J., Kim,S., Jeong,Y.S., Kim,K. and Cheon,D.S. | Journal of Medical Virology | 2013 |
| JQ235678 | Molecular characteristics of human coxsackievirus B1 infection in Korea, 2008-2009 | Kim,H., Kang,B., Hwang,S., Hong,J., Chung,J., Kim,S., Jeong,Y.S., Kim,K. and Cheon,D.S. | Journal of Medical Virology | 2013 |
| JQ235679 | Molecular characteristics of human coxsackievirus B1 infection in Korea, 2008-2009 | Kim,H., Kang,B., Hwang,S., Hong,J., Chung,J., Kim,S., Jeong,Y.S., Kim,K. and Cheon,D.S. | Journal of Medical Virology | 2013 |
| JQ235680 | Molecular characteristics of human coxsackievirus B1 infection in Korea, 2008-2009 | Kim,H., Kang,B., Hwang,S., Hong,J., Chung,J., Kim,S., Jeong,Y.S., Kim,K. and Cheon,D.S. | Journal of Medical Virology | 2013 |
| JQ235681 | Molecular characteristics of human coxsackievirus B1 infection in Korea, 2008-2009 | Kim,H., Kang,B., Hwang,S., Hong,J., Chung,J., Kim,S., Jeong,Y.S., Kim,K. and Cheon,D.S. | Journal of Medical Virology | 2013 |
| JQ235682 | Molecular characteristics of human coxsackievirus B1 infection in Korea, 2008-2009 | Kim,H., Kang,B., Hwang,S., Hong,J., Chung,J., Kim,S., Jeong,Y.S., Kim,K. and Cheon,D.S. | Journal of Medical Virology | 2013 |
| JQ235683 | Molecular characteristics of human coxsackievirus B1 infection in Korea, 2008-2009 | Kim,H., Kang,B., Hwang,S., Hong,J., Chung,J., Kim,S., Jeong,Y.S., Kim,K. and Cheon,D.S. | Journal of Medical Virology | 2013 |
| JQ235684 | Molecular characteristics of human coxsackievirus B1 infection in Korea, 2008-2009 | Kim,H., Kang,B., Hwang,S., Hong,J., Chung,J., Kim,S., Jeong,Y.S., Kim,K. and Cheon,D.S. | Journal of Medical Virology | 2013 |
| JQ235685 | Molecular characteristics of human coxsackievirus B1 infection in Korea, 2008-2009 | Kim,H., Kang,B., Hwang,S., Hong,J., Chung,J., Kim,S., Jeong,Y.S., Kim,K. and Cheon,D.S. | Journal of Medical Virology | 2013 |
| JQ235686 | Molecular characteristics of human coxsackievirus B1 infection in Korea, 2008-2009 | Kim,H., Kang,B., Hwang,S., Hong,J., Chung,J., Kim,S., Jeong,Y.S., Kim,K. and Cheon,D.S. | Journal of Medical Virology | 2013 |
| JQ235687 | Molecular characteristics of human coxsackievirus B1 infection in Korea, 2008-2009 | Kim,H., Kang,B., Hwang,S., Hong,J., Chung,J., Kim,S., Jeong,Y.S., Kim,K. and Cheon,D.S. | Journal of Medical Virology | 2013 |
| JQ235688 | Molecular characteristics of human coxsackievirus B1 infection in Korea, 2008-2009 | Kim,H., Kang,B., Hwang,S., Hong,J., Chung,J., Kim,S., Jeong,Y.S., Kim,K. and Cheon,D.S. | Journal of Medical Virology | 2013 |
| JQ235689 | Molecular characteristics of human coxsackievirus B1 infection in Korea, 2008-2009 | Kim,H., Kang,B., Hwang,S., Hong,J., Chung,J., Kim,S., Jeong,Y.S., Kim,K. and Cheon,D.S. | Journal of Medical Virology | 2013 |
| JQ239014 | Early human enterovirus infections in healthy Swedish children participating in the PRODIA pilot study | Simonen-Tikka,M.L., Hiekka,A.K., Klemola,P., Poussa,T., Ludvigsson,J., Korpela,R., Vaarala,O. and Roivainen,M. | Journal of Medical Virology | 2012 |
| JQ239015 | Early human enterovirus infections in healthy Swedish children participating in the PRODIA pilot study | Simonen-Tikka,M.L., Hiekka,A.K., Klemola,P., Poussa,T., Ludvigsson,J., Korpela,R., Vaarala,O. and Roivainen,M. | Journal of Medical Virology | 2012 |
| JQ713883 | Epidemics and Frequent Recombination within Species in Outbreaks of Human Enterovirus B-Associated Hand, Foot and Mouth Disease in Shandong China in 2010 and 2011 | Zhang,T., Du,J., Xue,Y., Su,H., Yang,F. and Jin,Q. | PLosOne | 2013 |
| JQ740124 | Molecular epidemiological study of enteroviruses associated with encephalitis in children from India | Kumar,A., Shukla,D., Kumar,R., Idris,M.Z., Misra,U.K. and Dhole,T.N. | Journal of Clinical Microbiology | 2012 |
| JQ740125 | Molecular epidemiological study of enteroviruses associated with encephalitis in children from India | Kumar,A., Shukla,D., Kumar,R., Idris,M.Z., Misra,U.K. and Dhole,T.N. | Journal of Clinical Microbiology | 2012 |
| JQ740126 | Molecular epidemiological study of enteroviruses associated with encephalitis in children from India | Kumar,A., Shukla,D., Kumar,R., Idris,M.Z., Misra,U.K. and Dhole,T.N. | Journal of Clinical Microbiology | 2012 |
| JQ740127 | Molecular epidemiological study of enteroviruses associated with encephalitis in children from India | Kumar,A., Shukla,D., Kumar,R., Idris,M.Z., Misra,U.K. and Dhole,T.N. | Journal of Clinical Microbiology | 2012 |
| JQ740128 | Molecular epidemiological study of enteroviruses associated with encephalitis in children from India | Kumar,A., Shukla,D., Kumar,R., Idris,M.Z., Misra,U.K. and Dhole,T.N. | Journal of Clinical Microbiology | 2012 |
| JQ740129 | Molecular epidemiological study of enteroviruses associated with encephalitis in children from India | Kumar,A., Shukla,D., Kumar,R., Idris,M.Z., Misra,U.K. and Dhole,T.N. | Journal of Clinical Microbiology | 2012 |
| JX009057 | Genotyping of enteroviruses in Denmark 2008 and 2009: comparison of methods targeting the VP1 and VP2 regions for direct genotyping of clinical samples | Midgley,S., Fonager,J. and Bottiger,B. | Direct Genbank Submission | 2012 |
| JX009076 | Genotyping of enteroviruses in Denmark 2008 and 2009: comparison of methods targeting the VP1 and VP2 regions for direct genotyping of clinical samples | Midgley,S., Fonager,J. and Bottiger,B. | Direct Genbank Submission | 2012 |
| JX009082 | Genotyping of enteroviruses in Denmark 2008 and 2009: comparison of methods targeting the VP1 and VP2 regions for direct genotyping of clinical samples | Midgley,S., Fonager,J. and Bottiger,B. | Direct Genbank Submission | 2012 |
| JX009085 | Genotyping of enteroviruses in Denmark 2008 and 2009: comparison of methods targeting the VP1 and VP2 regions for direct genotyping of clinical samples | Midgley,S., Fonager,J. and Bottiger,B. | Direct Genbank Submission | 2012 |
| JX009090 | Genotyping of enteroviruses in Denmark 2008 and 2009: comparison of methods targeting the VP1 and VP2 regions for direct genotyping of clinical samples | Midgley,S., Fonager,J. and Bottiger,B. | Direct Genbank Submission | 2012 |
| JX181913 | Molecular identification and analysis of human enteroviruses isolated from healthy children in shenzhen, china from 2010 to 2011 | Wu,W., Xu,W.B., Chen,L., Chen,H.L., Liu,Q., Wang,D.L., Chen,Y.J.,  Yao,W., Li,G., Feng,B., Shu,B.H., Zhou,Y.K. and He,Y.Q. | PLosOne | 2013 |
| JX181914 | Molecular identification and analysis of human enteroviruses isolated from healthy children in shenzhen, china from 2010 to 2011 | Wu,W., Xu,W.B., Chen,L., Chen,H.L., Liu,Q., Wang,D.L., Chen,Y.J.,  Yao,W., Li,G., Feng,B., Shu,B.H., Zhou,Y.K. and He,Y.Q. | PLosOne | 2013 |
| JX417717 | High frequency and diversity of species C enteroviruses in Cameroon and neighboring countries | Sadeuh-Mba,S.A., Bessaud,M., Massenet,D., Joffret,M.L., Endegue,M.C., Njouom,R., Reynes,J.M., Rousset,D. and Delpeyroux,F. | Journal of Clinical Virology | 2013 |
| JX417718 | High frequency and diversity of species C enteroviruses in Cameroon and neighboring countries | Sadeuh-Mba,S.A., Bessaud,M., Massenet,D., Joffret,M.L., Endegue,M.C., Njouom,R., Reynes,J.M., Rousset,D. and Delpeyroux,F. | Journal of Clinical Virology | 2013 |
| JX417719 | High frequency and diversity of species C enteroviruses in Cameroon and neighboring countries | Sadeuh-Mba,S.A., Bessaud,M., Massenet,D., Joffret,M.L., Endegue,M.C., Njouom,R., Reynes,J.M., Rousset,D. and Delpeyroux,F. | Journal of Clinical Virology | 2013 |
| JX417720 | High frequency and diversity of species C enteroviruses in Cameroon and neighboring countries | Sadeuh-Mba,S.A., Bessaud,M., Massenet,D., Joffret,M.L., Endegue,M.C., Njouom,R., Reynes,J.M., Rousset,D. and Delpeyroux,F. | Journal of Clinical Virology | 2013 |
| JX417721 | High frequency and diversity of species C enteroviruses in Cameroon and neighboring countries | Sadeuh-Mba,S.A., Bessaud,M., Massenet,D., Joffret,M.L., Endegue,M.C., Njouom,R., Reynes,J.M., Rousset,D. and Delpeyroux,F. | Journal of Clinical Virology | 2013 |
| JX437641 | High frequency and diversity of species C enteroviruses in Cameroon and neighboring countries | Sadeuh-Mba,S.A., Bessaud,M., Massenet,D., Joffret,M.L., Endegue,M.C., Njouom,R., Reynes,J.M., Rousset,D. and Delpeyroux,F. | Journal of Clinical Virology | 2013 |
| JX476149 | Characterization of the non-polio enterovirus infections associated with acute flaccid paralysis in South-Western India | Laxmivandana,R., Yergolkar,P., Gopalkrishna,V. and Chitambar,S.D. | PLosOne | 2013 |
| JX476150 | Characterization of the non-polio enterovirus infections associated with acute flaccid paralysis in South-Western India | Laxmivandana,R., Yergolkar,P., Gopalkrishna,V. and Chitambar,S.D. | PLosOne | 2013 |
| JX476151 | Characterization of the non-polio enterovirus infections associated with acute flaccid paralysis in South-Western India | Laxmivandana,R., Yergolkar,P., Gopalkrishna,V. and Chitambar,S.D. | PLosOne | 2013 |
| JX476152 | Characterization of the non-polio enterovirus infections associated with acute flaccid paralysis in South-Western India | Laxmivandana,R., Yergolkar,P., Gopalkrishna,V. and Chitambar,S.D. | PLosOne | 2013 |
| JX476153 | Characterization of the non-polio enterovirus infections associated with acute flaccid paralysis in South-Western India | Laxmivandana,R., Yergolkar,P., Gopalkrishna,V. and Chitambar,S.D. | PLosOne | 2013 |
| JX476154 | Characterization of the non-polio enterovirus infections associated with acute flaccid paralysis in South-Western India | Laxmivandana,R., Yergolkar,P., Gopalkrishna,V. and Chitambar,S.D. | PLosOne | 2013 |
| JX476155 | Characterization of the non-polio enterovirus infections associated with acute flaccid paralysis in South-Western India | Laxmivandana,R., Yergolkar,P., Gopalkrishna,V. and Chitambar,S.D. | PLosOne | 2013 |
| JX513568 | Non-polio enteroviruses and their association with acute diarrhea in children in India | Rao,D.C., Ananda Babu,M., Raghavendra,A., Dhananjaya,D., Kumar,S. and Maiya,P.P. | Infection, Genetics and Evolution | 2013 |
| JX513569 | Non-polio enteroviruses and their association with acute diarrhea in children in India | Rao,D.C., Ananda Babu,M., Raghavendra,A., Dhananjaya,D., Kumar,S. and Maiya,P.P. | Infection, Genetics and Evolution | 2013 |
| JX538148 | Characterizing the Picornavirus Landscape among Synanthropic Nonhuman Primates in Bangladesh, 2007 to 2008 | Oberste,M.S., Feeroz,M.M., Maher,K., Nix,W.A., Engel,G.A.,  Hasan,K.M., Begum,S., Oh,G., Chowdhury,A.H., Pallansch,M.A. and Jones-Engel,L. | Journal of Virology | 2013 |
| JX538149 | Characterizing the Picornavirus Landscape among Synanthropic Nonhuman Primates in Bangladesh, 2007 to 2008 | Oberste,M.S., Feeroz,M.M., Maher,K., Nix,W.A., Engel,G.A.,  Hasan,K.M., Begum,S., Oh,G., Chowdhury,A.H., Pallansch,M.A. and Jones-Engel,L. | Journal of Virology | 2013 |
| KC411823 | Epidemics and Frequent Recombination within Species in Outbreaks of  Human Enterovirus B-Associated Hand, Foot and Mouth Disease in Shandong China in 2010 and 2011 | Zhang,T., Du,J., Xue,Y., Su,H., Yang,F. and Jin,Q. | PLosOne | 2013 |
| KC411824 | Epidemics and Frequent Recombination within Species in Outbreaks of  Human Enterovirus B-Associated Hand, Foot and Mouth Disease in Shandong China in 2010 and 2011 | Zhang,T., Du,J., Xue,Y., Su,H., Yang,F. and Jin,Q. | PLosOne | 2013 |
| KC411825 | Epidemics and Frequent Recombination within Species in Outbreaks of  Human Enterovirus B-Associated Hand, Foot and Mouth Disease in Shandong China in 2010 and 2011 | Zhang,T., Du,J., Xue,Y., Su,H., Yang,F. and Jin,Q. | PLosOne | 2013 |
| KC867080 | Emergence, circulation, and spatiotemporal phylogenetic analysis of coxsackievirus a6- and coxsackievirus a10-associated hand, foot, and mouth disease infections from 2008 to 2012 in shenzhen, china | He,Y.Q., Chen,L., Xu,W.B., Yang,H., Wang,H.Z., Zong,W.P.,  Xian,H.X., Chen,H.L., Yao,X.J., Hu,Z.L., Luo,M., Zhang,H.L., Ma,H.W., Cheng,J.Q., Feng,Q.J. and Zhao,D.J. | Journal of Clinical Microbiology | 2013 |
| KF150151 | Molecular epidemiology of human enterovirus associated with aseptic meningitis in shandong province, china, 2006-2012 | Tao,Z., Wang,H., Li,Y., Liu,G., Xu,A., Lin,X., Song,L., Ji,F., Wang,S., Cui,N. and Song,Y. | PLosOne | 2014 |
| KF150152 | Molecular epidemiology of human enterovirus associated with aseptic meningitis in shandong province, china, 2006-2012 | Tao,Z., Wang,H., Li,Y., Liu,G., Xu,A., Lin,X., Song,L., Ji,F., Wang,S., Cui,N. and Song,Y. | PLosOne | 2014 |
| KF150153 | Molecular epidemiology of human enterovirus associated with aseptic meningitis in shandong province, china, 2006-2012 | Tao,Z., Wang,H., Li,Y., Liu,G., Xu,A., Lin,X., Song,L., Ji,F., Wang,S., Cui,N. and Song,Y. | PLosOne | 2014 |
| KF150154 | Molecular epidemiology of human enterovirus associated with aseptic meningitis in shandong province, china, 2006-2012 | Tao,Z., Wang,H., Li,Y., Liu,G., Xu,A., Lin,X., Song,L., Ji,F., Wang,S., Cui,N. and Song,Y. | PLosOne | 2014 |
| KF150155 | Molecular epidemiology of human enterovirus associated with aseptic meningitis in shandong province, china, 2006-2012 | Tao,Z., Wang,H., Li,Y., Liu,G., Xu,A., Lin,X., Song,L., Ji,F., Wang,S., Cui,N. and Song,Y. | PLosOne | 2014 |
| KF150156 | Molecular epidemiology of human enterovirus associated with aseptic meningitis in shandong province, china, 2006-2012 | Tao,Z., Wang,H., Li,Y., Liu,G., Xu,A., Lin,X., Song,L., Ji,F., Wang,S., Cui,N. and Song,Y. | PLosOne | 2014 |
| KF199866 | Enteric virus in coastal water of Northern Morocco | Amar,L. and Lkhider,M. | Direct Genbank Submission | 2013 |
| KF199867 | Enteric virus in coastal water of Northern Morocco | Amar,L. and Lkhider,M. | Direct Genbank Submission | 2013 |
| KF199868 | Enteric virus in coastal water of Northern Morocco | Amar,L. and Lkhider,M. | Direct Genbank Submission | 2013 |
| KF199869 | Enteric virus in coastal water of Northern Morocco | Amar,L. and Lkhider,M. | Direct Genbank Submission | 2013 |
| KF246749 | Molecular epidemiology of human enterovirus associated with aseptic meningitis in shandong province, china, 2006-2012 | Tao,Z., Wang,H., Li,Y., Liu,G., Xu,A., Lin,X., Song,L., Ji,F., Wang,S., Cui,N. and Song,Y. | PLosOne | 2014 |
| KF246750 | Molecular epidemiology of human enterovirus associated with aseptic meningitis in shandong province, china, 2006-2012 | Tao,Z., Wang,H., Li,Y., Liu,G., Xu,A., Lin,X., Song,L., Ji,F., Wang,S., Cui,N. and Song,Y. | PLosOne | 2014 |
| KF383382 | Prevalence and Characterization of Enterovirus Infections among  Pediatric Patients with Hand Foot Mouth Disease, Herpangina and Influenza Like Illness in Thailand, 2012 | Puenpa,J., Mauleekoonphairoj,J., Linsuwanon,P., Suwannakarn,K., Chieochansin,T., Korkong,S., Theamboonlers,A. and Poovorawan,Y. | PLosOne | 2014 |
| JF577980 |  |  |  |  |
| KF661141 | Prevalence and Characterization of Enterovirus Infections among  Pediatric Patients with Hand Foot Mouth Disease, Herpangina and Influenza Like Illness in Thailand, 2012 | Puenpa,J., Mauleekoonphairoj,J., Linsuwanon,P., Suwannakarn,K., Chieochansin,T., Korkong,S., Theamboonlers,A. and Poovorawan,Y. | PLosOne | 2014 |
| KF661142 | Prevalence and Characterization of Enterovirus Infections among  Pediatric Patients with Hand Foot Mouth Disease, Herpangina and Influenza Like Illness in Thailand, 2012 | Puenpa,J., Mauleekoonphairoj,J., Linsuwanon,P., Suwannakarn,K., Chieochansin,T., Korkong,S., Theamboonlers,A. and Poovorawan,Y. | PLosOne | 2014 |
| KF962544 | Non-rhinovirus enteroviruses associated with respiratory infections in Peru (2005-2010) | Huaman,J.L., Carrion,G., Ampuero,J.S., Gomez,J., Ocana,V., Paz,I., Gomez,E., Chavez,E., Sarmiento,F., Pozo,E., Laguna-Torres,V.A. and Halsey,E.S. | Virology Journal | 2014 |
| KF962545 | Non-rhinovirus enteroviruses associated with respiratory infections in Peru (2005-2010) | Huaman,J.L., Carrion,G., Ampuero,J.S., Gomez,J., Ocana,V., Paz,I., Gomez,E., Chavez,E., Sarmiento,F., Pozo,E., Laguna-Torres,V.A. and Halsey,E.S. | Virology Journal | 2014 |
| KF962546 | Non-rhinovirus enteroviruses associated with respiratory infections in Peru (2005-2010) | Huaman,J.L., Carrion,G., Ampuero,J.S., Gomez,J., Ocana,V., Paz,I., Gomez,E., Chavez,E., Sarmiento,F., Pozo,E., Laguna-Torres,V.A. and Halsey,E.S. | Virology Journal | 2014 |
| KF962547 | Non-rhinovirus enteroviruses associated with respiratory infections in Peru (2005-2010) | Huaman,J.L., Carrion,G., Ampuero,J.S., Gomez,J., Ocana,V., Paz,I., Gomez,E., Chavez,E., Sarmiento,F., Pozo,E., Laguna-Torres,V.A. and Halsey,E.S. | Virology Journal | 2014 |
| KF962548 | Non-rhinovirus enteroviruses associated with respiratory infections in Peru (2005-2010) | Huaman,J.L., Carrion,G., Ampuero,J.S., Gomez,J., Ocana,V., Paz,I., Gomez,E., Chavez,E., Sarmiento,F., Pozo,E., Laguna-Torres,V.A. and Halsey,E.S. | Virology Journal | 2014 |
| KF962549 | Non-rhinovirus enteroviruses associated with respiratory infections in Peru (2005-2010) | Huaman,J.L., Carrion,G., Ampuero,J.S., Gomez,J., Ocana,V., Paz,I., Gomez,E., Chavez,E., Sarmiento,F., Pozo,E., Laguna-Torres,V.A. and Halsey,E.S. | Virology Journal | 2014 |
| KF962550 | Non-rhinovirus enteroviruses associated with respiratory infections in Peru (2005-2010) | Huaman,J.L., Carrion,G., Ampuero,J.S., Gomez,J., Ocana,V., Paz,I., Gomez,E., Chavez,E., Sarmiento,F., Pozo,E., Laguna-Torres,V.A. and Halsey,E.S. | Virology Journal | 2014 |
| KF962551 | Non-rhinovirus enteroviruses associated with respiratory infections in Peru (2005-2010) | Huaman,J.L., Carrion,G., Ampuero,J.S., Gomez,J., Ocana,V., Paz,I., Gomez,E., Chavez,E., Sarmiento,F., Pozo,E., Laguna-Torres,V.A. and Halsey,E.S. | Virology Journal | 2014 |
| KF962552 | Non-rhinovirus enteroviruses associated with respiratory infections in Peru (2005-2010) | Huaman,J.L., Carrion,G., Ampuero,J.S., Gomez,J., Ocana,V., Paz,I., Gomez,E., Chavez,E., Sarmiento,F., Pozo,E., Laguna-Torres,V.A. and Halsey,E.S. | Virology Journal | 2014 |
| KF962553 | Non-rhinovirus enteroviruses associated with respiratory infections in Peru (2005-2010) | Huaman,J.L., Carrion,G., Ampuero,J.S., Gomez,J., Ocana,V., Paz,I., Gomez,E., Chavez,E., Sarmiento,F., Pozo,E., Laguna-Torres,V.A. and Halsey,E.S. | Virology Journal | 2014 |
| KF962554 | Non-rhinovirus enteroviruses associated with respiratory infections in Peru (2005-2010) | Huaman,J.L., Carrion,G., Ampuero,J.S., Gomez,J., Ocana,V., Paz,I., Gomez,E., Chavez,E., Sarmiento,F., Pozo,E., Laguna-Torres,V.A. and Halsey,E.S. | Virology Journal | 2014 |
| KJ472837 | Genotyping of enteroviruses isolated in Kenya from pediatric patients using partial VP1 region | Opanda,S.M., Wamunyokoli,F., Khamadi,S., Coldren,R. and Bulimo,W.D. | Springerplus | 2016 |
| KJ472838 | Genotyping of enteroviruses isolated in Kenya from pediatric patients using partial VP1 region | Opanda,S.M., Wamunyokoli,F., Khamadi,S., Coldren,R. and Bulimo,W.D. | Springerplus | 2016 |
| KJ472839 | Genotyping of enteroviruses isolated in Kenya from pediatric patients using partial VP1 region | Opanda,S.M., Wamunyokoli,F., Khamadi,S., Coldren,R. and Bulimo,W.D. | Springerplus | 2016 |
| KJ472840 | Genotyping of enteroviruses isolated in Kenya from pediatric patients using partial VP1 region | Opanda,S.M., Wamunyokoli,F., Khamadi,S., Coldren,R. and Bulimo,W.D. | Springerplus | 2016 |
| KJ472841 | Genotyping of enteroviruses isolated in Kenya from pediatric patients using partial VP1 region | Opanda,S.M., Wamunyokoli,F., Khamadi,S., Coldren,R. and Bulimo,W.D. | Springerplus | 2016 |
| KJ472842 | Genotyping of enteroviruses isolated in Kenya from pediatric patients using partial VP1 region | Opanda,S.M., Wamunyokoli,F., Khamadi,S., Coldren,R. and Bulimo,W.D. | Springerplus | 2016 |
| KJ472843 | Genotyping of enteroviruses isolated in Kenya from pediatric patients using partial VP1 region | Opanda,S.M., Wamunyokoli,F., Khamadi,S., Coldren,R. and Bulimo,W.D. | Springerplus | 2016 |
| KJ472844 | Genotyping of enteroviruses isolated in Kenya from pediatric patients using partial VP1 region | Opanda,S.M., Wamunyokoli,F., Khamadi,S., Coldren,R. and Bulimo,W.D. | Springerplus | 2016 |
| KJ472845 | Genotyping of enteroviruses isolated in Kenya from pediatric patients using partial VP1 region | Opanda,S.M., Wamunyokoli,F., Khamadi,S., Coldren,R. and Bulimo,W.D. | Springerplus | 2016 |
| KJ484535 | Phylogenetics of an echovirus 30 outbreak | Yan,Q. | Direct Genbank Submission | 2014 |
| KJ484536 | Phylogenetics of an echovirus 30 outbreak | Yan,Q. | Direct Genbank Submission | 2014 |
| KJ484537 | Phylogenetics of an echovirus 30 outbreak | Yan,Q. | Direct Genbank Submission | 2014 |
| KJ484538 | Phylogenetics of an echovirus 30 outbreak | Yan,Q. | Direct Genbank Submission | 2014 |
| KJ484539 | Phylogenetics of an echovirus 30 outbreak | Yan,Q. | Direct Genbank Submission | 2014 |
| KP006027 | NA | Yun,H. | Direct Genbank Submission | 2014 |
| LC013462 | Molecular detection of enteroviruses other than EV-A71 and CV-A16 from hand, foot and mouth disease in Yunnan Province, 2014 | Tian,B.T. | Direct Genbank Submission | 2014 |
