# Supplementary material for: In silico and ex vivo approaches indicate immune pressure on capsid and non-capsid regions of coxsackie B viruses in the human system
Source: PLoS One. 2018 Jun 20;13(6):e0199323. doi: 10.1371/journal.pone.0199323 (PMC6010236; doi:10.1371/journal.pone.0199323)
Supplement: S4 Table — The sites of positive selection identified in CBV1 VP1 sequences available on Genbank are presented, along with the predominant amino acid present at that site and any HLA-A*02:01 binding epitopes contained within the site. (DOCX) [file pone.0199323.s005.docx]

S4 Table: Sites of Positive Selection Identified in CBV1 VP1 by MEME.

| Codon | p-value | Consensus Identity | HLA-A2 Epitopes |
| --- | --- | --- | --- |
| 583 | 0.003672 | V |  |
| 610 | 1.59E-05 | H |  |
| 611 | 0.025116 | T |  |
| 681 | 0.00638 | L | KLELFTYLRF |
| 682 | 0.000659 | E | KLELFTYLRF |
| 683 | 0.000326 | L | KLELFTYLRF |
| 721 | 0.012965 | V | ILTHQIMYV |
| 848 | 8.74E-05 | S |  |
