# Supplementary material for: In silico and ex vivo approaches indicate immune pressure on capsid and non-capsid regions of coxsackie B viruses in the human system
Source: PLoS One. 2018 Jun 20;13(6):e0199323. doi: 10.1371/journal.pone.0199323 (PMC6010236; doi:10.1371/journal.pone.0199323)
Supplement: S5 Table — Full length sequences returned from Genbank for the queries “CBV3”, “CVB3”, “Coxsackievirus B3” and “Coxsackie B Virus 3” and used to identify sites evolving under positive selective pressure using Datamonkey’s mixed effects model of evolution packages. Sites that were identified as evolving under positive selection are listed, along with HLA-A*02:01 binding epitopes that incorporate that site. (DOCX) [file pone.0199323.s006.docx]

S5 Table: Sites of Positive Selection Identified in CBV3 by MEME.

This analysis was performed using all CBV3 sequences listed in S1 Table.

| Codon | Component | p-value | Consensus Identity | HLA-A2 Epitope |
| --- | --- | --- | --- | --- |
| 16 | VP4 | 0.004 | G | TGAHETGLNA |
| 18 | VP4 | 0.011092 | N | TGAHETGLNA |
| 30 | VP4 | 0.03321 | I | NSIIHYTNIN |
| 82 | NC | 0.002052 | X |  |
| 177 | VP2 | 0.001333 | X | YLGRTGYTIHV |
| 213 | VP2 | 0.019245 | A |  |
| 220 | VP2 | 4.03E-08 | X |  |
| 235 | VP2 | 0.043209 | K |  |
| 366 | NC | 0.002275 | M |  |
| 368 | NC | 0.018396 | I |  |
| 512 | VP3 | 0.000162 | A |  |
| 564 | VP3 | 0.027881 | S | LLKDTPFISQ |
| 566 | VP3 | 0.00014 | X |  |
| 648 | VP1 | 0.050696 | E |  |
| 661 | VP1 | 0.049235 | V |  |
| 662 | VP1 | 0.018929 | X |  |
| 829 | - | 0.023466 | K |  |
| 834 | - | 0.000675 | Q |  |
| 855 | - | 0.031457 | G |  |
| 861 | - | 0.006298 | V |  |
| 868 | 2A | 0.054301 | V |  |
| 873 | 2A | 0.000975 | L |  |
| 898 | 2A | 0.018232 | A |  |
| 923 | 2A | 4.14E-05 | Y |  |
| 968 | 2A | 0.058642 | E | GILRCEHGV |
| 1111 | - | 0.004514 | M |  |
| 1445 | 3A | 0.020592 | T |  |
| 1486 | 3A | 0.008391 | H |  |
| 1557 | c3 | 0.040698 | T | AMMKRNSSTV |
| 1824 | - | 0.003156 | V |  |
| 1888 | 3D | 0.054065 | X |  |
| 1921 | 3D | 0.033456 | F | AFHQNPGIVT |
| 1949 | 3D | 0.012551 | D | LDGHLIAFD |
| 2095 | 3D | 0.052197 | V |  |
| 2175 | - | 0.049761 | S |  |
| 2178 | - | 0.05063 | R |  |
