# Supplementary material for: In silico and ex vivo approaches indicate immune pressure on capsid and non-capsid regions of coxsackie B viruses in the human system
Source: PLoS One. 2018 Jun 20;13(6):e0199323. doi: 10.1371/journal.pone.0199323 (PMC6010236; doi:10.1371/journal.pone.0199323)
Supplement: S6 Table — Cryopreserved PBMCS were thawed and precultured at high-density for 48 hours as preparation for ELISpot assay. PBMCs were then recounted and plated at 3.3x10^6^/ml and 1x10^6^ cells stimulated with a pool of 4 peptides at 5μg/ml each final concentration (total final peptide concentration 20μg/ml) alongside diluent alone and viral peptide mix CEF (Mabtech) control conditions for three hours. Samples were transferred in triplicate to pre-coated and blocked IFNγ ELISpot plates (U-Cytech) and incubated for 24 hours. Cytokine release was identified as per the manufacturer’s instructions and plates counted using the Bio-sys Bioreader. The mean IFNy SI per 3.3x10^5^ cells of three replicate wells and total spots per 106 are presented for ELISpot assays against viral component specific peptide pools. (DOCX) [file pone.0199323.s007.docx]

**S6 Table: Raw Data for Viral Component Pool ELISpot Assays.**

|  |  | DMSO | VP4 | VP2 | VP3 | VP1 | 2A | 2B | 2C | 3A | C3 | 3D | CEF |
| --- | --- | --- | --- | --- | --- | --- | --- | --- | --- | --- | --- | --- | --- |
| Ctrl 1 | Mean SI | 1.0 | 0.5 | 0.4 | 0.2 | 1.0 | 0.8 | 0.9 | 0.4 | 0.4 | 0.4 | 0.1 | 12.1 |
|  | Total Spots | 28.0 | 15.0 | 10.0 | 5.0 | 27.0 | 22.0 | 25.0 | 12.0 | 12.0 | 10.0 | 3.0 | 339.0 |
| Ctrl 2 | Mean SI | 0.0 | 0.0 | 0.0 | 0.3 | 0.0 | 0.3 | 0.3 | 0.0 | 0.0 | 0.0 | 0.0 | 9.0 |
|  | Total Spots | 0.0 | 0.0 | 0.0 | 1.0 | 0.0 | 1.0 | 1.0 | 0.0 | 0.0 | 0.0 | 0.0 | 27.0 |
| Ctrl 3 | Mean SI | 0.0 | 0.0 | 0.7 | 0.3 | 0.7 | 1.0 | 0.3 | 0.3 | 0.0 | 0.0 | 0.3 | 3.7 |
|  | Total Spots | 0.0 | 0.0 | 2.0 | 1.0 | 2.0 | 3.0 | 1.0 | 1.0 | 0.0 | 0.0 | 1.0 | 11.0 |
| Ctrl 4 | Mean SI | 0.0 | 0.0 | 0.3 | 0.0 | 0.0 | 0.3 | 0.3 | 0.3 | 0.3 | 0.3 | 0.0 | 39.3 |
|  | Total Spots | 0.0 | 0.0 | 1.0 | 0.0 | 0.0 | 1.0 | 1.0 | 1.0 | 1.0 | 1.0 | 0.0 | 118.0 |
| Ctrl 5 | Mean SI | 0.0 | 0.0 | 0.3 | 0.0 | 0.3 | 0.7 | 0.0 | 0.3 | 0.3 | 0.0 | 0.0 | 19.0 |
|  | Total Spots | 0.0 | 0.0 | 1.0 | 0.0 | 1.0 | 2.0 | 0.0 | 1.0 | 1.0 | 0.0 | 0.0 | 57.0 |
| Ctrl 6 | Mean SI | 1.0 | 1.0 | 1.0 | 1.4 | 0.7 | 0.9 | 1.2 | 0.4 | 1.0 | 0.6 | 0.7 | 13.7 |
|  | Total Spots | 10.0 | 10.0 | 10.0 | 14.0 | 7.0 | 9.0 | 12.0 | 4.0 | 10.0 | 6.0 | 7.0 | 137.0 |
| Ctrl 7 | Mean SI | 0.0 | 0.3 | 1.3 | 0.0 | 0.3 | 0.0 | 1.3 | 0.7 | 0.0 | 0.3 | 0.3 | 7.3 |
|  | Total Spots | 0.0 | 1.0 | 4.0 | 0.0 | 1.0 | 0.0 | 4.0 | 2.0 | 0.0 | 1.0 | 1.0 | 22.0 |
| Ctrl 8 | Mean SI | 0.3 | 0.0 | 0.0 | 0.0 | 0.0 | 0.0 | 0.0 | 0.0 | 0.0 | 0.0 | 0.0 | 26.0 |
|  | Total Spots | 1.0 | 0.0 | 0.0 | 0.0 | 0.0 | 0.0 | 0.0 | 0.0 | 0.0 | 0.0 | 0.0 | 78.0 |
| Ctrl 9 | Mean SI | 0.0 | 0.3 | 0.3 | 0.0 | 0.0 | 0.0 | 0.3 | 0.0 | 0.0 | 0.0 | 0.7 | 49.7 |
|  | Total Spots | 0.0 | 1.0 | 1.0 | 0.0 | 0.0 | 0.0 | 1.0 | 0.0 | 0.0 | 0.0 | 2.0 | 149.0 |
| Ctrl 10 | Mean SI | 1.0 | 0.6 | 1.2 | 0.9 | 0.6 | 0.8 | 0.8 | 1.0 | 0.9 | 1.3 | 1.0 | 7.5 |
|  | Total Spots | 10.0 | 6.0 | 13.0 | 9.0 | 6.0 | 8.0 | 8.0 | 10.0 | 9.0 | 14.0 | 11.0 | 79.0 |
| Ctrl 11 | Mean SI | 1.0 | 0.5 | 0.2 | 2.0 | 1.0 | 1.7 | 1.3 | 1.2 | 0.5 | 0.7 | 0.5 | 19.7 |
|  | Total Spots | 6.0 | 3.0 | 1.0 | 12.0 | 6.0 | 10.0 | 8.0 | 7.0 | 3.0 | 4.0 | 3.0 | 118.0 |
| Ctrl 12 | Mean SI | 0.7 | 1.0 | 0.3 | 0.7 | 0.7 | 1.3 | 2.7 | 1.0 | 0.3 | 0.3 | 1.0 | 106.0 |
|  | Total Spots | 2.0 | 3.0 | 1.0 | 2.0 | 2.0 | 4.0 | 8.0 | 3.0 | 1.0 | 1.0 | 3.0 | 318.0 |
| Ctrl 13 | Mean SI | 0.0 | 0.3 | 1.0 | 1.3 | 1.0 | 0.0 | 0.0 | 0.7 | 0.3 | 1.3 | 2.0 | 9.0 |
|  | Total Spots | 0.0 | 1.0 | 3.0 | 4.0 | 3.0 | 0.0 | 0.0 | 2.0 | 1.0 | 4.0 | 6.0 | 27.0 |
| Ctrl 14 | Mean SI | 0.3 | 3.0 | 0.7 | 2.3 | 0.3 | 0.0 | 9.3 | 0.0 | 0.7 | 0.0 | 1.3 | 36.3 |
|  | Total Spots | 1.0 | 9.0 | 2.0 | 7.0 | 1.0 | 0.0 | 28.0 | 0.0 | 2.0 | 0.0 | 4.0 | 109.0 |
| Ctrl 15 | Mean SI | 0.3 | 0.3 | 0.0 | 0.0 | 0.3 | 0.0 | 0.0 | 0.0 | 0.0 | 0.0 | 0.0 | 27.3 |
|  | Total Spots | 1.0 | 1.0 | 0.0 | 0.0 | 1.0 | 0.0 | 0.0 | 0.0 | 0.0 | 0.0 | 0.0 | 82.0 |
| Ctrl 16 | Mean SI | 1.0 | 0.4 | 0.4 | 0.7 | 0.0 | 0.7 | 1.4 | 0.7 | 0.0 | 0.0 | 0.0 | 21.1 |
|  | Total Spots | 7.0 | 3.0 | 3.0 | 5.0 | 0.0 | 5.0 | 10.0 | 5.0 | 0.0 | 0.0 | 0.0 | 148.0 |
| Ctrl 17 | Mean SI | 1.0 | 1.3 | 2.3 | 1.3 | 2.3 | 0.3 | 1.0 | 1.0 | 1.0 | 1.7 | 1.7 | 33.3 |
|  | Total Spots | 3.0 | 4.0 | 7.0 | 4.0 | 7.0 | 1.0 | 3.0 | 3.0 | 3.0 | 5.0 | 5.0 | 100.0 |
| Ctrl 18 | Mean SI | 0.7 | 0.7 | 1.0 | 0.7 | 1.7 | 0.3 | 1.0 | 0.7 | 0.0 | 0.3 | 0.7 | 43.3 |
|  | Total Spots | 2.0 | 2.0 | 3.0 | 2.0 | 5.0 | 1.0 | 3.0 | 2.0 | 0.0 | 1.0 | 2.0 | 130.0 |
| T1D 1 | Mean SI | 0.0 | 0.0 | 0.0 | 0.0 | 0.0 | 0.0 | 0.0 | 0.0 | 0.0 | 0.0 | 0.3 | 14.0 |
|  | Total Spots | 0.0 | 0.0 | 0.0 | 0.0 | 0.0 | 0.0 | 0.0 | 0.0 | 0.0 | 0.0 | 1.0 | 42.0 |
| T1D 2 | Mean SI | 0.0 | 1.0 | 0.3 | 0.3 | 0.3 | 0.0 | 1.0 | 0.0 | 0.0 | 0.0 | 0.0 | 11.0 |
|  | Total Spots | 0.0 | 3.0 | 1.0 | 1.0 | 1.0 | 0.0 | 3.0 | 0.0 | 0.0 | 0.0 | 0.0 | 33.0 |
| T1D 3 | Mean SI | 1.0 | 2.3 | 0.7 | 2.0 | 1.0 | 0.7 | 2.7 | 1.3 | 0.3 | 2.3 | 1.0 | 54.7 |
|  | Total Spots | 3.0 | 7.0 | 2.0 | 6.0 | 3.0 | 2.0 | 7.0 | 4.0 | 1.0 | 7.0 | 3.0 | 164.0 |
| T1D 4 | Mean SI | 0.0 | 0.0 | 0.0 | 0.3 | 0.3 | 1.3 | 0.0 | 0.0 | 0.0 | 0.0 | 0.0 | 41.7 |
|  | Total Spots | 0.0 | 0.0 | 0.0 | 1.0 | 1.0 | 4.0 | 0.0 | 0.0 | 0.0 | 0.0 | 0.0 | 125.0 |
| T1D 5 | Mean SI | 1.0 | 1.1 | 0.8 | 0.8 | 1.2 | 1.8 | 2.0 | 0.4 | 0.9 | 0.7 | 0.8 | 5.9 |
|  | Total Spots | 44.0 | 47.0 | 35.0 | 35.0 | 54.0 | 77.0 | 87.0 | 18.0 | 40.0 | 29.0 | 37.0 | 258.0 |
| T1D6 | Mean SI | 0.3 | 0.0 | 0.0 | 0.7 | 0.0 | 0.0 | 0.0 | 0.3 | 0.0 | 0.0 | 0.7 | 3.7 |
|  | Total Spots | 1.0 | 0.0 | 0.0 | 2.0 | 0.0 | 0.0 | 0.0 | 1.0 | 0.0 | 0.0 | 2.0 | 11.0 |
| T1D7 | Mean SI | 0.0 | 0.0 | 0.3 | 0.3 | 1.0 | 0.0 | 0.3 | 0.3 | 0.7 | 0.7 | 1.0 | 16.0 |
|  | Total Spots | 0.0 | 0.0 | 1.0 | 1.0 | 3.0 | 0.0 | 1.0 | 1.0 | 2.0 | 2.0 | 3.0 | 48.0 |
| T1D8 | Mean SI | 0.3 | 0.0 | 0.3 | 0.0 | 0.3 | 1.0 | 0.3 | 1.3 | 0.0 | 0.0 | 0.0 | 3.7 |
|  | Total Spots | 1.0 | 0.0 | 1.0 | 0.0 | 1.0 | 3.0 | 1.0 | 4.0 | 0.0 | 0.0 | 0.0 | 11.0 |
| T1D9 | Mean SI | 1.0 | 0.5 | 0.8 | 1.1 | 0.6 | 0.7 | 0.5 | 0.3 | 0.5 | 0.5 | 0.2 | 12.0 |
|  | Total Spots | 10.0 | 5.0 | 8.0 | 11.0 | 6.0 | 7.0 | 5.0 | 3.0 | 5.0 | 5.0 | 2.0 | 120.0 |
| T1D 10 | Mean SI | 0.0 | 0.0 | 0.0 | 0.7 | 0.3 | 0.0 | 0.0 | 0.0 | 1.0 | 0.3 | 0.3 | 107.7 |
|  | Total Spots | 0.0 | 0.0 | 0.0 | 2.0 | 1.0 | 0.0 | 0.0 | 0.0 | 3.0 | 1.0 | 1.0 | 323.0 |
| T1D 11 | Mean SI | 0.0 | 0.7 | 0.0 | 0.0 | 0.0 | 0.3 | 1.0 | 0.3 | 0.0 | 0.0 | 0.3 | 59.3 |
|  | Total Spots | 0.0 | 2.0 | 0.0 | 0.0 | 0.0 | 1.0 | 3.0 | 1.0 | 0.0 | 0.0 | 1.0 | 178.0 |
| T1D 12 | Mean SI | 0.3 | 0.3 | 0.0 | 0.7 | 0.0 | 0.3 | 0.7 | 0.7 | 0.0 | 0.0 | 0.0 | 4.7 |
|  | Total Spots | 1.0 | 1.0 | 0.0 | 2.0 | 0.0 | 1.0 | 2.0 | 2.0 | 0.0 | 0.0 | 0.0 | 14.0 |
| T1D 13 | Mean SI | 1.0 | 0.7 | 3.0 | 9.0 | 0.7 | 0.0 | 0.3 | 0.3 | 0.7 | 1.0 | 0.3 | 112.0 |
|  | Total Spots | 3.0 | 2.0 | 9.0 | 27.0 | 2.0 | 0.0 | 1.0 | 1.0 | 2.0 | 3.0 | 1.0 | 336.0 |
| T1D 14 | Mean SI | 0.3 | 0.3 | 1.0 | 1.7 | 1.3 | 0.0 | 0.0 | 0.7 | 0.3 | 0.3 | 1.0 | 12.7 |
|  | Total Spots | 1.0 | 1.0 | 3.0 | 5.0 | 4.0 | 0.0 | 0.0 | 2.0 | 1.0 | 1.0 | 3.0 | 38.0 |
| T1D 15 | Mean SI | 1.0 | 0.7 | 3.0 | 0.0 | 0.3 | 2.0 | 0.7 | 0.3 | 0.7 | 0.7 | 1.0 | 15.3 |
|  | Total Spots | 3.0 | 2.0 | 9.0 | 0.0 | 1.0 | 6.0 | 2.0 | 1.0 | 2.0 | 2.0 | 3.0 | 46.0 |
| T1D 16 | Mean SI | 0.3 | 0.3 | 0.0 | 0.3 | 0.0 | 0.0 | 1.3 | 0.0 | 0.0 | 0.3 | 0.0 | 29.3 |
|  | Total Spots | 1.0 | 1.0 | 0.0 | 1.0 | 0.0 | 0.0 | 4.0 | 0.0 | 0.0 | 1.0 | 0.0 | 88.0 |
